# Transcriptomic Analysis of Vitrified–Warmed vs. Fresh Mouse Blastocysts: Cryo-Induced Physiological Mechanisms and Implantation Impact

**DOI:** 10.3390/ijms25168658

**Published:** 2024-08-08

**Authors:** Chi-Ying Lee, Han-Ni Tsai, En-Hui Cheng, Tsung-Hsien Lee, Pin-Yao Lin, Maw-Sheng Lee, Chun-I Lee

**Affiliations:** 1 Genetic Diagnosis Laboratory, Lee Women’s Hospital, Taichung 40652, Taiwan; s110080811@m110.nthu.edu.tw (C.-Y.L.); pin36.tw@yahoo.com.tw (H.-N.T.); enhuicheng@gmail.com (E.-H.C.); 2 Institute of Bioinformatics and Structural Biology, National Tsing Hua University, Hsinchu 30013, Taiwan; 3 Post Baccalaureate Medicine, National Chung Hsing University, Taichung 40227, Taiwan; jellylin0607@gmail.com; 4 Division of Infertility, Lee Women’s Hospital, Taichung 40402, Taiwan; t01617@live.csmu.edu.tw (T.-H.L.); msleephd@csmu.edu.tw (M.-S.L.); 5 Institute of Medicine, Chung Shan Medical University, Taichung 40201, Taiwan; 6 Department of Obstetrics and Gynecology, Chung Shan Medical University Hospital, Taichung 40201, Taiwan; 7 Department of Obstetrics and Gynecology, School of Medicine, Chung Shan Medical University, Taichung 40201, Taiwan

**Keywords:** blastocyst, vitrification, transcriptome, gene expression, implantation, microRNAs, next-generation sequencing, frozen embryo transfer, assisted reproduction

## Abstract

Blastocyst vitrification has significantly improved embryo transfer methods, leading to higher implantation success rates and better pregnancy outcomes in subsequent frozen embryo transfer cycles. This study aimed to simulate the transcriptional changes caused by vitrifying human blastocysts using mouse blastocysts as a model and to further investigate these changes’ effects. Utilizing a human vitrification protocol, we implanted both vitrified and fresh embryos into mice. We observed the implantation success rates and performed transcriptomic analysis on the blastocysts. To validate the results from messenger RNA sequencing, we conducted reverse transcriptase-quantitative polymerase chain reaction (RT-qPCR) to measure the expression levels of specific genes. Based on mRNA profiling, we predicted the microRNAs responsible for the regulation and used qPCR basic microRNA assays for validation. Our observations revealed a higher implantation success rate for vitrified embryos than fresh embryos. Transcriptomic analysis showed that vitrified–warmed blastocysts exhibited differentially expressed genes (DEGs) primarily associated with thermogenesis, chemical carcinogenesis-reactive oxygen species, oxidative phosphorylation, immune response, and MAPK-related signaling pathways. RT-qPCR confirmed increased expression of genes such as *Cdk6* and *Nfat2*, and decreased expression of genes such as *Dkk3* and *Mapk10*. Additionally, gene-microRNA interaction predictions and microRNA expression analysis identified twelve microRNAs with expression patterns consistent with the predicted results, suggesting potential roles in uterine epithelial cell adhesion, trophectoderm development, invasive capacity, and immune responses. Our findings suggest that vitrification induces transcriptomic changes in mouse blastocysts, and even small changes in gene expression can enhance implantation success. These results highlight the importance of understanding the molecular mechanisms underlying vitrification to optimize embryo transfer techniques and improve pregnancy outcomes.

## 1. Introduction

Vitrification, a cryopreservation technique that significantly enhances the flexibility of in vitro fertilization (IVF) procedures, allows for the efficient preservation of embryos before their transfer in subsequent menstrual cycles. While some studies have suggested that blastocyst vitrification improves perinatal outcomes, such as higher newborn weights [1,2,3], others indicate no significant differences [4]. Factors influencing embryo implantation include synchronization between endometrial maturation and the transferred embryos, female age, uterine condition, and embryonic factors. However, the impact of blastocyst vitrification and warming on embryo implantation and clinical outcomes remains to be fully elucidated.

This study aims to investigate the stress-induced physiological changes in mouse embryos caused by vitrification and warming through comprehensive transcriptomics and in silico analysis. The altered messenger RNA (mRNA) and microRNA profiles will contribute to our understanding and assessment of the impacts caused by the freeze-thaw process. Transcriptomic profiling of preimplantation embryos can identify genes that characterize this developmental stage, providing potential markers and insights into their functional features [5]. Nevertheless, the small and rapid changes in the embryo transcriptome pose significant research challenges.

Transcriptomic analysis involves RNA sequencing or microarray techniques to identify and quantify the expression levels of RNA transcripts, including messenger RNA (mRNA) and microRNA. The interaction between microRNA and mRNA is a crucial mechanism for regulating gene expression, wherein specific microRNAs can recognize and bind to specific mRNA molecules based on complementary base pairing, leading to mRNA degradation or translation inhibition [6].

Previous studies have employed various vitrification protocols [7,8,9,10]. We aim to extend the transcriptomics analysis results using in silico approaches to establish connections with the final implantation outcomes. This approach may provide potential explanations for the stress responses induced in embryos by the freeze-thaw process. In contrast, our study utilizes clinically relevant human vitrification protocols to detect gene expression profiles in a mouse model. It has been suggested that blastocysts may interact with the endometrium through microRNA and coordinate gene expression during the peri-implantation period [11]. Blastocyst vitrification alters the microRNA profile [12]; however, the relationship between the altered microRNA and mRNA profiles remains uncertain.

By employing mRNA sequencing for comprehensive transcriptomic analysis and in silico analysis, our study investigates the effects of blastocyst vitrification on mRNA expression and aims to elucidate how changes in gene expression in vitrified–warmed embryos might influence blastocyst implantation and subsequent fetal development. By employing mRNA sequencing for comprehensive transcriptomic analysis, we investigate the effects of blastocyst vitrification on mRNA expression and explore the interaction between microRNA and mRNA in this context. Our approach utilizes clinically relevant human vitrification protocols in a mouse model, enhancing the translational relevance of our findings.

While previous studies have separately indicated the impact of mRNA and microRNA on embryo implantation [7,11], a comprehensive investigation into the effects of human vitrification freezing protocols on the interplay between these two molecular profiles is lacking. Our study seeks to examine transcriptome changes and identify potential functional alterations comprehensively, offering insights into the complex interplay between microRNA regulation and gene expression in the context of embryo viability and implantation potential. Elucidating these functional changes is crucial for understanding how they may influence blastocyst development and embryo implantation. This research aims to contribute to the development of more effective vitrification protocols and potentially improve the success rates of assisted reproductive technologies, addressing significant challenges in the field of reproductive medicine.

## 2. Results

### 2.1. Impact of Vitrified–Warmed Mouse Blastocysts on Implantation

In this stage, we evaluated whether vitrified–warmed mouse blastocysts would affect implantation. Sixty embryos were randomly transferred, with thirty embryos subjected to vitrification-warming before transfer. To eliminate the potential impact of individual differences in mice on the implantation success rate, we transferred vitrified–warmed embryos into one side of the uterus and fresh embryos into the other side of the same mouse.

Mouse embryos were frozen using a clinical vitrification kit. Six vitrified–warmed and six fresh embryos were implanted into the right and left uterine horns of five mice, respectively. The implantation success rates for the vitrified–warmed and fresh embryos were 83.3% and 56.7%, respectively (*p* = 0.039; Table 1). Vitrification significantly increased the implantation success rate in this study.

### 2.2. Effect of Cryopreservation on Transcriptomic Profile

We investigated the impact of cryopreservation on the transcriptomic profile of in vivo–derived blastocysts. Initially, candidate genes were selected based on criteria including an average FPKM value of all samples > 0.5, a *p*-value < 0.05, and a 1.5-fold change between the case and control groups. The analysis utilized a list of differentially expressed genes (DEGs) generated from the selection criteria for more than 1.5-fold changes. In total, 2642 DEGs (1239 upregulated and 1403 downregulated genes) were identified in the vitrified–warmed blastocysts (Supplementary Table S1). Due to the limited amount of mRNA in each sample and the embryos being in a stage of rapid development, several gene expressions were found to be exclusive to either the case or control cells.

### 2.3. Gene Ontology Enrichment Analysis in Vitrified–Warmed and Fresh Preimplantation Embryos

Gene ontology (GO) enrichment analysis [13] was conducted to identify the biological processes, cellular components, and molecular functions associated with the DEG. The selection criteria for enriched GO terms included a gene count ≥ 3, a false discovery rate < 0.01, and an enrichment fold-change ≥ 1.5.

Compared to fresh blastocysts, the upregulated DEGs in vitrified–warmed blastocysts were enriched for 32 GO terms, while the downregulated DEGs were enriched for 45 GO terms (Supplementary Table S2). Notably, among the upregulated DEGs, no molecular function terms were enriched; however, their enrichment fold-changes were considerably higher, with a maximum approaching 6-fold. In contrast, none of the enrichment fold-changes for the downregulated DEGs exceeded 3-fold.

**Figure 1:**
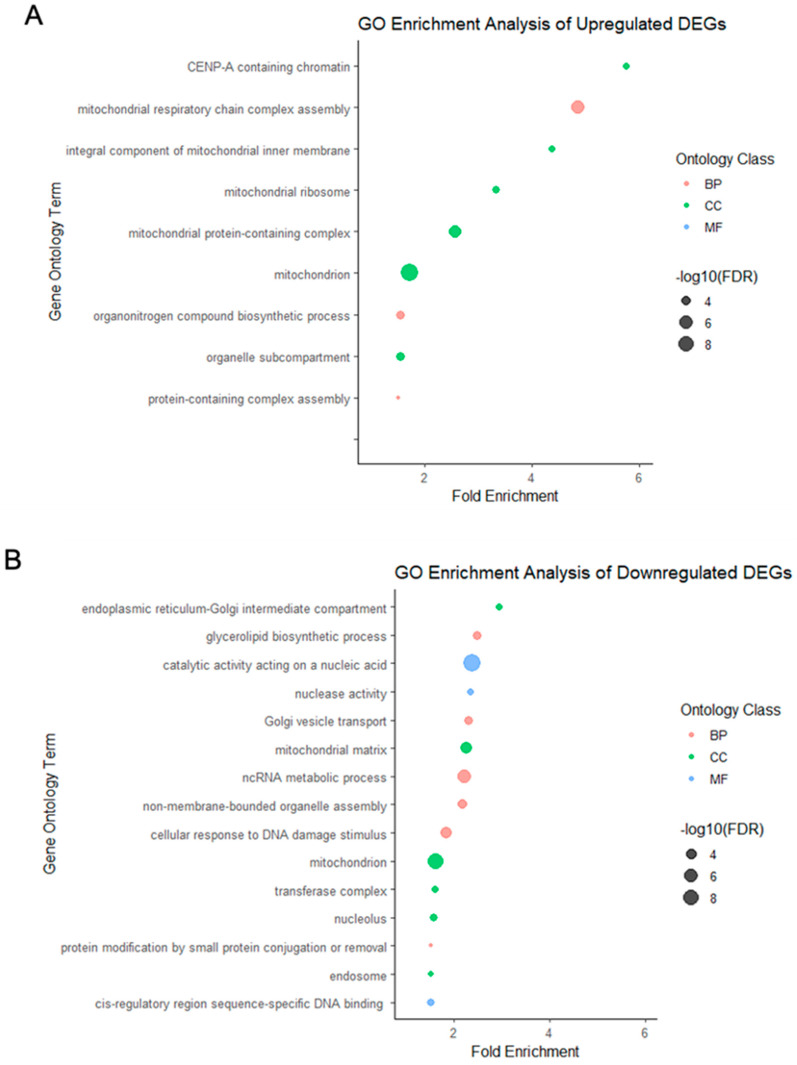
The most abundant GO terms correspond to the (**A**) upregulated and (**B**) downregulated gene expression of vitrified–warmed blastocysts. A plot of gene characteristics depicts the functional categories derived from molecular functions, biological processes, and cellular components. The GO enrichment analysis reveals critical insights into the physiological impacts of vitrification and warming on mouse blastocysts. The upregulated DEGs, highly associated with mitochondria and chromatin, suggest enhanced metabolic activity and potential chromatin remodeling. In contrast, the downregulated DEGs, linked to the endoplasmic reticulum-Golgi intermediate compartment and glycerolipid biosynthesis, indicate a reduction in lipid metabolism and intracellular transport processes. These findings highlight the distinct molecular adaptations occurring in response to cryopreservation, providing a deeper understanding of its effects on embryo viability and development potential. Larger data points indicate lower FDR. Red, green, and blue dots represent biological processes, cellular components, and molecular functions, respectively.

The DEGs in the vitrified–warmed blastocysts were categorized into distinct functional groups based on their molecular functions, biological processes, and cellular components. Hierarchical clustering analysis was applied to the various functional categories for both up and downregulated DEGs. If GO terms shared more than 50% of genes, they were combined, and the most statistically significant term was selected (Figure 1).

GO enrichment analysis revealed that the upregulated DEGs were primarily associated with mitochondria and chromatin, whereas the downregulated DEGs were primarily associated with the endoplasmic reticulum-Golgi intermediate compartment, glycerolipid biosynthetic processes, and catalytic activity acting on nucleic acids, occupying the top three positions in terms of enrichment fold-changes.

### 2.4. Kyoto Encyclopedia of Genes and Genomes Pathway Enrichment Analysis

**Figure 2:**
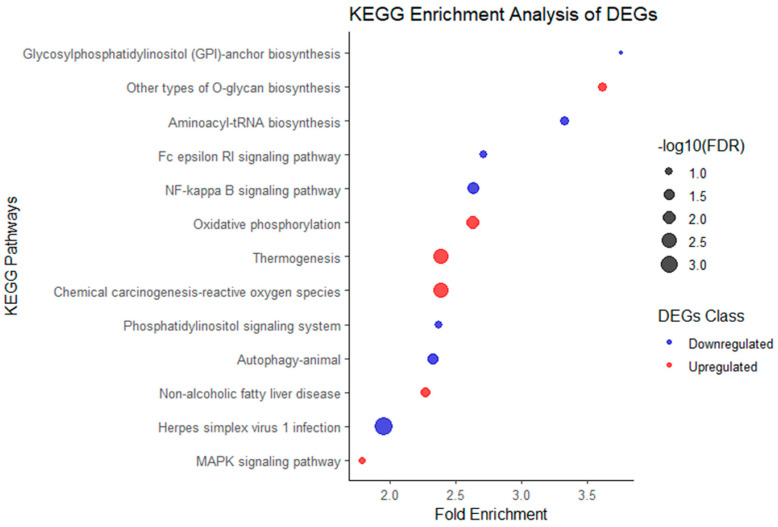
Enrichment analysis of KEGG pathways associated with DEGs in vitrified–warmed blastocysts. The scatterplot depicts the enriched KEGG pathways corresponding to upregulated (red) and downregulated (blue) DEGs. With a false discovery rate (FDR) < 0.2 and fold-change ≥ 1.5, the upregulated DEGs were enriched for 6 pathways, while the downregulated DEGs were enriched for 7 pathways. Enriched pathways of upregulated DEGs such as “Thermogenesis”, “Chemical carcinogenesis-reactive oxygen species”, and “Oxidative phosphorylation”. These pathways promote ATP generation, affecting cell survival, proliferation, migration, and differentiation via the “MAPK signaling pathway”. Enriched pathways of downregulated DEGs mostly involved in immune responses, such as “Herpes simplex virus 1 infection”, “NF-kappa B signaling pathway”, “Autophagy-animal”, “Fc epsilon RI signaling pathway”, and “Glycosylphosphatidylinositol (GPI)-anchor biosynthesis”. The x-axis represents the fold enrichment, calculated as the ratio of the observed gene frequency in the pathway to the expected frequency, based on a random distribution. The y-axis represents the −log10 transformed false discovery rate (FDR), where larger dot points indicate lower FDR values and higher statistical significance. Pathways positioned towards the left exhibit more significant fold enrichments, indicating a higher representation of DEGs in those pathways compared to random expectation.

The upregulated and downregulated genes in vitrified–warmed blastocysts were analyzed to investigate their associations with the critical Kyoto Encyclopedia of Genes and Genomes (KEGG) pathways (Supplementary Table S3). With a false discovery rate (FDR) < 0.2 and fold-change ≥ 1.5, the upregulated DEGs were enriched for six pathways, while the downregulated DEGs were enriched for seven pathways (Figure 2).

**Figure 3:**
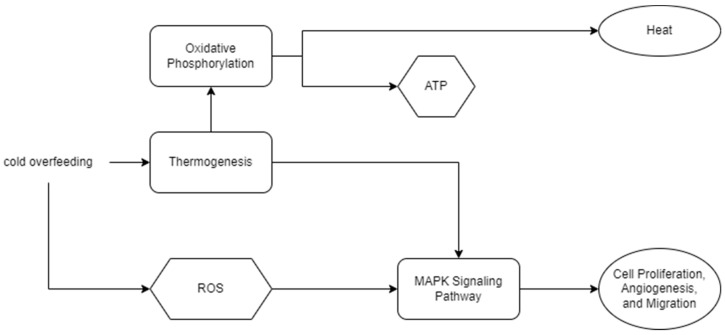
Physiological Mechanisms Induced by Cryopreservation. Cold overfeeding leads to cellular damage, prompting the thermogenesis process to generate heat. This process activates downstream pathways, such as “Oxidative phosphorylation” and “MAPK signaling pathway,” which are essential for ATP production and cellular functions. Consequently, the cryopreservation and warming process necessitates increased ATP production to sustain cell survival and proliferation. The KEGG database indicates associations between pathway enrichment results, represented as rectangles, and downstream mechanism predictions, depicted as ovals. Hexagons denote vital compounds.

Some pathways enriched by upregulated DEGs exhibited clear upstream and downstream relationships. For instance, the upstream pathways “Thermogenesis” (mmu04714) and “Chemical carcinogenesis-reactive oxygen species” (mmu05208) lead to “Oxidative phosphorylation” (mmu00190), promoting ATP generation within cells, ultimately affecting cell survival, proliferation, migration, and differentiation via the “MAPK signaling pathway” (mmu04010). These pathway associations are based on inferences and suggestions provided by KEGG (Figure 3).

Interestingly, the pathways enriched by downregulated DEGs were mostly directly or indirectly involved in immune responses, such as “Herpes simplex virus 1 infection” (mmu05168), “NF-kappa B signaling pathway” (mmu04064), “Autophagy-animal” (mmu04140), “Fc epsilon RI signaling pathway” (mmu04664), and “Glycosylphosphatidylinositol (GPI)-anchor biosynthesis” (mmu00563).

### 2.5. RT-qPCR Validation of Key Genes

Based on the previous pathway enrichment analysis, we selected key genes related to cell cycle regulation for validation by reverse transcription-quantitative polymerase chain reaction (RT-qPCR).

**Figure 4:**
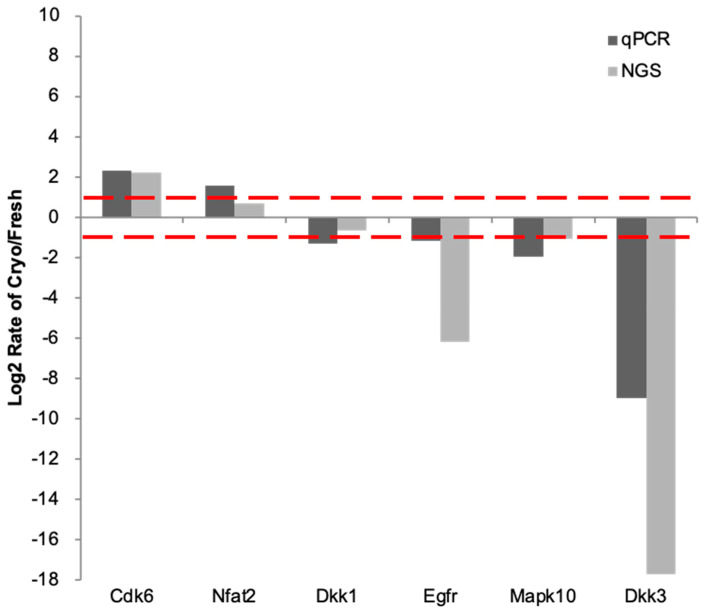
Validation of gene expression results by RT-qPCR. The Y-axis represents the log-2-fold change between vitrified–warmed and fresh blastocysts. The dark gray bar represents the ratio generated by RT-qPCR, and the light gray bar represents the ratio calculated by the NGS result. The dashed line represents the two-fold change difference between the vitrified and fresh cells.

The six validated genes exhibited expression trends consistent with the RNA-Seq data (Figure 4). RT-qPCR analysis demonstrated upregulated mRNA expression of *Cdk6* and *Nfat2*, whereas *Dkk3*, *Mapk10*, and *Egfr* mRNA levels were downregulated. Additionally, the expression pattern of *Dkk1* corroborated the RNA-Seq findings. The gene expression differences between vitrified and fresh embryos analyzed through RT-qPCR were concordant with the RNA-Seq results, indicating the reliability of the transcriptomic data.

### 2.6. Prediction of MicroRNA Involvement in Vitrified–Warmed Embryos

microRNAs are small noncoding RNAs, approximately 21–23 nucleotides in length, that play a crucial role in regulating gene expression by binding to target messenger RNAs (mRNAs). We employed bioinformatics tools to predict which microRNAs may be implicated in vitrified–warmed embryos based on the observed mRNA expression profiles.

We analyzed the entire gene list using GSEA v.4.1.0 [14,15] to extract putative microRNAs. The predicted microRNA expression trend decreased if the enrichment score was negative and increased if the score was positive. Our analysis identified the enrichment of 536 microRNAs with a *p*-value of 0.05 in vitrified–warmed blastocysts (Supplementary Table S5).

### 2.7. Identification and Functional Analysis of Validated MicroRNA Candidates in Vitrified–Warmed Embryos

The miRCURY experimental analysis, with a fold-change cutoff of 1.5 and a quality threshold exceeding level “B”, identified 97 out of 768 tested microRNAs meeting these criteria (Supplementary Table S6). By intersecting the predicted and experimental microRNA datasets, we identified 14 potential microRNA candidates. However, only 12 of these microRNAs exhibited consistent expression trends between the predicted and experimental results (Table 2), validating the performance trends of the predicted microRNA candidates in vitrified embryos.

To investigate the potential roles of these validated microRNA candidates in vitrified embryos, we extracted the target genes of the 12 microRNAs for functional enrichment analysis. This analysis provided insights into the interactions between microRNAs and their target messenger RNAs (mRNAs) in vitrified–warmed embryos (Table 3).

## 3. Discussion

While previous studies have reported differential transcriptional or microRNA expression in vitrified–warmed blastocysts compared to fresh blastocysts, the correlation between these two molecular profiles has not been comprehensively explored. The present study aimed to simultaneously analyze the transcriptome and microRNA expression in vitrified–warmed blastocysts using RNA-sequencing and microRNA profiling, respectively, to elucidate the potential molecular changes induced by the vitrification process.

To ensure clinical relevance, we performed vitrification of mouse blastocysts using a vitrification kit similar to those employed in human in vitro fertilization (IVF) procedures. Additionally, we conducted implantation assays to validate that the implantation mechanism in the mouse model closely resembles the human process. Specifically, six vitrified–warmed blastocysts were transferred into the right uterine horn of pseudopregnant female mice, while an equal number of fresh blastocysts were transferred into the left uterine horn of the same recipients. This experimental design accounted for potential differences in maternal and uterine synchrony.

Notably, our study revealed a higher implantation rate for vitrified blastocysts compared to fresh blastocysts. This significant difference in implantation rates aligns with some clinical retrospective studies [16], although others have shown no significant disparity [17,18]. It is crucial to acknowledge the species differences between humans and mice, as most studies in mice and other species have demonstrated slightly lower implantation rates for vitrified blastocysts [19]. While we cannot definitively conclude that the application of human clinical protocols to mouse embryos is responsible for these divergent results, our findings clearly demonstrate that the freeze-thaw process induces changes in mRNA and miRNA profiles.

This observation suggests that vitrified mouse blastocysts may exhibit a higher implantation success rate due to specific molecular changes induced by the vitrification process. To elucidate the mechanisms behind this phenomenon, we conducted a comprehensive transcriptomic analysis. Our aim was twofold: to confirm mRNA and miRNA alterations due to freeze-thaw processes in a stable model organism, and to investigate how these changes in blastocysts might influence implantation rates.

The discrepancy between our results and previous studies in mice underscores the complexity of cryopreservation effects on embryo viability and implantation potential. It highlights the need for a deeper understanding of the molecular mechanisms at play. Our transcriptomic analysis provides valuable insights into these mechanisms, potentially explaining the enhanced implantation success of vitrified blastocysts observed in our study.

In the following sections, we will delve into the specific transcriptomic changes observed in vitrified blastocysts compared to fresh ones. This analysis reveals alterations in key genetic pathways and regulatory networks that may contribute to the improved implantation rates. By examining these molecular changes in detail, we aim to shed light on the underlying biological processes affected by vitrification and their potential impact on embryo development and implantation success.

GO term enrichment analysis of DEGs in vitrified–warmed blastocysts revealed up-regulation of genes associated with mitochondrial functions and chromatin regulation, particularly those related to mitochondria and CENP-A-containing chromatin (Figure 1A). These findings indicate that the vitrification-warming process triggers blastocyst stress-related responses, with upregulated gene expressions primarily converging into these two cellular function categories. Conversely, the downregulated DEGs (Figure 1B) exhibited a more diverse pattern, encompassing categories, such as intracellular transport, glycerolipid biosynthetic processes, and catalytic activity acting on nucleic acids. This diversity underscores the complexity of gene expression changes induced by vitrification in blastocysts.

Inhibiting glycerolipid biosynthetic processes in bovine embryos produced in vitro has been shown to reduce lipid content, increase mitochondrial activity, and improve cryotolerance, while also favoring the expression of genes involved in lipid metabolism regulation and oxidative stress response [20]. Additionally, cryopreserved embryos may experience reductions in mitochondrial size, number, or functionality [21,22]. Although vitrification has a lesser impact on embryos than slow freezing, it still causes membrane damage, mitochondrial dysfunction, and DNA damage [23]. Our fold enrichment analysis revealed significant alterations in chromatin regulation and cellular mitochondrial function, supporting the involvement of these processes in the observed changes. The frequent mention of mitochondria-related terms underscores the importance of mitochondria as primary energy-producing organelles in vitrified–warmed blastocysts. Interestingly, our results suggest that mitochondrial function, DNA repair, and glycerolipid biosynthesis begin to recover at an early stage following vitrification and warming.

We characterized the molecular response of vitrified–warmed embryos through pathway enrichment analysis of DEGs. KEGG pathway enrichment analysis of upregulated DEGs indicated the enrichment of gene functions promoting thermogenesis, oxidative phosphorylation, and O-glycan biosynthesis in vitrified–warmed embryos. Specifically, enriched pathways included thermogenesis, chemical carcinogenesis-reactive oxygen species, oxidative phosphorylation, nonalcoholic fatty liver disease, other types of O-glycan biosynthesis, and the MAPK signaling pathway (Figure 2). These pathways collectively contribute to ATP production, cell proliferation, angiogenesis, and migration (Figure 3). Among the enriched pathways, thermogenesis exhibited the lowest false discovery rate (FDR), indicating the highest statistical significance. This suggests that, under cold stress, vitrified embryos attempt to enhance mitochondrial activity and increase ATP production for self-preservation. Previous studies have observed similar protective mechanisms in general cells and MII oocytes under cold conditions [24,25].

The downstream MAPK signaling pathway also showed a 1.78-fold gene enrichment. Upregulated DEGs included genes such as *Nfat2* and *Cdk6*, which regulate cell proliferation, differentiation, stability, and pregnancy by mediating adhesion molecules and angiogenesis [26,27]. In summary, vitrified–warmed embryos respond to cold stress, cellular damage, and increased reactive oxygen species (ROS) by enhancing thermogenesis, oxidative phosphorylation, and energy metabolism. Providing antioxidants or additional culture time could assist in restoring embryonic health [21,28,29]. Concurrently, the MAPK signaling pathway activation may influence subsequent embryonic cell proliferation or implantation.

We further validated key genes from the enriched biological pathways to confirm the existence of MAPK signaling pathway activation. Specifically, we established the upregulated expression of *Cdk6* and *Nfat2*, which are known to, through the MAPK pathway, promote cell proliferation, differentiation, and migration [30,31]. It has been observed that MAPK10 inhibits the phosphorylation and activity of NFAT2, suggesting that decreased MAPK10 levels may enhance NFAT2 activity [32]. DKK3, on the other hand, serves as an essential transcription factor regulatory protein, and a decrease in *Dkk3* gene expression may facilitate the entry of transcription factors into the nucleus [33]. These genes were selected for validation from the RNA-Seq data to confirm the predisposition of the cells towards proliferation, differentiation, and migration.

For the downregulated DEGs, KEGG pathway enrichment analysis showed that the herpes simplex virus 1 infection pathway had the lowest FDR, indicating the highest statistical significance. This pathway is associated with inflammatory factors. Downregulation of genes related to herpes simplex virus 1 infection, and the associated decrease in inflammatory factors, suggests a reduced immune response. This immune response reduction may mimic the maternal immune system’s tolerance of the blastocyst. Consequently, the gene expression signature of immune evasion in vitrified–warmed blastocysts may contribute to the observed higher implantation success rate. Literature suggests that NF-kappa B upregulation can regulate and inhibit apoptosis [34]. Additionally, decreased NF-kappa B signaling pathway activity, observed in the human endometrium and early decidua during pregnancy, contributes to successful embryo implantation [35]. The Fc epsilon RI signaling pathway and glycosylphosphatidylinositol (GPI)-anchor biosynthesis are also involved in immune regulation. Our study found that embryos exhibit immune regulation to facilitate better implantation. MicroRNAs in exosomes may assist in the implantation process [11], likely serving as a communication method between the early embryo and the uterus or maternal system, coordinating mechanisms to achieve implantation.

Previous studies have suggested that preimplantation embryos may utilize microRNAs to self-regulate their development and coordination with the maternal uterus for preimplantation preparation [11,12]. We aimed to quantify microRNAs to verify whether embryos indeed use microRNAs for self-regulation and immune coordination with the uterus. By predicting microRNA candidates based on altered mRNA expression, we identified 12 microRNAs that potentially regulate vitrified–warmed blastocysts (Table 2). These microRNAs involve various biological processes, such as focal adhesion, energy metabolism, tissue differentiation, transcriptional regulation, immune regulation, autophagy, and cation regulation [36]. In our study, these microRNAs primarily influence processes related to immune response, regulation of embryonic stem cells, and inflammation (Table 3).

The regulation of miR-200 family members has been previously observed during the embryo implantation process, with a natural decrease in their levels modulating estradiol and progesterone levels, while their targets, Zeb1 and Zeb2, naturally increase [37]. Overexpression of the miR-200 family has been shown to inhibit implantation success rates in mice [38]. Low miR-200b expression may enhance and induce the angiogenic response of endothelial cells. MiR-200b regulates focal adhesion and germ-layer specification in early embryos, with decreased expression observed during implantation [37,39,40]. MiR-93 localizes to the differentiating primitive endoderm and trophectoderm of the blastocyst, controlling stem cell differentiation [39,41]. Additionally, increased miR-93 levels in trophoblast cells cause apoptosis and inhibit proliferation, migration, and invasion, whereas decreased miR-93 levels can reverse these mechanisms [42]. MiR-150-5p impacts placental cellular abilities, including migration, invasion, and angiogenesis of extravillous trophoblast cells, and regulates VEGF and MMP9 expression [43]. Our observations of differentially expressed miR-200b, miR-93, and miR-150 in vitrified–warmed embryos are consistent with these findings and may contribute to the increased implantation success rates observed in our study.

Based on the functional enrichment classification of microRNAs, the “Immune Response” category includes miR-9, miR-103a, miR-150, miR-93, and miR-200b. The decrease in miR-9 may lead to immune evasion or a reduction in innate immunity, as miR-9 downregulates inflammation [44,45,46,47]. The decrease in miR-150 affects T-cell differentiation and cytotoxic efficiency [48,49]. Additionally, the reduced expression of miR-150 may decrease kinase activity and limit activated T-cell proliferation [50], contributing to uterine receptivity. Similarly, the downregulation of miR-93 promotes immune evasion, a phenomenon observed in cancer cells [51,52]. By regulating Kindlin-2, the downregulation of miR-200b may promote cell focal adhesion formation and enhance migratory/invasive capabilities [53]. MiR-150 targets NOTCH3, which plays an important role in T-cell differentiation and leukemogenesis, and its reduction reduces T-cell proliferation and survival [54]. In our study, consistent with results in vitrified–warmed embryos, the expression of miR-150-5p was decreased, promoting successful implantation by regulating protein kinase activity and T-cell proliferation. This phenomenon, observed for the first time in embryos, might explain the improved implantation rates in mouse embryos in our study.

In stem cell differentiation, miR-361, secreted by human embryos, may regulate endometrial epithelial cell adhesion, and the downregulation of miR-361 may support implantation [55]. Furthermore, decreased expression of miR-9 inhibits human stem cell proliferation but enhances stem cell invasion [56], which may promote embryo implantation. Our study observed a decrease in miR-103a in embryos, with previous research also noting decreased miR-103a expression in follicular fluid, linked to improved embryonic development and success in IVF and embryo transfer [57]. Lower levels of miR-150-5p in the endometrium, plasma, or plasma exosomes have been identified as biomarkers for implantation [58,59]. MiR-148b, in vitrified–warmed sperm and oocytes, regulates fertilization and early embryonic development through the PTEN gene [60]. The miR-130b and miR-301b clusters are known to be involved in cellular aging and embryonic stem cell differentiation, and their decreased expression can lead to reduced cellular aging and increased cellular synthesis [61,62]. The simultaneous analysis of RNA-Seq and microRNA data confirmed that vitrified embryos might gain advantages in the preimplantation stages by modulating the immune response and stem cell regulation.

This study has several limitations. First, due to the small number of cells in each sample and the rapid changes in gene expression, some mRNAs were present only in the case or control cells. Consequently, we included many genes with significant fold changes in our list. After enrichment of the gene pathway and microRNA gene set, we performed qPCR verification on the critical genes in the pathway and microRNA array verification on the predicted microRNAs. Second, there are differences in the developmental patterns of mouse and human blastocysts, which may affect the generalizability of our findings to human embryos. Lastly, we did not specifically modify the mothers’ diet or hormone status, factors that could influence the interpretation of the results. We used a human cryopreservation kit on mouse blastocysts, and our in vivo study showed an increased implantation success rate for the vitrified mouse blastocysts.

Despite these limitations, our findings provide valuable insights into the molecular mechanisms by which vitrified–warmed embryos respond to cold stress and potential damage. The identification of key microRNAs and mRNA pathways involved in these processes enhances our understanding of the biological and physiological adaptations that support successful implantation and development. Further research is needed to explore these mechanisms in human embryos and to evaluate the potential clinical applications of our findings.

## 4. Materials and Methods

### 4.1. Mice

This study, which aimed to focus on the impact of freezing and thawing on embryos, was conducted under carefully controlled conditions. We selected commonly used, age-appropriate mouse strains to minimize unexpected interference from the maternal aspect. Female C57BL/6JNarl mice aged 6–8 weeks, male C57BL/6JNarl mice aged 10 weeks, and female and male ICR mice aged 8–12 weeks were obtained from the National Laboratory Animal Center in Taipei, Taiwan. These mice were housed in environments with humidity levels between 40% and 60%, a temperature of 22 ± 2 °C, and a photoperiod of 12 h of light and 12 h of darkness. This meticulous control of the environment ensures the reliability of our results. They had free access to food and water.

All animal experimental protocols involving animals underwent a rigorous review and were approved by the Institutional Animal Care and Use Committee of Chung Shan Medical University, Taichung, Taiwan (IACUC approval no. 2341). To minimize the impact of elevated carbon dioxide levels in the blood on the embryonic condition, all experimental mice were euthanized by the cervical dislocation method.

### 4.2. Preparation of Embryos

Superovulation was induced in 6–8-week-old virgin mice of the C57BL/6JNarl strain through intraperitoneal injection of 5IU pregnant mare serum gonadotrophin (Sigma Chemical Co., St. Louis, MO, USA), followed 48 h later by injection with 5 IU of human chorionic gonadotrophin (Serono, Rome, Italy). Each super-ovulated mouse was placed overnight in a cage with a sexually mature male mouse of the same strain [63]. The presence of a copulation plug in the vagina determined whether mating was successful. The mated female mice were euthanized through cervical dislocation 20 h after human chorionic gonadotrophin injection, and zygotes were collected from the oviducts. The cumulus cells of the zygotes were removed by exposure to hyaluronidase (80 IU/mL; Sigma). The zygotes were placed into wells with a fresh human tubal fluid medium. The HTF medium was prepared in-house following the protocol described by Quinn (1995) in “Enhanced Results in Mouse and Human Embryo Culture Using a Modified Human Tubal Fluid Medium Lacking Glucose and Phosphate” [64]. Embryos in the blastocyst stage were obtained by incubating the zygotes in an atmosphere with 5% CO_2_ at 37 °C for three days. Blastocysts were evaluated using the mouse grading system, and grade II and III blastocysts were selected as candidates for embryo transfer [65]. Images of the selected mouse embryos are provided as Supplementary Materials.

### 4.3. Embryo Vitrification and Warming

Significant species differences exist between humans and mice, so generalizations should be made cautiously. However, this study aimed to simulate human embryo vitrification closely. Therefore, we utilized human protocols on mouse embryos. The embryo vitrification process was carried out using a Cryotech Vitrification Kit (Cryotech, Tokyo, Japan), following the manufacturer’s instructions and the protocol outlined by Gutnisky et al. [66]. This kit comprises various solutions and devices designed for vitrifying and warming oocytes and embryos. We meticulously followed the manufacturer’s instructions for the experiments conducted herein.

Briefly, mouse embryos were equilibrated at room temperature for approximately 10 to 15 min in equilibration solution (ES), then transferred to vitrification solution (VS). After repeated aspiration in the VS, embryos and a minimal amount of liquid were individually loaded onto film strips and swiftly immersed in liquid nitrogen. For warming, the strips were directly immersed in the warming solution (TS) at 37 °C for 1 min. Embryos were then incubated for 3 min in diluent solution (DS) and washed twice for 5 min each in washing solution (WS). Post-warming, the embryos were incubated in human tubal fluid for 2 h before embryo transfer.

### 4.4. Embryo Transfer

Five recipient female mice (8–12-week-old, ICR strain) were prepared by mating with vasectomized male mice of the same strain 3 days before embryo transfer. Mating was confirmed by the presence of a vaginal plug the following morning. Thirty vitrified–warmed and thirty fresh blastocyst embryos from four C57BL/6JNarl mice were prepared for embryo transfer. Embryo transfer was performed 2.5 days following mating. Six vitrified–warmed blastocysts were transferred to the right uterine horn in the same recipient. The same number of fresh blastocysts was transferred to the left uterine horn of pseudo-pregnant recipients. The mice were euthanized 4 days after embryo transfer.

### 4.5. RNA-Seq Analysis

Transcriptomic analysis, a crucial component of this study, was performed using RNA sequencing technology to obtain mRNA expression information. For both vitrified–warmed and fresh embryos, 100 blastocysts were collected for each group. The embryos in each group were pooled and then divided into three aliquots for triplicate sequencing. This design resulted in six sample outcomes: three from vitrified–warmed embryos and three from fresh embryos.

Samples of 1 µL were taken to construct a library using the Ovation SoLo RNA-Seq library preparation kit (NuGEN Technologies, Redwood City, CA, USA; #0501-32). Two systems were used to assess the concentration and quality of the mRNA library. The concentration of mRNA was determined using the highly sensitive and accurate fluorescence-based Qubit 2.0 Fluorometer (Thermo Fisher Scientific, Waltham, MA, USA) with the dsDNA High Sensitivity Kit (Thermo Fisher Scientific, Waltham, MA, USA). The Agilent 2100 Bioanalyzer system (Agilent Technologies, Santa Clara, CA, USA) with the DNA 1000 assay assessed the mRNA quality. Sequencing commenced after confirming that the library exhibited good quality (an RNA integrity number, RIN, greater than 7 was considered the standard). Normalized RNA libraries were clustered and sequenced on an Illumina NovaSeq Sequencing System (Illumina, Inc., San Diego, CA, USA) (2 × 150 bp, paired-end). Each sample sequenced yielded at least five million reads.

This approach allowed us to maximize the reliability of transcriptomic data while working with a limited number of embryos. By pooling 100 embryos before division, we ensured sufficient RNA for analysis while minimizing individual variation. The triplicate sequencing of each pooled sample enhanced the statistical significance and reproducibility of our results, enabling a comprehensive understanding of the transcriptomic changes induced by the vitrification process.

### 4.6. Data Preprocessing

The resulting reads underwent quality control, trimming, alignment to the *Mus musculus* GRCm38 reference genome, and calculation of gene expression values using CLC Genomics Workbench v.10 (https://www.qiagenbioinformatics.com/products/clc-genomics-workbench/, accessed on 8 February 2021). The QC method of CLC Genomics Workbench v.10 was based on FastQC v 0.11.5 (http://www.bioinformatics.babraham.ac.uk/projects/fastqc/, accessed on 8 February 2021). This process involved removing low-quality sequences (limit = 0.01, with a quality value (QV) greater than 20) and any ambiguous nucleotides, with no ambiguous nucleotides allowed. Adapter sequences were also removed, using the following adapters: universal and indexed adapter ‘GCTCTTCCGATCT’. The alignment to the *Mus musculus* reference genome required that at least 50% of the reads’ length could be mapped to the reference genome, with at least 80% similarity in the mapped portion.

Gene expression data were normalized using Fragments Per Kilobase per Million (FPKM) [67] and calibrated using the geometric mean of housekeeping genes (including 12 genes: *Actb, B2m*, *Gapdh*, *Hprt*, *Pgk1*, *Ppia*, *Rpl13a*, *Rplp0*, *Sdha*, *Tbp*, *Tfrc*, and *Ywhaz*).

### 4.7. Real-Time RT-PCR

To ensure reproducibility, we repeated the earlier procedures and collected 100 embryos from each group to pool together for real-time RT-PCR experiments. We verified partial NGS gene expression using real-time RT-PCR. mRNA was isolated from individually collected embryos using the Dynabeads mRNA DIRECT Micro Kit (Dynal Biotech ASA, Oslo, Norway) following the manufacturer’s instructions.

RT reactions were performed in 17 μL of diethyl-pyrocarbonate-treated water, 1 μL of RNaseOUT recombinant RNase inhibitor (40 U/μL; Invitrogen, Carlsbad, CA, USA), 1 μL of dNTPs (10 mM each dATP, dGTP, dCTP, and dTTP), 1 μL of Oligo (dT) 20 primer (50 μM; Invitrogen), 4 μL of 5X First-Strand Buffer (Invitrogen), 2 μL of DTT (0.1 M), and 1 μL of SuperScript III Reverse Transcriptase (200 U/μL; Invitrogen). The reaction was conducted at 50 °C for 1 h, then at 70 °C for 15 min, 4 °C for 2 min, and 37 °C for 20 min. The resulting cDNA was used for RT-PCR.

RT-PCR was performed with SYBR green PCR master mix (Applied Biosystems, Foster City, CA, USA) and using an ABI 7300 system (Applied Biosystems). Cycling consisted of an initial holding stage (50 °C for 2 min), 95 °C for 10 min, 40 cycles of 95 °C for 15 s and 60 °C for 1 min, and a dissociation stage (95 °C for 15 s, 60 °C for 1 min, and 95 °C for 15 s). Primers are listed in Supplementary Table S4.

### 4.8. MicroRNA Expression Analysis in Vitrified–Warmed Blastocysts

We conducted a miRCURY experiment to validate the microRNA expression results in vitrified–warmed blastocysts. The miRCURY LNA RT Kit is a PCR-based system specifically designed for microRNA array analysis. A total of 150 vitrified–warmed and 150 fresh embryos were individually collected for microRNA expression analysis. MicroRNA expression levels were assessed using microRNA array analysis as part of the transcriptomic analysis.

Total RNA isolation was performed using TriFastFL (VWR/Peqlab, Erlangen, Germany) following the manufacturer’s instructions. RNA concentration and quality were determined using a NanoDrop spectrophotometer (Thermo Fisher Scientific, Dreieich, Germany), with an absorbance wavelength ratio of A260/280  >  1.8 considered suitable for microRNA analysis. Subsequently, cDNA was synthesized using the miRCURY LNA RT Kit (QIAGEN, Hilden, Germany) according to the standard protocol.

The miRCURY analysis established certain quality standards:

S: CT values were <30, indicating relatively high microRNA expression in fresh and vitrified–warmed blastocysts. 

A: MicroRNA expression was relatively high in both tests, with CT values < 30 in one test and >30 in the other. 

B: MicroRNA expression was relatively low in fresh and vitrified–warmed blastocysts, with CT values > 30 in both tests. 

C: The data exceeded the threshold cycle cutoff in one or both sets. 

We anticipated using data containing at least level B or higher for further analysis. This PCR-based approach allowed for sensitive and specific quantification of microRNA expression in our blastocyst samples.

### 4.9. Functional Annotation and Pathway Enrichment Analysis of Differentially Expressed Genes

DEGs identified from RNA-seq analysis results between the control and treatment groups were employed for functional annotation and biological pathway enrichment to elucidate the biological changes and subsequent clinical implications associated with vitrified–warmed embryos. GO term and KEGG pathway enrichment analyses of DEGs were conducted using ShinyGO v.0.80 [13]. To elucidate further gene expression patterns related to vitrified–warmed embryos from a fundamental biological perspective.

### 4.10. MicroRNA Analysis and Functional Enrichment in Vitrified–Warmed Embryos

MicroRNA regulates various biological and physiological processes, including most events in mammalian reproduction. Understanding the natural function of microRNA in vitrified–warmed embryos can enhance our comprehension of embryo locomotion and implantation. We predicted the microRNA expression differences based on DEGs. At this stage, microRNA with a *p*-value of 0.05 was predicted using the analysis package provided by Gene Set Enrichment Analysis, GSEA v.4.1.0 [14,15]. The annotation gene set utilized the C3 molecular signatures database, comprising genes regulating target genes based on predictions for microRNA and transcription factor binding sites [15,68,69]. Subsequently, we correlated the predicted results with the microRNA analysis from the previous section, establishing a longitudinal link between microRNA regulation and mRNA expression. Finally, we used TAM 2.0 to perform functional enrichment analysis of microRNAs [70], applying a Bonferroni *p*-value of 0.05 and ensuring that the number of microRNAs was at least three.

## 5. Conclusions

The findings of this study enhance our understanding of the impact of vitrification on the implantation and developmental potential of blastocysts in mammalian species. These changes involve regulating stress physiology, promoting self-developmental progress, and preparing for preimplantation. Furthermore, it has been observed that microRNAs also undergo changes, and these altered microRNAs may participate in embryo implantation through the modulation of immune responses or stem cell regulation, as indicated in the existing literature.

By promoting the process of self-development, there is a potential for more robust embryonic development and an increase in the implantation rate. Our findings suggest that the enhanced implantation rate in frozen embryo transfer cycles may be attributed to the modified gene expression profiles of vitrified blastocysts rather than solely relying on a selective, more natural endometrial environment.

## Figures and Tables

**Table 1:** The number of successful implantations of fresh and vitrified–warmed blastocysts. Recipient female mice (8–12-week-old, ICR strain) were prepared by mating with vasectomized male mice of the same strain 4 days before embryo transfer. Mating was confirmed by the presence of a vaginal plug the following morning. The embryos prepared from C57BL/6JNarl mice were cultured in human tubal fluid for 3 days. The blastocyst transfer was performed 2.5 days following mating. Six vitrified–warmed blastocysts were transferred into the right uterine horn of pregnant mice, and an equal number of fresh blastocysts were transferred into the left uterine horn of the same mice. The mice were euthanized 4 days after embryo transfer.

Number of Mice	Fresh Blastocyst	Vitrified–Warmed Blastocyst
No.1	3/6	3/6
No.2	4/6	6/6
No.3	2/6	5/6
No.4	6/6	6/6
No.5	2/6	5/6
total	17/30 (56.7%)	25/30 (83.3%)

**Table 2:** Summary of microRNA biomarker candidates for implantation advantage of vitrified–warmed blastocysts. The mRNA gene expression profile with gene set enrichment analysis (GSEA) was performed to predict miRNA that may regulate embryonic development. In addition, we performed a miRCURY experiment to verify the results of miRNA expression in vitrified–warmed blastocysts.

microRNA	*p*-Value of GSEA ^1^	NES of GSEA ^2^	miRCURY Log2 of Fold Change ^3^
miR-200b-3p	0.010	−1.28	−0.60
miR-361-5p	0.020	−1.36	−0.62
miR-93-3p	0.020	−1.37	−0.62
miR-9-5p	0.016	−1.27	−0.76
miR-30e-3p	0.007	−1.31	−0.79
miR-214-3p	0.009	−1.39	−0.81
miR-148b-3p	0.017	−1.29	−0.86
miR-301b-3p	0.027	−1.25	−1.00
miR-103a-2-5p	0.031	−1.33	−1.18
miR-30d-3p	0.006	−1.30	−1.25
miR-130b-3p	0.021	−1.24	−1.29
miR-150-5p	0.001	−1.54	−1.36

^1^* p*-value < 0.05. ^2^ NES, normalized enrichment score by GSEA v4.1.0. GSEA normalizes the enrichment scores to produce a normalized enrichment score, which can be used to compare the enrichment of individual enrichments. The plus or minus sign can indicate the directionality of gene expression in the enrichment, but the NES does not represent the intensity of microRNA expression. ^3^ Experimental log-2-fold changes are more than 0.58 or less than −0.58 (this means fold changes are more than 1.5 or less than 0.67) between vitrified–warmed and fresh blastocysts.

**Table 3:** Function enrichment analysis was conducted on miRNA derived from vitrified–warmed blastocysts. The various functions of the miRNA impacted the regulation of embryonic implantation and development. This function list can infer and assess how the vitrified–warmed process influences embryos, leading to a higher successful implantation rate in vitrified–warmed blastocysts.

Function	Bonferroni *p*-Value ^#^	microRNA ^$^
Immune Response	9.30 × 10^−4^	mir-9, mir-103a, mir-150, mir-93, mir-200b
Embryonic Stem Cell Differentiation	9.30 × 10^−4^	mir-9, mir-103a, mir-130b
Nephrotoxicity	2.40 × 10^−3^	mir-30d, mir-130b, mir-30e, mir-200b
Regulation of Stem Cell	6.54 × 10^−3^	mir-9, mir-200b, mir-214, mir-93
Cell Proliferation	7.04 × 10^−3^	mir-9, mir-200b, mir-93, mir-150
Cell Cycle	8.73 × 10^−3^	mir-9, mir-103a, mir-200b, mir-150
Aging	3.35 × 10^−2^	mir-9, mir-30d, mir-30e
Inflammation	4.92 × 10^−2^	mir-9, mir-150, mir-93, mir-130b

^#^ Bonferroni *p*-value < 0.05. ^$^ The number of microRNAs is at least three.

## Data Availability

The data for this study are available and can be accessed via the National Center for Biotechnology Information (NCBI) Gene Expression Omnibus (GEO) database under the accession number GSE268758, which can be found at https://www.ncbi.nlm.nih.gov/geo/query/acc.cgi?&acc=GSE268758.

## Supplementary Materials

The following supporting information can be downloaded at: https://www.mdpi.com/article/10.3390/ijms25168658/s1.
