# Supplementary material for: Transcriptomic Analysis of Vitrified–Warmed vs. Fresh Mouse Blastocysts: Cryo-Induced Physiological Mechanisms and Implantation Impact
Source: Int J Mol Sci. 2024 Aug 8;25(16):8658. doi: 10.3390/ijms25168658 (PMC11354596; doi:10.3390/ijms25168658)
Supplement: Supplementary file 1 [file ijms-25-08658-s001.zip › Supplementary Table S1.pdf]

Supplementary Table S1 presents the results of the experiments. The "Fresh-1" group refers to the direct implantation of mouse embryos, while the "Cryo-2" group denotes the implantation of mouse embryos after vitrification and thawing. The p-value for Cryo-2 vs. Fresh-1 indicates the level of statistical significance between the two groups, with a cutoff value of 0.05. Owing to the limited amount of mRNA in each sample and the embryos' rapid developmental stage, several gene expressions were found to be exclusive to specific samples. Consequently, we set the criterion for FPKM Mean of six samples > 0.5. The mean values of the Fresh-1 and Cryo-2 groups calculated the Normalized Fold Change LOG2(Cryo-2/Fresh-1). If one of the values was zero, it was replaced with 0.000001 before calculating the fold change.Table sorting is indicated by Normalized Fold Change LOG2(Cryo-2 / Fresh-1).

Official Gene Symbol	Entrez Gene ID	Gene description	Normalized Fold Change LOG2(Cryo-2 / Fresh-1)	FPKM Mean of six samples	p-value of Cryo-2 vs Fresh-1
Moap1	64113	modulator of apoptosis 1	21.477	1.27	4.65E-17
Syce3	75459	synaptonemal complex central element protein 3	21.147	1.01	1.75E-07
LOC102642720	102642720	-	20.958	0.89	2.89E-09
Myl7	17898	myosin, light polypeptide 7, regulatory	20.944	0.88	3.25E-07
LOC100044633	100044633	-	20.755	0.77	4.99E-06
Ppp1r2-ps3	546723	protein phosphatase 1, regulatory (inhibitor) subunit 2, pseudogene 3	20.713	0.75	6.98E-08
Gstm6	14867	glutathione S-transferase, mu 6	20.629	0.71	3.30E-10
LOC102638962	102638962	-	20.619	0.70	3.44E-06
Fbxw14	50757	F-box and WD-40 domain protein 14	20.595	0.69	3.46E-11
LOC105245424	105245424	-	20.435	0.62	4.53E-07
Olfr134	258829	olfactory receptor 134	20.273	0.55	3.06E-07
Fbxw24	382106	F-box and WD-40 domain protein 24	6.831	0.60	1.64E-07
LOC105245381	105245381	-	6.622	0.69	7.10E-07
Slc51b	330962	solute carrier family 51, beta subunit	6.405	2.78	3.98E-09
Cml1	66116	camello-like 1	6.189	0.57	2.44E-06
Timp1	21857	tissue inhibitor of metalloproteinase 1	6.083	0.66	4.37E-06
Oxld1	66431	oxidoreductase like domain containing 1	6.075	0.69	4.60E-06
Rnu11	353373	U11 small nuclear RNA	5.940	4.51	1.89E-05
Zfp352	236537	zinc finger protein 352	5.818	0.61	2.00E-10
Camk2n2	73047	calcium/calmodulin-dependent protein kinase II inhibitor 2	5.770	1.11	1.33E-10
Rps24-ps2	383815	ribosomal protein S24, pseudogene 2	5.752	0.88	4.70E-05
Tex12	66654	testis expressed gene 12	5.711	1.47	3.53E-07
Fbxo2	230904	F-box protein 2	5.668	0.65	3.88E-07
Rbm14-rbm4	102902673	Rbm14-Rbm4 readthrough	5.537	1.42	1.42E-03
Lmo2	16909	LIM domain only 2	5.529	0.98	3.97E-15
Rsc1a1	69994	regulatory solute carrier protein, family 1, member 1	5.504	4.40	9.92E-03
BC021614	225884	cDNA sequence BC021614	5.415	1.46	9.15E-12
Apoc2	11813	apolipoprotein C-II	5.105	1.14	1.47E-07
Zscan4f	665902	zinc finger and SCAN domain containing 4F	5.092	2.98	1.49E-43
Rpl21-ps5	545172	ribosomal protein L21, pseudogene 5	5.066	0.53	7.32E-04
Snx7	76561	sorting nexin 7	5.031	0.64	4.85E-12
Mnd1	76915	meiotic nuclear divisions 1 homolog (S. cerevisiae)	4.999	0.51	1.51E-09
LOC105244671	105244671	-	4.978	4.86	4.86E-61
LOC102632821	102632821	-	4.945	0.76	1.93E-15
Tctex1d2	66061	Tctex1 domain containing 2	4.847	0.60	4.89E-05
Ptgds	19215	prostaglandin D2 synthase (brain)	4.702	0.76	1.70E-06
Phf7	71838	PHD finger protein 7	4.657	0.60	2.22E-11
Asb3	65257	ankyrin repeat and SOCS box-containing 3	4.653	0.86	7.99E-25
LOC105242648	105242648	-	4.645	0.65	1.26E-11
Abhd8	64296	abhydrolase domain containing 8	4.612	0.96	2.53E-16
Nradd	67169	neurotrophin receptor associated death domain	4.503	0.93	2.79E-10
LOC102638884	102638884	-	4.443	0.89	4.55E-46

Official Gene Symbol	Entrez Gene ID	Gene description	Normalized Fold Change LOG2(Cryo-2 / Fresh-1)	FPKM Mean of six samples	p-value of Cryo-2 vs Fresh-1
Rpl30-ps11	628441	ribosomal protein L30, pseudogene 11	4.271	1.15	4.06E-05
Dand5	23863	DAN domain family, member 5	4.260	0.62	3.12E-09
B3gnt7	227327	UDP-GlcNAc:betaGal beta-1,3-N-acetylglucosaminyltransferase 7	4.216	0.57	2.10E-12
Chrnb1	11443	cholinergic receptor, nicotinic, beta polypeptide 1 (muscle)	4.168	0.67	1.34E-02
H2-Ke6	14979	H2-K region expressed gene 6	4.081	2.29	2.48E-18
Tmed6	66269	transmembrane emp24 protein transport domain containing 6	4.027	0.67	1.55E-05
Tmem106c	380967	transmembrane protein 106C	3.969	0.67	2.88E-09
Ccl9	20308	chemokine (C-C motif) ligand 9	3.942	0.62	1.38E-15
Npy	109648	neuropeptide Y	3.936	0.51	4.12E-03
Efna1	13636	ephrin A1	3.914	0.56	2.10E-08
Xrcc3	74335	X-ray repair complementing defective repair in Chinese hamster cells 3	3.833	1.19	3.38E-27
Dusp9	75590	dual specificity phosphatase 9	3.715	0.65	3.20E-05
LOC105246824	105246824	-	3.657	2.18	3.55E-14
Mthfsl	100039707	5, 10-methenyltetrahydrofolate synthetase-like	3.504	1.22	2.03E-18
LOC102635075	102635075	-	3.485	0.50	7.09E-04
BC024978	414069	cDNA sequence BC024978	3.475	0.68	4.97E-19
Pxt1	69307	peroxisomal, testis specific 1	3.468	0.50	1.89E-04
Tarm1	245126	T cell-interacting, activating receptor on myeloid cells 1	3.454	0.80	1.44E-10
LOC102639700	102639700	-	3.421	1.65	4.85E-18
Slc30a1	22782	solute carrier family 30 (zinc transporter), member 1	3.402	8.21	4.96E-269
Mt1	17748	metallothionein 1	3.398	449.79	1.27E-111
Srd5a3	57357	steroid 5 alpha-reductase 3	3.374	1.32	9.24E-20
Lst1	16988	leukocyte specific transcript 1	3.356	1.33	7.62E-05
Cyp2f2	13107	cytochrome P450, family 2, subfamily f, polypeptide 2	3.355	0.59	5.43E-08
Slc3a1	20532	solute carrier family 3, member 1	3.340	1.22	1.67E-19
Lrrc51	69358	leucine rich repeat containing 51	3.332	0.61	4.81E-02
Slc30a2	230810	solute carrier family 30 (zinc transporter), member 2	3.318	1.51	2.38E-39
Rnase4	58809	ribonuclease, RNase A family 4	3.259	0.75	6.63E-12
Vim	22352	vimentin	3.203	0.51	2.07E-07
Cluap1	76779	clusterin associated protein 1	3.195	1.23	1.95E-33
Pgc	109820	progastricsin (pepsinogen C)	3.194	0.54	6.91E-06
Hba-a1	15122	hemoglobin alpha, adult chain 1	3.179	3.23	1.36E-13
Acot1	26897	acyl-CoA thioesterase 1	3.154	0.80	3.33E-09
Lmo4	16911	LIM domain only 4	3.150	1.59	3.51E-22
Mt2	17750	metallothionein 2	3.150	235.30	0.00E+00
LOC102638361	102638361	-	3.144	0.60	1.14E-05
Obp2a	227627	odorant binding protein 2A	3.137	0.52	2.66E-06
Pcna-ps2	18540	proliferating cell nuclear antigen pseudogene 2	3.132	1.48	2.11E-13
Nrp	654309	neural regeneration protein	3.102	0.94	1.63E-08
Myl4	17896	myosin, light polypeptide 4	3.098	0.90	3.41E-09
Ggnbp2os	102635561	-	3.094	0.77	1.30E-04
Spink2	69982	serine peptidase inhibitor, Kazal type 2	3.089	0.75	2.23E-05
Tmem40	94346	transmembrane protein 40	3.076	0.59	7.19E-10
LOC105247048	105247048	-	3.052	0.99	1.42E-07
Bex6	328660	brain expressed gene 6	3.004	0.83	3.57E-06

Official Gene Symbol	Entrez Gene ID	Gene description	Normalized Fold Change LOG2(Cryo-2 / Fresh-1)	FPKM Mean of six samples	p-value of Cryo-2 vs Fresh-1
Fam120aos	68128	family with sequence similarity 120A, opposite strand	2.995	2.42	4.98E-29
Bphl	68021	biphenyl hydrolase-like (serine hydrolase, breast epithelial mucin-associated antigen)	2.988	0.65	1.27E-06
Zfp688	69234	zinc finger protein 688	2.982	0.64	1.68E-15
LOC102640575	102640575	-	2.955	2.19	4.06E-06
Ak3l2-ps	635960	adenylate kinase 3-like 2, pseudogene	2.918	0.51	2.21E-05
Serf1	20365	small EDRK-rich factor 1	2.918	1.65	4.18E-07
Syce1	74075	synaptonemal complex central element protein 1	2.878	1.14	1.05E-11
Acn9	71238	ACN9 homolog (S. cerevisiae)	2.866	2.73	4.53E-10
B4gat1	108902	UDP-GlcNAc:betaGal beta-1,3-N-acetylglucosaminyltransferase 1	2.853	0.79	1.58E-09
Prr23a	623166	proline rich 23A	2.852	0.50	4.14E-04
Spats2l	67198	spermatogenesis associated, serine-rich 2-like	2.841	0.66	6.30E-20
Il17ra	16172	interleukin 17 receptor A	2.841	0.73	1.43E-17
Rad51c	114714	RAD51 homolog C	2.833	1.25	2.87E-25
Sp6	83395	trans-acting transcription factor 6	2.816	0.50	1.31E-15
Slc25a35	71998	solute carrier family 25, member 35	2.809	1.33	6.54E-16
Tmem129	68366	transmembrane protein 129	2.796	0.99	2.24E-17
Tcf19	106795	transcription factor 19	2.789	0.78	2.44E-09
Zscan4c	245109	zinc finger and SCAN domain containing 4C	2.778	0.58	1.36E-08
Armc7	276905	armadillo repeat containing 7	2.764	1.47	1.20E-28
Rap2a	76108	RAS related protein 2a	2.732	1.23	2.75E-33
Pcbd2	72562	pterin 4 alpha carbinolamine dehydratase/dimerization cofactor of hepatocyte nuclear factor 1 alpha (TCF1) 2	2.692	1.47	1.13E-09
Foxi3	232077	forkhead box I3	2.684	0.67	1.44E-05
Khdrbs3	13992	KH domain containing, RNA binding, signal transduction associated 3	2.661	0.72	2.13E-08
LOC105246186	105246186	-	2.655	1.53	9.32E-25
Grcc10	14790	gene rich cluster, C10 gene	2.649	1.67	2.91E-06
LOC101056074	101056074	-	2.647	0.65	1.40E-11
LOC105242433	105242433	-	2.590	0.94	9.10E-05
Rab2b	76338	RAB2B, member RAS oncogene family	2.586	1.34	5.40E-23
Dlg5	71228	discs, large homolog 5 (Drosophila)	2.580	0.51	4.91E-08
Smarca5-ps	545700	SWI/SNF related, matrix associated, actin depenent ragulator of chromatin, subfamily a, member 5, pseudogene	2.574	0.67	1.67E-15
Tceal8	66684	transcription elongation factor A (SII)-like 8	2.548	0.85	4.58E-12
Wbscr17	212996	Williams-Beuren syndrome chromosome region 17 homolog (human)	2.544	0.57	1.49E-16
Mterf2	74238	mitochondrial transcription termination factor 2	2.541	1.00	4.31E-14
Cbr2	12409	carbonyl reductase 2	2.537	1.76	3.86E-10
Myh6	17888	myosin, heavy polypeptide 6, cardiac muscle, alpha	2.529	0.77	3.00E-25
Ppp1r14d	72112	protein phosphatase 1, regulatory (inhibitor) subunit 14D	2.527	1.20	4.61E-08
Wfdc12	192200	WAP four-disulfide core domain 12	2.520	1.24	9.13E-06
Tff3	21786	trefoil factor 3, intestinal	2.508	1.09	1.77E-03
LOC105244171	105244171	-	2.492	0.66	3.62E-27
Fam221a	231946	family with sequence similarity 221, member A	2.477	0.67	1.16E-10
Mylip	218203	myosin regulatory light chain interacting protein	2.459	1.02	5.45E-16
Npm2	328440	nucleophosmin/nucleoplasmin 2	2.445	1.55	5.07E-44

Official Gene Symbol	Entrez Gene ID	Gene description	Normalized Fold Change LOG2(Cryo-2 / Fresh-1)	FPKM Mean of six samples	p-value of Cryo-2 vs Fresh-1
Rpsa-ps10	100040633	ribosomal protein SA, pseudogene 10	2.440	5.22	1.83E-30
Fam229b	66337	family with sequence similarity 229, member B	2.439	0.60	2.42E-03
Ripk1	19766	receptor (TNFRSF)-interacting serine-threonine kinase 1	2.439	0.87	1.17E-30
Gadd45b	17873	growth arrest and DNA-damage-inducible 45 beta	2.430	1.16	2.25E-08
Pigv	230801	phosphatidylinositol glycan anchor biosynthesis, class V	2.416	19.29	4.96E-250
Acy3	71670	aspartoacylase (aminoacylase) 3	2.411	0.66	5.84E-05
Alkbh7	66400	alkB, alkylation repair homolog 7 (E. coli)	2.403	2.35	6.07E-11
BC037034	231807	cDNA sequence BC037034	2.369	0.62	6.61E-10
Hormad2	75828	HORMA domain containing 2	2.365	0.52	6.19E-12
Rasl10a	75668	RAS-like, family 10, member A	2.330	3.18	1.96E-20
Zfp689	71131	zinc finger protein 689	2.328	0.53	5.41E-09
Ninj1	18081	ninjurin 1	2.309	3.51	1.62E-19
Cd68	12514	CD68 antigen	2.299	1.27	3.46E-08
Dapk2	13143	death-associated protein kinase 2	2.294	0.51	5.11E-05
Pmel	20431	premelanosome protein	2.264	0.67	3.36E-06
Kdelr3	105785	KDEL (Lys-Asp-Glu-Leu) endoplasmic reticulum protein retention receptor 3	2.263	1.89	4.73E-17
LOC105244937	105244937	-	2.262	6.22	5.49E-13
Tsacc	76927	TSSK6 activating co-chaperone	2.246	1.59	1.05E-05
Tango2	27883	transport and golgi organization 2	2.243	1.06	2.03E-12
LOC105246114	105246114	-	2.240	0.63	2.82E-15
Spata7	104871	spermatogenesis associated 7	2.237	2.56	4.59E-27
Zbtb48	100090	zinc finger and BTB domain containing 48	2.231	1.02	2.04E-13
Fv1	14349	Friend virus susceptibility 1	2.226	0.55	8.33E-04
Srrd	70118	SRR1 domain containing	2.224	2.85	5.45E-16
Tmem53	68777	transmembrane protein 53	2.221	0.62	3.86E-04
Pnp2	667034	purine-nucleoside phosphorylase 2	2.199	0.68	1.25E-04
Tmco4	77056	transmembrane and coiled-coil domains 4	2.199	0.88	8.58E-20
Cklf	75458	chemokine-like factor	2.193	1.66	2.01E-17
Ksr1	16706	kinase suppressor of ras 1	2.169	0.99	4.18E-34
Plscr3	70310	phospholipid scramblase 3	2.160	0.59	2.36E-07
Fgf4	14175	fibroblast growth factor 4	2.159	1.20	2.03E-17
Micu3	78506	mitochondrial calcium uptake family, member 3	2.148	0.90	2.95E-14
Vamp2	22318	vesicle-associated membrane protein 2	2.148	0.77	3.27E-08
Dusp5	240672	dual specificity phosphatase 5	2.128	1.34	5.47E-07
Fam53a	74504	family with sequence similarity 53, member A	2.108	2.04	1.21E-19
Ubl4	27643	ubiquitin-like 4	2.108	4.82	2.23E-39
Arid3a	13496	AT rich interactive domain 3A (BRIGHT-like)	2.091	0.89	1.17E-26
Vmn1r56	81015	vomeronasal 1 receptor 56	2.072	0.57	4.00E-05
Mxd3	17121	Max dimerization protein 3	2.069	1.01	4.29E-06
Ddt	13202	D-dopachrome tautomerase	2.061	5.51	2.02E-14
Tmem233	545798	transmembrane protein 233	2.046	2.56	2.52E-08
Rpl31-ps11	668409	ribosomal protein L31, pseudogene 11	2.045	1.27	3.36E-03
BC037704	100502982	cDNA sequence BC037704	2.041	0.72	1.81E-06
Miox	56727	myo-inositol oxygenase	2.034	1.37	1.81E-06
Sct	20287	secretin	2.032	0.87	1.19E-02
Tmem185b	226351	transmembrane protein 185B	2.025	1.79	1.17E-19
Nudt11	58242	nudix (nucleoside diphosphate linked moiety X)-type motif 11	1.994	0.94	1.19E-09
Smad1	17125	SMAD family member 1	1.986	1.11	2.31E-14

Official Gene Symbol	Entrez Gene ID	Gene description	Normalized Fold Change LOG2(Cryo-2 / Fresh-1)	FPKM Mean of six samples	p-value of Cryo-2 vs Fresh-1
Dusp1	19252	dual specificity phosphatase 1	1.985	1.29	6.86E-10
B3glct	381694	beta-3-glucosyltransferase	1.985	0.97	4.24E-24
Cyba	13057	cytochrome b-245, alpha polypeptide	1.974	0.73	8.61E-03
Pot1b	72836	protection of telomeres 1B	1.967	0.76	6.37E-11
Pxmp4	59038	peroxisomal membrane protein 4	1.960	1.56	1.64E-09
Fam228b	207921	family with sequence similarity 228, member B	1.959	0.52	4.29E-10
Cst7	13011	cystatin F (leukocystatin)	1.956	0.73	1.80E-03
Tesk1	21754	testis specific protein kinase 1	1.936	4.27	1.05E-55
Rps12-ps10	670832	-	1.933	3.32	3.42E-07
D1Ertd622e	52392	DNA segment, Chr 1, ERATO Doi 622, expressed	1.932	0.52	3.25E-07
Bloc1s3	232946	biogenesis of lysosomal organelles complex-1, subunit 3	1.927	1.76	6.38E-14
Csf2ra	12982	colony stimulating factor 2 receptor, alpha, low-affinity (granulocyte-macrophage)	1.918	0.96	4.02E-08
Tbcd	108903	tubulin-specific chaperone d	1.911	1.21	7.08E-22
Slc2a7	435818	solute carrier family 2 (facilitated glucose transporter), member 7	1.910	0.62	5.79E-06
Mipol1	73490	mirror-image polydactyly gene 1 homolog (human)	1.907	2.25	9.29E-12
Abracl	73112	ABRA C-terminal like	1.899	2.17	3.82E-08
Gsta1	14857	glutathione S-transferase, alpha 1 (Ya)	1.898	0.80	4.44E-03
Gdpd5	233552	glycerophosphodiester phosphodiesterase domain containing 5	1.896	0.75	6.84E-12
Vstm5	69137	V-set and transmembrane domain containing 5	1.885	0.65	1.07E-06
LOC105246638	105246638	-	1.870	3.23	3.12E-06
Slc16a6	104681	solute carrier family 16 (monocarboxylic acid transporters), member 6	1.859	1.82	3.00E-32
Fam210b	67017	family with sequence similarity 210, member B	1.858	0.88	1.98E-11
Rpl38-ps1	625646	ribosomal protein L38, pseudogene 1	1.840	2.03	1.02E-02
Mybl1	17864	myeloblastosis oncogene-like 1	1.832	0.86	2.89E-19
Necab2	117148	N-terminal EF-hand calcium binding protein 2	1.831	1.08	6.13E-08
Nrm	106582	nurim (nuclear envelope membrane protein)	1.830	2.25	2.72E-12
Zfp42-ps1	22703	zinc finger protein 48, pseudogene 1	1.829	0.72	2.30E-04
Hnf1a	21405	HNF1 homeobox A	1.823	0.79	1.07E-08
Zscan22	232878	zinc finger and SCAN domain containing 22	1.823	0.70	8.05E-08
Slc7a7	20540	solute carrier family 7 (cationic amino acid transporter, y+ system), member 7	1.818	0.53	4.73E-06
Smyd4	319822	SET and MYND domain containing 4	1.814	0.67	1.67E-08
Frs3	107971	fibroblast growth factor receptor substrate 3	1.807	0.53	5.51E-06
Proca1	216974	protein interacting with cyclin A1	1.800	1.03	1.79E-08
Nmrk1	225994	nicotinamide riboside kinase 1	1.799	3.57	6.65E-25
Kctd15	233107	potassium channel tetramerisation domain containing 15	1.790	0.84	6.26E-12
Gins2	272551	GINS complex subunit 2 (Psf2 homolog)	1.782	2.79	3.51E-10
Mob3c	100465	MOB kinase activator 3C	1.774	0.55	3.59E-08
Ppa2	74776	pyrophosphatase (inorganic) 2	1.773	4.16	1.81E-44
Cdo1	12583	cysteine dioxygenase 1, cytosolic	1.754	0.66	8.97E-04
LOC105244535	105244535	-	1.754	0.56	7.49E-12
Hmha1	70719	histocompatibility (minor) HA-1	1.753	0.76	3.44E-11
Col26a1	140709	collagen, type XXVI, alpha 1	1.753	0.63	5.68E-08
Pxmp2	19301	peroxisomal membrane protein 2	1.740	1.41	4.82E-05
Pigp	56176	phosphatidylinositol glycan anchor biosynthesis, class P	1.740	3.14	6.57E-15

Official Gene Symbol	Entrez Gene ID	Gene description	Normalized Fold Change LOG2(Cryo-2 / Fresh-1)	FPKM Mean of six samples	p-value of Cryo-2 vs Fresh-1
Fbxo27	233040	F-box protein 27	1.739	0.51	5.58E-05
Chga	12652	chromogranin A	1.738	1.93	7.80E-13
Nfkbia	18035	nuclear factor of kappa light polypeptide gene enhancer in B cells inhibitor, alpha	1.737	1.13	1.52E-06
Capn6	12338	calpain 6	1.735	1.28	3.34E-14
Mir22hg	100042498	Mir22 host gene (non-protein coding)	1.733	1.29	1.07E-20
Cirbp	12696	cold inducible RNA binding protein	1.731	2.80	1.87E-14
Gale	74246	galactose-4-epimerase, UDP	1.731	1.05	2.42E-06
Znrd1as	76416	Znrd1 antisense	1.714	4.60	2.01E-24
Themis3	74556	thymocyte selection associated family member 3	1.713	0.61	3.22E-04
Sac3d1	66406	SAC3 domain containing 1	1.707	5.68	2.78E-22
Shisa7	232813	shisa family member 7	1.692	5.89	4.98E-88
Sdccag8	76816	serologically defined colon cancer antigen 8	1.690	1.24	1.87E-13
Bc1	100568459	brain cytoplasmic RNA 1	1.683	41.80	1.07E-18
Ldlrad2	435811	low density lipoprotein receptor A domain containing 2	1.664	0.50	5.29E-05
Pla2g7	27226	phospholipase A2, group VII (platelet-activating factor acetylhydrolase, plasma)	1.653	1.62	6.44E-11
Zfp2	22678	zinc finger protein 2	1.648	1.24	3.43E-11
Echdc3	67856	enoyl Coenzyme A hydratase domain containing 3	1.632	0.51	1.74E-03
Dos	100503659	downstream of Stk11	1.629	0.66	1.79E-09
Jun	16476	jun proto-oncogene	1.628	4.51	1.41E-37
Stk19	54402	serine/threonine kinase 19	1.621	2.27	1.62E-08
Zfp605	675812	zinc finger protein 605	1.620	1.58	1.88E-19
Usp49	224836	ubiquitin specific peptidase 49	1.618	0.86	2.18E-14
Akr1e1	56043	aldo-keto reductase family 1, member E1	1.616	3.98	3.81E-20
Ppp1r3e	105651	protein phosphatase 1, regulatory (inhibitor) subunit 3E	1.612	0.70	6.79E-04
LOC105244295	105244295	-	1.611	0.59	2.78E-28
Rpl36a-ps1	100043787	ribosomal protein L36A, pseudogene 1	1.603	2.76	1.44E-04
Zfp273	212569	zinc finger protein 273	1.602	3.27	3.77E-21
Klk7	23993	kallikrein related-peptidase 7 (chymotryptic, stratum corneum)	1.601	1.68	1.32E-08
Mrps16	66242	mitochondrial ribosomal protein S16	1.589	6.71	1.42E-11
Tspan4	64540	tetraspanin 4	1.588	1.89	1.02E-09
Ogfod3	66179	2-oxoglutarate and iron-dependent oxygenase domain containing 3	1.587	2.10	7.38E-08
Gstm2	14863	glutathione S-transferase, mu 2	1.585	0.70	1.02E-02
Rnu3a	19850	U3A small nuclear RNA	1.577	94.40	3.15E-23
Rab40b	217371	Rab40B, member RAS oncogene family	1.576	0.92	4.58E-04
Gipc2	54120	GIPC PDZ domain containing family, member 2	1.575	2.29	1.39E-08
Hist1h2ai	319191	histone cluster 1, H2ai	1.571	28.24	3.16E-30
Lxn	17035	latexin	1.565	1.16	6.50E-04
Ulbp1	77777	UL16 binding protein 1	1.564	0.98	1.23E-05
Trp53rk	76367	transformation related protein 53 regulating kinase	1.564	0.66	2.23E-09
Ift43	76411	intraflagellar transport 43	1.563	1.25	2.47E-16
Mocs1	56738	molybdenum cofactor synthesis 1	1.563	1.05	5.92E-08
Zfp493	72958	zinc finger protein 493	1.560	0.81	5.44E-07
Fhl2	14200	four and a half LIM domains 2	1.559	2.49	1.07E-11
Cldn9	56863	claudin 9	1.552	0.55	6.40E-03
Acp6	66659	acid phosphatase 6, lysophosphatidic	1.546	2.06	1.16E-09
Tmem27	57394	transmembrane protein 27	1.542	2.31	1.03E-08

Official Gene Symbol	Entrez Gene ID	Gene description	Normalized Fold Change LOG2(Cryo-2 / Fresh-1)	FPKM Mean of six samples	p-value of Cryo-2 vs Fresh-1
Hsbp1l1	66255	heat shock factor binding protein 1-like 1	1.538	1.60	4.40E-10
Zfp691	195522	zinc finger protein 691	1.536	1.35	9.87E-09
Repin1	58887	replication initiator 1	1.533	1.14	4.03E-09
Slc50a1	19729	solute carrier family 50 (sugar transporter), member 1	1.526	0.77	1.11E-02
Gdf11	14561	growth differentiation factor 11	1.524	0.77	8.43E-04
Cyp4a14	13119	cytochrome P450, family 4, subfamily a, polypeptide 14	1.524	0.85	1.61E-06
Icosl	50723	icos ligand	1.523	2.07	3.66E-15
Mov10	17454	Moloney leukemia virus 10	1.522	2.66	6.97E-25
Fam57a	116972	family with sequence similarity 57, member A	1.522	0.78	4.30E-05
Ccdc61	232933	coiled-coil domain containing 61	1.520	1.27	6.21E-07
Gdpgp1	269952	GDP-D-glucose phosphorylase 1	1.518	1.07	1.65E-10
Uhrf2	109113	ubiquitin-like, containing PHD and RING finger domains 2	1.517	0.98	2.21E-09
Gpr123	52389	G protein-coupled receptor 123	1.514	6.71	3.66E-55
En2	13799	engrailed 2	1.513	11.67	5.96E-55
Mfsd7b	226844	major facilitator superfamily domain containing 7B	1.511	0.60	7.41E-07
Ino80b	70020	INO80 complex subunit B	1.504	3.44	2.79E-09
Ccdc51	66658	coiled-coil domain containing 51	1.496	0.64	1.67E-05
Rpl5-ps2	668936	ribisomal protein L5, pseudogene 2	1.496	1.76	5.17E-05
Traf3ip2	103213	TRAF3 interacting protein 2	1.495	1.44	3.30E-11
Bbs10	71769	Bardet-Biedl syndrome 10 (human)	1.489	0.87	9.65E-07
Swsap1	66962	SWIM type zinc finger 7 associated protein 1	1.489	1.54	2.73E-07
Zfp933	242747	zinc finger protein 933	1.485	1.42	2.55E-15
Dpagt1	13478	dolichyl-phosphate (UDP-N-acetylglucosamine) acetylglucosaminephosphotransferase 1 (GlcNAc-1-P transferase)	1.483	2.09	1.99E-13
Slc17a5	235504	solute carrier family 17 (anion/sugar transporter), member 5	1.481	2.23	1.09E-19
Mypop	232934	Myb-related transcription factor, partner of profilin	1.480	1.12	3.87E-09
Ovca2	246257	candidate tumor suppressor in ovarian cancer 2	1.480	2.49	2.83E-13
Rassf3	192678	Ras association (RalGDS/AF-6) domain family member 3	1.474	2.24	2.29E-21
Rps4x-ps	667617	Rps4x retrotransposed pseudogene	1.471	1.48	5.24E-04
Saysd1	67509	SAYSVFN motif domain containing 1	1.470	0.65	3.31E-04
Txnrd2	26462	thioredoxin reductase 2	1.466	1.66	4.70E-08
Shb	230126	src homology 2 domain-containing transforming protein B	1.466	0.81	5.10E-06
Pstpip2	19201	proline-serine-threonine phosphatase-interacting protein 2	1.463	1.98	1.59E-11
Tmem107	66910	transmembrane protein 107	1.462	1.81	3.19E-06
Rps10-ps1	668457	ribosomal protein S10, pseudogene 1	1.462	1.15	2.64E-02
Agrp	11604	agouti related protein	1.460	1.20	3.27E-03
LOC105246068	105246068	-	1.457	0.82	1.03E-19
Zhx3	320799	zinc fingers and homeoboxes 3	1.456	0.84	3.04E-08
AA414768	245350	expressed sequence AA414768	1.455	1.83	7.59E-08
Tmem60	212090	transmembrane protein 60	1.455	8.48	6.57E-15
Arf2	11841	ADP-ribosylation factor 2	1.452	3.77	4.53E-23
Vps28	66914	vacuolar protein sorting 28 (yeast)	1.451	16.04	1.86E-30
Adad2	75773	adenosine deaminase domain containing 2	1.451	1.09	6.34E-06
LOC102638448	102638448	-	1.449	44.68	3.67E-51

Official Gene Symbol	Entrez Gene ID	Gene description	Normalized Fold Change LOG2(Cryo-2 / Fresh-1)	FPKM Mean of six samples	p-value of Cryo-2 vs Fresh-1
Tmem18	211986	transmembrane protein 18	1.443	2.58	2.11E-17
Trmt61a	328162	tRNA methyltransferase 61A	1.440	0.68	1.11E-04
Lrrc4	192198	leucine rich repeat containing 4	1.424	0.57	4.34E-04
Zswim7	69747	zinc finger SWIM-type containing 7	1.423	7.10	1.11E-10
Cenpw	66311	centromere protein W	1.421	11.50	7.98E-38
Stx1a	20907	syntaxin 1A (brain)	1.419	0.90	1.24E-05
Slc1a5	20514	solute carrier family 1 (neutral amino acid transporter), member 5	1.411	1.79	6.31E-12
Hbb-bs	100503605	hemoglobin, beta adult s chain	1.407	3.58	6.37E-06
Zfp142	77264	zinc finger protein 142	1.406	1.81	4.16E-36
LOC102640977	102640977	-	1.398	0.99	6.32E-03
Sfxn5	94282	sideroflexin 5	1.395	0.66	5.89E-06
Lamtor2	83409	late endosomal/lysosomal adaptor, MAPK and MTOR activator 2	1.391	15.94	1.61E-20
Prr5	109270	proline rich 5 (renal)	1.391	1.31	3.69E-13
Jund	16478	jun D proto-oncogene	1.388	5.67	1.58E-35
Pip5k1c	18717	phosphatidylinositol-4-phosphate 5-kinase, type 1 gamma	1.386	2.63	1.20E-21
Procr	19124	protein C receptor, endothelial	1.383	1.01	9.76E-05
Ndufa13	67184	NADH dehydrogenase (ubiquinone) 1 alpha subcomplex, 13	1.380	1.94	1.08E-10
Cdkn1c	12577	cyclin-dependent kinase inhibitor 1C (P57)	1.379	1.42	2.75E-06
Sco2	100126824	SCO cytochrome oxidase deficient homolog 2 (yeast)	1.379	1.92	2.44E-05
Xndc1	102443350	Xrcc1 N-terminal domain containing 1	1.378	1.18	6.19E-09
Ccdc160	434778	coiled-coil domain containing 160	1.376	2.93	9.24E-18
Smpd2	20598	sphingomyelin phosphodiesterase 2, neutral	1.373	1.69	4.55E-06
Jmjd7	433466	jumonji domain containing 7	1.369	3.32	2.38E-10
Vmac	106639	vimentin-type intermediate filament associated coiled-coil protein	1.369	0.70	1.64E-04
Trpt1	107328	tRNA phosphotransferase 1	1.364	0.87	7.58E-03
Pir	69656	pirin	1.364	0.66	6.57E-03
C1galt1c1	59048	C1GALT1-specific chaperone 1	1.358	3.19	2.10E-10
Csrnp2	207785	cysteine-serine-rich nuclear protein 2	1.357	1.09	4.98E-11
Asb13	142688	ankyrin repeat and SOCS box-containing 13	1.354	2.00	2.76E-10
Rps2-ps10	667279	ribosomal protein S2, pseudogene 10	1.351	2.07	5.40E-04
Cep152	99100	centrosomal protein 152	1.349	1.32	1.02E-16
Fam98c	73833	family with sequence similarity 98, member C	1.346	3.22	7.09E-10
Mcat	223722	malonyl CoA:ACP acyltransferase (mitochondrial)	1.342	2.74	1.22E-10
Oas1e	231699	2'-5' oligoadenylate synthetase 1E	1.341	0.80	1.70E-03
Aspa	11484	aspartoacylase	1.340	0.72	1.17E-03
Tspan13	66109	tetraspanin 13	1.333	0.52	8.03E-03
Alkbh6	233065	alkB, alkylation repair homolog 6 (E. coli)	1.333	2.11	7.37E-05
Atg4a_2	666468	autophagy related 4A, cysteine peptidase	1.332	2.30	3.22E-07
Rn5s-ps1	19805	5S RNA, pseudogene 1	1.332	7.78	1.28E-02
Krtcap2	66059	keratinocyte associated protein 2	1.331	12.88	4.57E-18
Fbrsl1	381668	fibrosin-like 1	1.330	2.38	2.29E-36
Vsig10l	75690	ZV-set and immunoglobulin domain containing 10 like	1.329	0.89	1.48E-07
Rnu5g	97418	RNA, U5G small nuclear	1.324	236.17	7.58E-40
Tmem139	109218	transmembrane protein 139	1.319	3.05	3.41E-08
Zfp326	54367	zinc finger protein 326	1.319	2.21	2.88E-20

Official Gene Symbol	Entrez Gene ID	Gene description	Normalized Fold Change LOG2(Cryo-2 / Fresh-1)	FPKM Mean of six samples	p-value of Cryo-2 vs Fresh-1
Tex21	80384	testis expressed gene 21	1.319	0.58	6.25E-04
Tmem184b	223693	transmembrane protein 184b	1.318	1.16	1.06E-15
Ccpg1os	546143	cell cycle progression 1, opposite strand	1.317	2.47	1.04E-05
Cenpv	73139	centromere protein V	1.317	1.68	7.82E-06
LOC105244150	105244150	-	1.316	0.68	3.80E-02
Pbxip1	229534	pre B cell leukemia transcription factor interacting protein 1	1.315	0.76	3.03E-07
Syngr1	20972	synaptogyrin 1	1.312	2.97	7.58E-24
Hao2	56185	hydroxyacid oxidase 2	1.310	1.46	4.30E-07
Sfxn2	94279	sideroflexin 2	1.307	1.29	1.77E-08
Mfsd10	68294	major facilitator superfamily domain containing 10	1.300	2.67	1.28E-10
Tac2	21334	tachykinin 2	1.299	1.43	2.84E-03
Gpsm3	106512	G-protein signalling modulator 3 (AGS3-like, C. elegans)	1.296	0.70	1.09E-02
Ptk7	71461	PTK7 protein tyrosine kinase 7	1.293	0.55	7.03E-05
Fut8	53618	fucosyltransferase 8	1.288	2.77	2.88E-22
Dnajc1	13418	DnaJ (Hsp40) homolog, subfamily C, member 1	1.286	0.97	3.40E-15
BC029722	613262	cDNA sequence BC029722	1.285	0.53	4.35E-02
Hspb8	80888	heat shock protein 8	1.281	4.50	1.03E-15
Zdhhc12	66220	zinc finger, DHHC domain containing 12	1.279	5.32	1.92E-11
Aldob	230163	aldolase B, fructose-bisphosphate	1.277	1.39	8.51E-06
Filip1l	78749	filamin A interacting protein 1-like	1.276	0.51	7.98E-04
Fuom	69064	fucose mutarotase	1.276	0.51	1.17E-03
Tnks1bp1	228140	tankyrase 1 binding protein 1	1.272	0.77	1.36E-11
Slc35a5	74102	solute carrier family 35, member A5	1.269	1.88	1.18E-14
Rcan1	54720	regulator of calcineurin 1	1.268	3.33	8.81E-16
Ccdc92	215707	coiled-coil domain containing 92	1.266	3.12	2.02E-14
Tmem80	71448	transmembrane protein 80	1.259	0.66	5.85E-04
Phpt1	75454	phosphohistidine phosphatase 1	1.256	4.76	2.05E-05
Msrb3	320183	methionine sulfoxide reductase B3	1.256	1.22	1.77E-09
Ifngr1	15979	interferon gamma receptor 1	1.256	0.56	1.28E-02
Emb	13723	embigin	1.254	0.93	3.15E-05
Inf2	70435	inverted formin, FH2 and WH2 domain containing	1.253	0.86	1.07E-06
Caprin2	232560	caprin family member 2	1.251	2.18	2.29E-15
Gpr160	71862	G protein-coupled receptor 160	1.250	1.53	7.32E-07
Tbcb	66411	tubulin folding cofactor B	1.250	12.22	1.71E-25
Mapkapk5	17165	MAP kinase-activated protein kinase 5	1.247	8.45	7.58E-33
Lemd2	224640	LEM domain containing 2	1.243	0.91	1.65E-04
Fdxacb1	382137	ferredoxin-fold anticodon binding domain containing 1	1.243	1.80	5.15E-07
Mrpl14	68463	mitochondrial ribosomal protein L14	1.240	7.09	1.96E-10
Msl2_2	77853	male-specific lethal 2 homolog (Drosophila)	1.240	4.12	1.52E-24
Rufy1	216724	RUN and FYVE domain containing 1	1.237	2.16	1.54E-11
Wbscr27	79565	Williams Beuren syndrome chromosome region 27 (human)	1.235	2.89	8.00E-15
Timm17b	21855	translocase of inner mitochondrial membrane 17b	1.228	5.90	4.37E-19
Brip1	237911	BRCA1 interacting protein C-terminal helicase 1	1.222	2.04	1.38E-25
Mrpl52	68836	mitochondrial ribosomal protein L52	1.220	31.13	1.41E-18
Msl2_1	77853	male-specific lethal 2 homolog (Drosophila)	1.218	4.71	5.08E-06
Tst	22117	thiosulfate sulfurtransferase, mitochondrial	1.217	1.57	7.55E-04
Gna15	14676	guanine nucleotide binding protein, alpha 15	1.216	0.89	3.42E-04
Jakmip1	76071	janus kinase and microtubule interacting protein 1	1.216	0.72	1.43E-08

Official Gene Symbol	Entrez Gene ID	Gene description	Normalized Fold Change LOG2(Cryo-2 / Fresh-1)	FPKM Mean of six samples	p-value of Cryo-2 vs Fresh-1
Mrpl53	68499	mitochondrial ribosomal protein L53	1.216	17.37	1.38E-17
Spp1	20750	secreted phosphoprotein 1	1.216	12.04	3.52E-34
Plagl2	54711	pleiomorphic adenoma gene-like 2	1.211	1.21	2.78E-12
Serping1	12258	serine (or cysteine) peptidase inhibitor, clade G, member 1	1.211	1.71	3.16E-06
Fam53c	66306	family with sequence similarity 53, member C	1.205	2.43	1.24E-18
Armcx5	494468	armadillo repeat containing, X-linked 5	1.203	1.87	6.09E-09
Nudt8	66387	nudix (nucleoside diphosphate linked moiety X)-type motif 8	1.199	1.20	2.24E-02
Zfp850	100043772	zinc finger protein 850	1.196	1.11	1.03E-13
Ddhd1	114874	DDHD domain containing 1	1.192	2.32	7.78E-20
Mdp1	67881	magnesium-dependent phosphatase 1	1.190	2.01	9.27E-06
LOC100534325	100534325	expressed sequence AW146154 pseudogene	1.189	2.31	5.33E-05
Cdh3	12560	cadherin 3	1.188	0.62	4.95E-05
Ifngr2	15980	interferon gamma receptor 2	1.185	1.17	1.48E-04
Cfap45	71870	cilia and flagella associated protein 45	1.185	0.66	4.81E-03
Ccdc138	76138	coiled-coil domain containing 138	1.183	0.73	8.64E-05
Tmem171	380863	transmembrane protein 171	1.181	1.13	2.05E-04
LOC105246634	105246634	-	1.174	1.86	3.55E-05
Zfp865	319748	zinc finger protein 865	1.169	0.54	1.49E-04
Ooep	67968	oocyte expressed protein	1.168	6.31	3.33E-12
Zkscan17	268417	zinc finger with KRAB and SCAN domains 17	1.168	0.77	4.20E-06
Ptprtos	77645	protein tyrosine phosphatase, receptor type T, opposite strand	1.168	1.05	6.76E-06
Siah2	20439	seven in absentia 2	1.166	0.70	2.15E-03
Casp7	12369	caspase 7	1.162	1.26	7.48E-07
Camta1	100072	calmodulin binding transcription activator 1	1.162	0.57	1.62E-08
Atxn1	20238	ataxin 1	1.158	1.49	8.52E-33
Pdgfa	18590	platelet derived growth factor, alpha	1.158	9.63	3.67E-74
Cdc25c	12532	cell division cycle 25C	1.157	3.79	7.96E-15
Triml1	244448	tripartite motif family-like 1	1.157	1.10	3.44E-06
Depdc5	277854	DEP domain containing 5	1.155	0.72	1.54E-09
Zfp747	269997	zinc finger protein 747	1.155	2.06	7.82E-09
Trim26	22670	tripartite motif-containing 26	1.155	2.01	4.21E-11
Alox12	11684	arachidonate 12-lipoxygenase	1.154	2.01	6.59E-09
Ppm1k	243382	protein phosphatase 1K (PP2C domain containing)	1.154	1.32	2.46E-14
Ckap4	216197	cytoskeleton-associated protein 4	1.151	1.28	1.32E-05
Pacs1	107975	phosphofurin acidic cluster sorting protein 1	1.151	0.75	3.46E-06
Sft2d3	67158	SFT2 domain containing 3	1.149	1.35	1.70E-06
Btbd19	78611	BTB (POZ) domain containing 19	1.149	0.87	1.02E-07
ATP6	17705	-	1.148	9.70	1.84E-10
Nfic	18029	nuclear factor I/C	1.147	1.02	1.36E-09
Efna3	13638	ephrin A3	1.143	0.60	4.83E-03
Smyd2	226830	SET and MYND domain containing 2	1.142	2.64	2.21E-07
Adcy3	104111	adenylate cyclase 3	1.141	1.12	8.91E-09
Phf2	18676	PHD finger protein 2	1.140	0.89	8.07E-08
Cntln	338349	centlein, centrosomal protein	1.139	0.68	1.04E-09
Ddit4	74747	DNA-damage-inducible transcript 4	1.138	5.93	3.41E-14
Tm6sf1	107769	transmembrane 6 superfamily member 1	1.135	2.56	5.14E-10
Ippk	75678	inositol 1,3,4,5,6-pentakisphosphate 2-kinase	1.134	3.55	4.27E-16
Usp31	76179	ubiquitin specific peptidase 31	1.132	2.32	1.74E-30
Scrn3	74616	secernin 3	1.132	1.96	3.80E-10

Official Gene Symbol	Entrez Gene ID	Gene description	Normalized Fold Change LOG2(Cryo-2 / Fresh-1)	FPKM Mean of six samples	p-value of Cryo-2 vs Fresh-1
Map1b	17755	microtubule-associated protein 1B	1.129	0.60	5.23E-08
Creld1	171508	cysteine-rich with EGF-like domains 1	1.128	0.58	1.39E-02
Slc39a6	106957	solute carrier family 39 (metal ion transporter), member 6	1.125	1.75	1.03E-09
Dynll2	68097	dynein light chain LC8-type 2	1.124	9.09	1.14E-32
Rpl19-ps11	100041511	ribosomal protein L19, pseudogene 11	1.124	1.70	2.63E-02
Ebpl	68177	emopamil binding protein-like	1.122	1.97	1.35E-06
Cep85l	100038725	centrosomal protein 85-like	1.121	1.40	5.44E-11
Hook1	77963	hook homolog 1 (Drosophila)	1.121	1.03	2.27E-06
Gsta2	14858	glutathione S-transferase, alpha 2 (Yc2)	1.120	0.75	3.82E-02
Mir1983	100316716	microRNA 1983	1.118	12.18	3.56E-03
Lcmt1	30949	leucine carboxyl methyltransferase 1	1.118	2.74	1.98E-07
Pdzk1ip1	67182	PDZK1 interacting protein 1	1.117	3.65	4.81E-06
Pnkd	56695	paroxysmal nonkinesiogenic dyskinesia	1.117	2.56	4.94E-13
H2-DMb1	14999	histocompatibility 2, class II, locus Mb1	1.117	1.21	4.29E-03
Cdkn2c	12580	cyclin-dependent kinase inhibitor 2C (p18, inhibits CDK4)	1.115	2.08	1.28E-07
Rnf113a2	66381	ring finger protein 113A2	1.115	5.24	5.44E-11
Dtd1	66044	D-tyrosyl-tRNA deacylase 1	1.114	3.75	1.20E-07
Garem	381126	GRB2 associated, regulator of MAPK1	1.114	1.76	9.83E-13
Osbpl3	71720	oxysterol binding protein-like 3	1.113	0.64	9.07E-08
Myo10	17909	myosin X	1.112	1.09	1.08E-14
Xkr8	381560	X Kell blood group precursor related family member 8 homolog	1.111	1.19	2.96E-08
Fam154a	75811	family with sequence similarity 154, member A	1.111	1.19	5.79E-04
Rps6ka5	73086	ribosomal protein S6 kinase, polypeptide 5	1.111	0.72	7.64E-07
Mtcl1	68617	microtubule crosslinking factor 1	1.110	0.82	4.93E-08
LOC105244835	105244835	-	1.110	2.46	4.44E-24
Pigz	239827	phosphatidylinositol glycan anchor biosynthesis, class Z	1.109	1.43	1.05E-11
Rps16-ps2	100039355	ribosomal protein S16, pseudogene 2	1.109	38.00	4.76E-28
Heca	380629	headcase homolog (Drosophila)	1.109	1.71	1.15E-11
Smim22	432995	small integral membrane protein 22	1.108	7.81	5.17E-06
Rhou	69581	ras homolog gene family, member U	1.107	1.42	1.13E-07
Vaultrc5	378472	vault RNA component 5	1.106	52.67	6.91E-24
Tcea3	21401	transcription elongation factor A (SII), 3	1.104	2.28	6.46E-05
Chic1	12212	cysteine-rich hydrophobic domain 1	1.103	0.98	3.85E-11
Asb4	65255	ankyrin repeat and SOCS box-containing 4	1.102	0.51	2.67E-03
Crlf1	12931	cytokine receptor-like factor 1	1.101	0.52	3.85E-02
Timm10b	14356	translocase of inner mitochondrial membrane 10B	1.097	5.77	9.13E-10
Padi6	242726	peptidyl arginine deiminase, type VI	1.092	1.41	7.40E-05
Plk2	20620	polo-like kinase 2	1.092	2.24	1.33E-09
Acpp	56318	acid phosphatase, prostate	1.092	0.76	4.98E-07
Mir6240	102466620	microRNA 6240	1.092	13.79	4.21E-03
Pskh1	244631	protein serine kinase H1	1.091	1.90	1.55E-09
Abhd6	66082	abhydrolase domain containing 6	1.090	2.14	1.09E-08
Fam122a	68034	family with sequence similarity 122, member A	1.089	2.27	5.90E-06
Nos1	18125	nitric oxide synthase 1, neuronal	1.087	1.20	2.26E-17
Mrps12	24030	mitochondrial ribosomal protein S12	1.087	17.02	2.64E-17
Gng10	14700	guanine nucleotide binding protein (G protein), gamma 10	1.086	0.78	4.11E-02
Fam69a	67266	family with sequence similarity 69, member A	1.086	4.93	4.21E-18

Official Gene Symbol	Entrez Gene ID	Gene description	Normalized Fold Change LOG2(Cryo-2 / Fresh-1)	FPKM Mean of six samples	p-value of Cryo-2 vs Fresh-1
Cry2	12953	cryptochrome 2 (photolyase-like)	1.085	0.59	4.55E-04
Rab6b	270192	RAB6B, member RAS oncogene family	1.085	0.80	2.02E-05
Hist1h2aa	319163	histone cluster 1, H2aa	1.085	7.42	2.03E-05
Adcy9	11515	adenylate cyclase 9	1.085	0.70	5.13E-07
App	11820	amyloid beta (A4) precursor protein	1.085	2.56	1.06E-13
Telo2	71718	TEL2, telomere maintenance 2, homolog (S. cerevisiae)	1.081	0.50	4.40E-03
Yy2	100073351	Yy2 transcription factor	1.081	1.80	1.98E-07
Ppp1r13l	333654	protein phosphatase 1, regulatory (inhibitor) subunit 13 like	1.081	0.62	1.38E-04
Dhx34	71723	DEAH (Asp-Glu-Ala-His) box polypeptide 34	1.079	1.37	3.00E-10
Hist1h4f	319157	histone cluster 1, H4f	1.079	45.10	4.62E-20
Rny3	19874	RNA, Y3 small cytoplasmic (associated with Ro protein)	1.077	588.18	3.47E-51
Ptges2	96979	prostaglandin E synthase 2	1.076	4.27	2.78E-12
Ccbl1	70266	cysteine conjugate-beta lyase 1	1.075	1.14	2.79E-05
Trib3	228775	tribbles homolog 3 (Drosophila)	1.074	1.03	1.79E-03
Sept12	71089	septin 12	1.073	2.85	1.03E-10
Reep6	70335	receptor accessory protein 6	1.071	2.64	1.82E-08
n-R5s136	100861491	nuclear encoded rRNA 5S 136	1.071	######	1.75E-90
Recql4	79456	RecQ protein-like 4	1.071	1.22	2.84E-07
Tnfrsf12a	27279	tumor necrosis factor receptor superfamily, member 12a	1.070	1.35	1.76E-02
Cd2	12481	CD2 antigen	1.070	1.55	1.25E-02
Pcgf1	69837	polycomb group ring finger 1	1.069	2.82	3.26E-04
Elmod1	270162	ELMO/CED-12 domain containing 1	1.068	0.51	3.69E-03
Xlr3c	22446	X-linked lymphocyte-regulated 3C	1.068	0.94	9.18E-03
LOC105245483	105245483	-	1.067	0.69	2.52E-51
D10Jhu81e	28295	DNA segment, Chr 10, Johns Hopkins University 81 expressed	1.066	2.00	3.41E-04
Coro2a	107684	coronin, actin binding protein 2A	1.065	1.14	5.39E-07
Wdr83os	414077	WD repeat domain 83 opposite strand	1.065	13.05	1.16E-11
Cipc	217732	CLOCK interacting protein, circadian	1.063	0.69	5.86E-06
Tmem115	56395	transmembrane protein 115	1.063	1.41	1.14E-04
Rcn3	52377	reticulocalbin 3, EF-hand calcium binding domain	1.062	0.86	1.32E-02
Tnrc6c	217351	trinucleotide repeat containing 6C	1.060	0.82	4.91E-10
Pcnx	54604	pecanex homolog (Drosophila)	1.058	1.64	1.30E-24
Mpl	17480	myeloproliferative leukemia virus oncogene	1.057	0.65	2.61E-03
Taf8	63856	TAF8 RNA polymerase II, TATA box binding protein (TBP)-associated factorq	1.055	1.22	9.21E-10
Zfp9	22750	zinc finger protein 9	1.054	1.53	6.80E-09
Capn5	12337	calpain 5	1.054	2.13	4.80E-16
Lnx2	140887	ligand of numb-protein X 2	1.054	2.45	1.05E-13
Mycl	16918	v-myc myelocytomatosis viral oncogene homolog, lung carcinoma derived (avian)	1.053	0.84	5.76E-05
Snord13	100217422	small nucleolar RNA, C/D box 13	1.053	84.95	2.62E-11
Fadd	14082	Fas (TNFRSF6)-associated via death domain	1.051	1.02	1.45E-04
Trip11	109181	thyroid hormone receptor interactor 11	1.049	1.69	1.54E-19
Dhx57	106794	DEAH (Asp-Glu-Ala-Asp/His) box polypeptide 57	1.048	1.69	4.73E-12
Prr11	270906	proline rich 11	1.048	4.34	1.18E-20
Ctu1	233189	cytosolic thiouridylase subunit 1 homolog (S. pombe)	1.048	2.17	1.67E-07

Official Gene Symbol	Entrez Gene ID	Gene description	Normalized Fold Change LOG2(Cryo-2 / Fresh-1)	FPKM Mean of six samples	p-value of Cryo-2 vs Fresh-1
Phlda3	27280	pleckstrin homology-like domain, family A, member 3	1.048	3.73	1.11E-07
Vkorc1	27973	vitamin K epoxide reductase complex, subunit 1	1.047	7.66	1.48E-07
Aldh3a2	11671	aldehyde dehydrogenase family 3, subfamily A2	1.047	1.62	5.11E-16
Tgds	76355	TDP-glucose 4,6-dehydratase	1.046	2.01	1.95E-06
Btbd18	100270744	BTB (POZ) domain containing 18	1.045	0.95	1.15E-04
Zc3hav1l	209032	zinc finger CCCH-type, antiviral 1-like	1.045	0.60	1.43E-05
Rasa2	114713	RAS p21 protein activator 2	1.045	1.50	1.58E-11
LOC105245362	105245362	-	1.041	0.63	2.57E-09
Zfp948	381066	zinc finger protein 948	1.040	0.95	1.02E-04
Jag2	16450	jagged 2	1.039	0.82	2.61E-06
Atp5g1	11951	ATP synthase, H+ transporting, mitochondrial F0 complex, subunit C1 (subunit 9)	1.035	45.73	3.64E-40
Mgat4b	103534	mannoside acetylglucosaminyltransferase 4, isoenzyme B	1.033	3.81	1.93E-11
Mknk2	17347	MAP kinase-interacting serine/threonine kinase 2	1.033	2.94	3.65E-11
Wdr89	72338	WD repeat domain 89	1.032	8.14	4.29E-25
Pomk	74653	protein-O-mannose kinase	1.032	0.85	1.54E-04
Zscan4b	665780	zinc finger and SCAN domain containing 4B	1.029	0.67	2.31E-02
Fgd1	14163	FYVE, RhoGEF and PH domain containing 1	1.028	0.92	2.09E-05
Csta1	209294	cystatin A1	1.025	7.02	5.86E-21
Agfg2	231801	ArfGAP with FG repeats 2	1.025	1.34	1.04E-05
Ric1	226089	RAB6A GEF complex partner 1	1.024	3.30	3.74E-22
Map3k5	26408	mitogen-activated protein kinase kinase kinase 5	1.023	1.59	6.16E-12
Gclc	14629	glutamate-cysteine ligase, catalytic subunit	1.023	2.43	1.27E-11
Akap7	432442	A kinase (PRKA) anchor protein 7	1.022	1.28	4.79E-12
Cenpb	12616	centromere protein B	1.018	0.97	1.20E-03
Cln5	211286	ceroid-lipofuscinosis, neuronal 5	1.016	4.34	2.39E-16
Tacstd2	56753	tumor-associated calcium signal transducer 2	1.015	4.05	5.29E-09
Hist1h2bp	319188	histone cluster 1, H2bp	1.014	6.58	2.65E-06
Igsf9b	235086	immunoglobulin superfamily, member 9B	1.010	0.50	1.65E-10
Arf5	11844	ADP-ribosylation factor 5	1.009	4.57	4.16E-06
Slc16a13	69309	solute carrier family 16 (monocarboxylic acid transporters), member 13	1.009	1.05	6.01E-06
Nucb2	53322	nucleobindin 2	1.008	2.26	1.45E-09
Rpp25	102614	ribonuclease P/MRP 25 subunit	1.008	3.08	7.58E-06
Sec22a	317717	SEC22 vesicle trafficking protein homolog A (S. cerevisiae)	1.005	2.65	3.29E-12
Cela2a	13706	chymotrypsin-like elastase family, member 2A	1.005	1.07	4.35E-02
Rpl27-ps3	621100	ribosomal protein L27, pseudogene 3	1.003	8.86	4.20E-05
Acer1	171168	alkaline ceramidase 1	1.001	1.26	2.64E-04
Zscan26	432731	zinc finger and SCAN domain containing 26	0.999	4.40	4.64E-22
Asap1	13196	ArfGAP with SH3 domain, ankyrin repeat and PH domain1	0.999	0.62	3.30E-06
Ly6g6c	68468	lymphocyte antigen 6 complex, locus G6C	0.999	1.14	4.26E-02
Dusp7	235584	dual specificity phosphatase 7	0.998	0.82	6.97E-04
Tpst1	22021	protein-tyrosine sulfotransferase 1	0.998	2.70	6.64E-07
Sccpdh	109232	saccharopine dehydrogenase (putative)	0.997	2.29	1.21E-05
LOC105244007	105244007	-	0.997	0.73	1.27E-06
Agbl5	231093	ATP/GTP binding protein-like 5	0.993	0.76	6.43E-05
Zdhhc13	243983	zinc finger, DHHC domain containing 13	0.992	3.01	2.29E-09
Fam46b	100342	family with sequence similarity 46, member B	0.991	0.51	3.04E-02

Official Gene Symbol	Entrez Gene ID	Gene description	Normalized Fold Change LOG2(Cryo-2 / Fresh-1)	FPKM Mean of six samples	p-value of Cryo-2 vs Fresh-1
Syne3	212073	spectrin repeat containing, nuclear envelope family member 3	0.990	0.66	1.78E-05
Igdcc3	19289	immunoglobulin superfamily, DCC subclass, member 3	0.990	0.51	2.49E-03
Cystm1	66060	cysteine-rich transmembrane module containing 1	0.989	12.57	3.30E-09
Pisd	320951	phosphatidylserine decarboxylase	0.988	2.90	2.33E-10
Mettl10	72096	methyltransferase like 10	0.986	1.27	5.38E-04
Plod3	26433	procollagen-lysine, 2-oxoglutarate 5-dioxygenase 3	0.985	1.59	3.76E-07
Rpl35a-ps2	100042363	ribosomal protein L35A, pseudogene 2	0.984	22.09	8.02E-12
Mb21d2	239796	Mab-21 domain containing 2	0.983	0.71	1.37E-03
Tpmt	22017	thiopurine methyltransferase	0.983	1.54	7.59E-07
Map3k1	26401	mitogen-activated protein kinase kinase kinase 1	0.981	1.16	3.30E-14
Snhg12	100039864	small nucleolar RNA host gene 12	0.980	19.99	2.57E-37
Qtrtd1	106248	queuine tRNA-ribosyltransferase domain containing 1	0.980	1.38	1.03E-05
Crip1	12925	cysteine-rich protein 1 (intestinal)	0.977	30.01	5.33E-14
Clcn2	12724	chloride channel 2	0.975	1.26	8.78E-06
Psme2	19188	proteasome (prosome, macropain) activator subunit 2 (PA28 beta)	0.975	2.07	5.50E-03
Gpr116	224792	G protein-coupled receptor 116	0.975	0.70	3.32E-07
Dhrs1	52585	dehydrogenase/reductase (SDR family) member 1	0.975	0.80	3.57E-02
Rfx1	19724	regulatory factor X, 1 (influences HLA class II expression)	0.975	1.43	1.22E-08
Arhgap17	70497	Rho GTPase activating protein 17	0.972	2.45	4.90E-16
Atrn	11990	attractin	0.971	1.85	2.82E-16
Ginm1	215751	glycoprotein integral membrane 1	0.970	7.44	2.90E-13
Zfp65	235907	zinc finger protein 65	0.968	3.19	1.44E-13
Eif4ebp1	13685	eukaryotic translation initiation factor 4E binding protein 1	0.967	63.61	2.84E-45
Pigh	110417	phosphatidylinositol glycan anchor biosynthesis, class H	0.967	2.14	3.75E-06
Farp2	227377	FERM, RhoGEF and pleckstrin domain protein 2	0.965	0.58	3.90E-03
Fdx1	14148	ferredoxin 1	0.965	3.55	1.22E-12
Foxm1	14235	forkhead box M1	0.964	3.24	4.36E-15
Rnu3b-ps1	19852	U3B small nuclear RNA pseudogene 1	0.964	20.45	2.58E-05
Agtrap	11610	angiotensin II, type I receptor-associated protein	0.964	3.17	9.54E-14
Coa3	52469	cytochrome C oxidase assembly factor 3	0.963	15.18	1.09E-12
Rny1	19872	RNA, Y1 small cytoplasmic, Ro-associated	0.962	137.04	2.93E-15
Pycard	66824	PYD and CARD domain containing	0.961	2.73	3.56E-06
Bbs5	72569	Bardet-Biedl syndrome 5 (human)	0.960	1.29	1.56E-04
Mok	26448	MOK protein kinase	0.959	0.62	2.00E-03
Ptbp2	56195	polypyrimidine tract binding protein 2	0.959	2.13	2.37E-09
Klf16	118445	Kruppel-like factor 16	0.957	1.97	6.71E-06
Fam83b	208994	family with sequence similarity 83, member B	0.957	0.86	5.27E-07
Parp4	328417	poly (ADP-ribose) polymerase family, member 4	0.956	2.23	2.16E-15
Cdk14	18647	cyclin-dependent kinase 14	0.956	1.02	4.40E-07
Gan	209239	giant axonal neuropathy	0.954	2.03	2.49E-06
Chpf	74241	chondroitin polymerizing factor	0.953	1.05	4.26E-04
Zfp712	78251	zinc finger protein 712	0.953	0.93	7.67E-04
Mif	17319	macrophage migration inhibitory factor	0.952	81.83	4.68E-38
Tprn	97031	taperin	0.952	4.55	8.85E-13
Mars2	212679	methionine-tRNA synthetase 2 (mitochondrial)	0.952	1.40	4.68E-05

Official Gene Symbol	Entrez Gene ID	Gene description	Normalized Fold Change LOG2(Cryo-2 / Fresh-1)	FPKM Mean of six samples	p-value of Cryo-2 vs Fresh-1
Peg10	170676	paternally expressed 10	0.951	6.62	1.85E-38
n-R5s100	100861471	nuclear encoded rRNA 5S 100	0.951	######	1.53E-11
Higd2a	67044	HIG1 domain family, member 2A	0.950	15.98	1.07E-10
Commd2	52245	COMM domain containing 2	0.949	4.80	7.77E-14
Abat	268860	4-aminobutyrate aminotransferase	0.948	0.87	5.75E-05
Tmem109	68539	transmembrane protein 109	0.947	1.63	4.01E-05
N6amt2	68043	N-6 adenine-specific DNA methyltransferase 2 (putative)	0.946	5.91	1.44E-08
Apbb1	11785	amyloid beta (A4) precursor protein-binding, family B, member 1	0.945	1.84	3.29E-06
Mob2	101513	MOB kinase activator 2	0.942	1.96	5.60E-06
Pofut2	80294	protein O-fucosyltransferase 2	0.941	2.29	6.39E-06
Mrpl34	94065	mitochondrial ribosomal protein L34	0.940	16.52	7.07E-10
Rps12-ps9	100503211	ribosomal protein S12, pseudogene 9	0.936	4.34	4.41E-03
Snora15	100113364	small nucleolar RNA, H/ACA box 15	0.935	12.81	1.70E-02
Angptl4	57875	angiopoietin-like 4	0.934	2.35	2.09E-05
Basp1	70350	brain abundant, membrane attached signal protein 1	0.934	6.00	7.41E-09
AK010878	100233175	cDNA sequence AK010878	0.934	2.85	2.41E-04
Tmem177	66343	transmembrane protein 177	0.933	1.19	4.94E-05
Man2c1	73744	mannosidase, alpha, class 2C, member 1	0.932	0.88	2.06E-04
Rnf114	81018	ring finger protein 114	0.932	9.90	2.81E-30
Tmem201	230917	transmembrane protein 201	0.931	2.30	8.02E-15
Otx2	18424	orthodenticle homolog 2	0.930	1.00	4.51E-04
Ndufa5	68202	NADH dehydrogenase (ubiquinone) 1 alpha subcomplex, 5	0.929	20.09	3.68E-09
Fbxo9	71538	f-box protein 9	0.929	4.85	3.00E-11
Zfp708	432769	zinc finger protein 708	0.927	2.08	1.87E-05
Polh	80905	polymerase (DNA directed), eta (RAD 30 related)	0.927	0.70	2.89E-03
Ilkap	67444	integrin-linked kinase-associated serine/threonine phosphatase 2C	0.926	1.79	6.23E-07
Tmem110	69179	transmembrane protein 110	0.925	1.73	1.57E-06
Map2k7	26400	mitogen-activated protein kinase kinase 7	0.925	1.90	1.12E-07
Dgat1	13350	diacylglycerol O-acyltransferase 1	0.925	2.38	1.01E-06
Bre	107976	brain and reproductive organ-expressed protein	0.923	8.67	1.53E-15
Sap30l	50724	SAP30-like	0.923	12.67	3.68E-17
Magi2	50791	membrane associated guanylate kinase, WW and PDZ domain containing 2	0.922	0.52	2.27E-05
Prps2	110639	phosphoribosyl pyrophosphate synthetase 2	0.921	0.52	1.43E-03
Atp5k	11958	ATP synthase, H+ transporting, mitochondrial F1F0 complex, subunit E	0.921	42.96	2.52E-11
Zfp931	353208	zinc finger protein 931	0.921	2.67	1.04E-05
Slc20a2	20516	solute carrier family 20, member 2	0.921	1.53	8.46E-07
Stradb	227154	STE20-related kinase adaptor beta	0.920	2.83	2.99E-07
Utf1	22286	undifferentiated embryonic cell transcription factor 1	0.919	7.18	4.10E-09
Cox16	66272	cytochrome c oxidase assembly protein 16	0.918	7.88	5.26E-16
Peli2	93834	pellino 2	0.918	0.91	8.67E-07
Ccdc91	67015	coiled-coil domain containing 91	0.917	4.56	7.72E-13
Fam185a	330050	family with sequence similarity 185, member A	0.915	1.30	1.65E-06
Psenen	66340	presenilin enhancer 2 homolog (C. elegans)	0.915	28.06	3.18E-11
Zkscan8	93681	zinc finger with KRAB and SCAN domains 8	0.915	0.88	2.48E-07
Rhod	11854	ras homolog gene family, member D	0.914	0.95	4.20E-02

Official Gene Symbol	Entrez Gene ID	Gene description	Normalized Fold Change LOG2(Cryo-2 / Fresh-1)	FPKM Mean of six samples	p-value of Cryo-2 vs Fresh-1
Ccdc163	68394	coiled-coil domain containing 163	0.914	1.49	8.52E-04
Mrpl47	74600	mitochondrial ribosomal protein L47	0.914	32.87	4.22E-27
Sbk1	104175	SH3-binding kinase 1	0.913	0.75	1.24E-03
Gtf2a2	235459	general transcription factor II A, 2	0.912	4.37	1.22E-16
Sgms1	208449	sphingomyelin synthase 1	0.912	1.73	1.72E-10
Fbxo7	69754	F-box protein 7	0.910	1.26	7.71E-04
Nat6	56441	N-acetyltransferase 6	0.910	1.33	2.01E-03
Palm	18483	paralemmin	0.909	1.07	1.79E-03
B2m	12010	beta-2 microglobulin	0.909	26.67	5.94E-22
AI987944	233168	expressed sequence AI987944	0.907	1.14	3.75E-05
Map3k6	53608	mitogen-activated protein kinase kinase kinase 6	0.905	0.54	7.85E-04
Pglyrp1	21946	peptidoglycan recognition protein 1	0.903	1.69	4.91E-02
LOC105242851	105242851	-	0.902	1.20	7.96E-04
Gmip	78816	Gem-interacting protein	0.900	0.69	1.46E-03
Axdnd1	77352	-	0.899	0.53	2.62E-02
Rpl35a-ps3	100043341	ribosomal protein L35A, pseudogene 3	0.898	8.48	5.64E-04
Atf6b	12915	activating transcription factor 6 beta	0.897	2.15	1.56E-05
Hist1h4j	319159	histone cluster 1, H4j	0.896	6.96	1.41E-02
Rnf121	75212	ring finger protein 121	0.895	1.85	9.88E-06
Emc9	85308	ER membrane protein complex subunit 9	0.893	2.78	2.86E-03
Tex30	75623	testis expressed 30	0.892	7.49	3.84E-10
Il17rc	171095	interleukin 17 receptor C	0.892	0.78	1.21E-02
Fank1	66930	fibronectin type 3 and ankyrin repeat domains 1	0.892	1.83	5.31E-04
Errfi1	74155	ERBB receptor feedback inhibitor 1	0.890	2.35	6.54E-08
Spsb3	79043	splA/ryanodine receptor domain and SOCS box containing 3	0.890	1.30	3.41E-04
Cox10	70383	cytochrome c oxidase assembly protein 10	0.890	6.57	2.20E-17
Chchd7	66433	coiled-coil-helix-coiled-coil-helix domain containing 7	0.889	3.36	1.75E-03
Tor1aip1	208263	torsin A interacting protein 1	0.889	3.84	1.61E-16
Lpcat4	99010	lysophosphatidylcholine acyltransferase 4	0.886	4.62	2.87E-08
Proser3	333193	proline and serine rich 3	0.884	1.09	2.62E-03
Kmt2a	214162	lysine (K)-specific methyltransferase 2A	0.883	2.77	1.09E-36
Ccdc88a	108686	coiled coil domain containing 88A	0.883	1.60	4.99E-17
Baiap2	108100	brain-specific angiogenesis inhibitor 1-associated protein 2	0.882	0.57	5.68E-04
Irf2bp2	270110	interferon regulatory factor 2 binding protein 2	0.881	1.53	1.38E-06
Dcxr	67880	dicarbonyl L-xylulose reductase	0.880	2.47	2.17E-03
Ccdc64	75665	coiled-coil domain containing 64	0.880	0.70	1.18E-03
Zcchc2	227449	zinc finger, CCHC domain containing 2	0.880	2.51	2.24E-14
Ndufb11	104130	NADH dehydrogenase (ubiquinone) 1 beta subcomplex, 11	0.878	29.24	2.18E-21
Nfkbiz	80859	nuclear factor of kappa light polypeptide gene enhancer in B cells inhibitor, zeta	0.877	0.78	1.71E-03
Zp2	22787	zona pellucida glycoprotein 2	0.877	1.08	5.45E-03
Mgst3	66447	microsomal glutathione S-transferase 3	0.876	11.15	1.03E-06
Efhc2	74405	EF-hand domain (C-terminal) containing 2	0.872	0.58	1.36E-02
Pgls	66171	6-phosphogluconolactonase	0.872	1.61	1.48E-02
Zdhhc4	72881	zinc finger, DHHC domain containing 4	0.871	6.86	1.87E-11
Lace1	215951	lactation elevated 1	0.871	1.71	1.80E-05
Snrpf	69878	small nuclear ribonucleoprotein polypeptide F	0.871	29.78	2.28E-21
Shroom4	208431	shroom family member 4	0.871	0.83	6.90E-08
Shisa5	66940	shisa family member 5	0.870	1.67	1.66E-04

Official Gene Symbol	Entrez Gene ID	Gene description	Normalized Fold Change LOG2(Cryo-2 / Fresh-1)	FPKM Mean of six samples	p-value of Cryo-2 vs Fresh-1
Kremen2	73016	kringle containing transmembrane protein 2	0.870	0.77	1.33E-02
Slc25a28	246696	solute carrier family 25, member 28	0.869	2.30	2.34E-04
LOC101055907	101055907	-	0.869	0.87	4.07E-04
Ghdc	80860	GH3 domain containing	0.867	5.49	3.82E-10
Sp2	78912	Sp2 transcription factor	0.864	1.54	1.23E-07
Cenpm	66570	centromere protein M	0.864	3.07	6.55E-05
Tmem199	195040	transmembrane protein 199	0.863	9.75	2.13E-11
Raph1	77300	Ras association (RalGDS/AF-6) and pleckstrin homology domains 1	0.863	3.59	2.04E-28
Ptpn9	56294	protein tyrosine phosphatase, non-receptor type 9	0.863	0.79	3.75E-03
Socs2	216233	suppressor of cytokine signaling 2	0.860	0.88	7.73E-09
Zfp105	22646	zinc finger protein 105	0.860	1.78	4.37E-04
Hmg20b	15353	high mobility group 20B	0.857	7.93	1.43E-12
Pawr	114774	PRKC, apoptosis, WT1, regulator	0.856	19.07	2.98E-27
Tpm3-rs7	621054	tropomyosin 3, related sequence 7	0.856	0.62	4.61E-02
Nudt2	66401	nudix (nucleoside diphosphate linked moiety X)-type motif 2	0.855	9.12	1.60E-09
Ago4	76850	argonaute RISC catalytic subunit 4	0.854	0.57	4.14E-04
Fkbp2	14227	FK506 binding protein 2	0.854	5.70	2.99E-07
Hsdl2	72479	hydroxysteroid dehydrogenase like 2	0.853	3.61	7.04E-11
Cyb5a	109672	cytochrome b5 type A (microsomal)	0.852	9.40	8.09E-07
Rab26os	75614	RAB26, member RAS oncogene family, opposite strand	0.852	35.79	1.95E-13
Itm2a	16431	integral membrane protein 2A	0.852	1.52	5.08E-03
Bcl2l12	75736	BCL2-like 12 (proline rich)	0.852	2.43	2.56E-03
Ccdc34	68201	coiled-coil domain containing 34	0.852	4.36	1.39E-15
Atp6v0c-ps2	100039636	ATPase, H+ transporting, lysosomal V0 subunit C, pseudogene 2	0.851	12.38	5.66E-11
Slc38a2	67760	solute carrier family 38, member 2	0.851	1.10	1.12E-04
Cd99l2	171486	CD99 antigen-like 2	0.850	1.36	2.30E-04
Spred2	114716	sprouty-related, EVH1 domain containing 2	0.849	2.43	1.35E-19
Ddit3	13198	DNA-damage inducible transcript 3	0.849	5.40	5.49E-05
Ankrd16	320816	ankyrin repeat domain 16	0.849	0.70	1.72E-02
Tmem237	381259	transmembrane protein 237	0.848	0.85	1.15E-02
Dimt1	66254	DIM1 dimethyladenosine transferase 1-like (S. cerevisiae)	0.848	4.65	2.99E-10
Mex3a	72640	mex3 homolog A (C. elegans)	0.848	0.53	2.70E-03
Cisd3	217149	CDGSH iron sulfur domain 3	0.848	1.81	4.41E-02
Irf3	54131	interferon regulatory factor 3	0.848	3.91	6.00E-08
Flrt1	396184	fibronectin leucine rich transmembrane protein 1	0.847	0.79	6.69E-10
Pla2g10	26565	phospholipase A2, group X	0.846	2.81	2.48E-04
Ankrd10	102334	ankyrin repeat domain 10	0.846	3.06	2.03E-13
Mep1b	17288	meprin 1 beta	0.845	2.19	4.23E-05
Cdyl	12593	chromodomain protein, Y chromosome-like	0.845	2.88	3.99E-10
Cenpu	71876	centromere protein U	0.843	2.01	3.07E-08
Mfap5	50530	microfibrillar associated protein 5	0.843	0.96	4.42E-02
St3gal6	54613	ST3 beta-galactoside alpha-2,3-sialyltransferase 6	0.843	0.56	4.74E-04
Pdik1l	230809	PDLIM1 interacting kinase 1 like	0.842	1.48	2.96E-07
D17Wsu92e	224647	DNA segment, Chr 17, Wayne State University 92, expressed	0.839	4.92	4.63E-14
Tsix	22097	X (inactive)-specific transcript, opposite strand	0.839	1.16	1.03E-39
Ctps2	55936	cytidine 5'-triphosphate synthase 2	0.839	1.36	1.39E-07

Official Gene Symbol	Entrez Gene ID	Gene description	Normalized Fold Change LOG2(Cryo-2 / Fresh-1)	FPKM Mean of six samples	p-value of Cryo-2 vs Fresh-1
Ralgapa1	56784	Ral GTPase activating protein, alpha subunit 1	0.836	2.05	4.02E-12
Ubl5	66177	ubiquitin-like 5	0.835	13.11	6.34E-22
Mrpl23	19935	mitochondrial ribosomal protein L23	0.835	7.39	2.27E-08
Snx18	170625	sorting nexin 18	0.834	3.33	1.48E-12
Zdhhc7	102193	zinc finger, DHHC domain containing 7	0.834	2.09	1.10E-05
Foxj2	60611	forkhead box J2	0.834	0.77	5.31E-04
Dgkq	110524	diacylglycerol kinase, theta	0.833	0.75	5.51E-04
Coro1b	23789	coronin, actin binding protein 1B	0.833	9.68	4.38E-14
Abcg2	26357	ATP-binding cassette, sub-family G (WHITE), member 2	0.832	0.84	9.74E-03
Snhg9	73474	small nucleolar RNA host gene 9	0.830	38.68	4.05E-11
Xlr4c	72891	X-linked lymphocyte-regulated 4C	0.830	7.34	8.63E-07
Mkl2	239719	MKL/myocardin-like 2	0.829	0.85	1.00E-10
Pigc	67292	phosphatidylinositol glycan anchor biosynthesis, class C	0.825	1.00	1.59E-03
Ndufa1	54405	NADH dehydrogenase (ubiquinone) 1 alpha subcomplex, 1	0.825	9.76	1.11E-04
Cln3	12752	ceroid lipofuscinosis, neuronal 3, juvenile (Batten, Spielmeyer-Vogt disease)	0.825	1.78	1.52E-05
Arrb1	109689	arrestin, beta 1	0.823	0.75	2.30E-05
Inpp5e	64436	inositol polyphosphate-5-phosphatase E	0.822	1.18	6.08E-05
Chd1l	68058	chromodomain helicase DNA binding protein 1-like	0.820	1.16	1.08E-03
Alg3	208624	asparagine-linked glycosylation 3 (alpha-1,3-mannosyltransferase)	0.820	5.68	2.25E-06
Nkiras1	69721	NFKB inhibitor interacting Ras-like protein 1	0.820	0.90	2.94E-04
Ndufaf5	69487	NADH dehydrogenase (ubiquinone) complex I, assembly factor 5	0.818	1.41	4.92E-04
Snrnp35	76167	small nuclear ribonucleoprotein 35 (U11/U12)	0.818	2.89	7.58E-05
Apex2	77622	apurinic/apyrimidinic endonuclease 2	0.818	1.41	8.38E-06
Arl6ip5	65106	ADP-ribosylation factor-like 6 interacting protein 5	0.817	4.95	9.54E-06
Plekhm1	353047	pleckstrin homology domain containing, family M (with RUN domain) member 1	0.817	3.41	2.81E-14
Snhg1	83673	small nucleolar RNA host gene 1	0.817	36.46	7.09E-46
Pigs	276846	phosphatidylinositol glycan anchor biosynthesis, class S	0.816	3.51	1.85E-07
Tprkb	69786	Tp53rk binding protein	0.814	6.44	3.79E-14
Id3	15903	inhibitor of DNA binding 3	0.814	6.39	1.31E-05
Ribc1	66611	RIB43A domain with coiled-coils 1	0.812	1.63	7.49E-05
Atp6v1g1	66290	ATPase, H+ transporting, lysosomal V1 subunit G1	0.811	44.72	9.93E-31
Romo1	67067	reactive oxygen species modulator 1	0.811	9.67	2.31E-05
Rps19bp1	66538	ribosomal protein S19 binding protein 1	0.810	15.30	1.83E-09
Nfya	18044	nuclear transcription factor-Y alpha	0.809	3.54	2.05E-10
Gxylt1	223827	glucoside xylosyltransferase 1	0.809	3.36	2.44E-16
Hint2	68917	histidine triad nucleotide binding protein 2	0.809	2.97	2.46E-02
Hist1h2bn	319187	histone cluster 1, H2bn	0.809	69.01	9.42E-17
Cep250	16328	centrosomal protein 250	0.808	1.81	2.85E-11
Bcor	71458	BCL6 interacting corepressor	0.808	3.08	2.20E-15
Chchd5	66170	coiled-coil-helix-coiled-coil-helix domain containing 5	0.807	21.42	8.69E-19
Upf3a	67031	UPF3 regulator of nonsense transcripts homolog A (yeast)	0.807	5.61	1.01E-08
Zfp36l1	12192	zinc finger protein 36, C3H type-like 1	0.807	3.26	9.01E-08
Tmem170	66817	transmembrane protein 170	0.807	5.29	4.32E-13

Official Gene Symbol	Entrez Gene ID	Gene description	Normalized Fold Change LOG2(Cryo-2 / Fresh-1)	FPKM Mean of six samples	p-value of Cryo-2 vs Fresh-1
Hes1	15205	hairy and enhancer of split 1 (Drosophila)	0.806	4.98	3.76E-07
Slc7a8	50934	solute carrier family 7 (cationic amino acid transporter, y+ system), member 8	0.805	0.54	2.17E-02
Pafah2	100163	platelet-activating factor acetylhydrolase 2	0.804	2.40	4.38E-07
LOC102639505	102639505	-	0.804	2.04	1.01E-02
Ift74	67694	intraflagellar transport 74	0.803	1.73	1.90E-06
Dnajc30	66114	DnaJ (Hsp40) homolog, subfamily C, member 30	0.803	2.83	3.91E-03
Nkx6-2	14912	NK6 homeobox 2	0.802	2.81	1.13E-03
Ndufs8	225887	NADH dehydrogenase (ubiquinone) Fe-S protein 8	0.800	26.04	1.08E-15
Pqlc1	66943	PQ loop repeat containing 1	0.799	1.57	1.77E-06
Amz2	13929	archaelysin family metallopeptidase 2	0.798	3.45	9.13E-08
Cox6a1	12861	cytochrome c oxidase subunit VIa polypeptide 1	0.796	107.67	5.87E-30
Trmt11	73681	tRNA methyltransferase 11	0.795	2.69	1.08E-06
Atxn7	246103	ataxin 7	0.795	3.94	4.73E-19
Snx9	66616	sorting nexin 9	0.795	6.32	4.82E-10
Ppwd1	238831	peptidylprolyl isomerase domain and WD repeat containing 1	0.794	10.25	1.43E-16
Tm2d2	69742	TM2 domain containing 2	0.793	2.85	1.10E-03
Tmem79	71913	transmembrane protein 79	0.792	3.24	3.58E-06
Chdh	218865	choline dehydrogenase	0.790	1.15	1.69E-05
Pomt2	217734	protein-O-mannosyltransferase 2	0.789	1.17	4.50E-05
Fbxo22	71999	F-box protein 22	0.789	12.07	1.44E-20
Ephx1	13849	epoxide hydrolase 1, microsomal	0.787	1.93	2.30E-03
Mtus2	77521	microtubule associated tumor suppressor candidate 2	0.787	1.33	4.67E-09
Tpst2	22022	protein-tyrosine sulfotransferase 2	0.786	2.97	2.19E-05
Ttll1	319953	tubulin tyrosine ligase-like 1	0.786	2.65	3.21E-05
Cd209c	170776	CD209c antigen	0.785	1.30	1.26E-02
Hist1h2an	319170	histone cluster 1, H2an	0.785	32.75	9.52E-11
Cox14	66379	cytochrome c oxidase assembly protein 14	0.784	20.97	3.44E-11
Sart3	53890	squamous cell carcinoma antigen recognized by T cells 3	0.784	5.57	1.27E-16
Pdxp	57028	pyridoxal (pyridoxine, vitamin B6) phosphatase	0.782	1.31	1.94E-02
Ccdc18	73254	coiled-coil domain containing 18	0.782	2.91	9.02E-13
Minos1	433771	mitochondrial inner membrane organizing system 1	0.782	12.61	1.11E-19
Chic2	74277	cysteine-rich hydrophobic domain 2	0.782	11.12	7.41E-09
Rbpms	19663	RNA binding protein gene with multiple splicing	0.782	9.80	5.19E-26
Bin2	668218	bridging integrator 2	0.781	16.83	8.66E-23
Glipr2	384009	GLI pathogenesis-related 2	0.780	6.54	8.98E-10
Ptprk	19272	protein tyrosine phosphatase, receptor type, K	0.780	1.90	1.41E-09
Prorsd1	67939	prolyl-tRNA synthetase domain containing 1	0.780	2.48	2.67E-04
Ephb3	13845	Eph receptor B3	0.778	4.79	1.42E-17
Nprl2	56032	nitrogen permease regulator-like 2	0.778	2.41	1.42E-03
Ice1	218333	interactor of little elongation complex ELL subunit 1	0.778	4.41	2.34E-18
H1fx	243529	H1 histone family, member X	0.776	1.45	4.64E-02
Cdkn1a	12575	cyclin-dependent kinase inhibitor 1A (P21)	0.775	5.76	8.93E-09
Efemp1	216616	epidermal growth factor-containing fibulin-like extracellular matrix protein 1	0.775	4.42	1.38E-06
Mrps25	64658	mitochondrial ribosomal protein S25	0.775	1.62	1.41E-07
Usp54	78787	ubiquitin specific peptidase 54	0.775	0.85	2.20E-06
Nr1d2	353187	nuclear receptor subfamily 1, group D, member 2	0.775	0.62	6.31E-03
Mrpl40	18100	mitochondrial ribosomal protein L40	0.775	37.02	1.45E-21

Official Gene Symbol	Entrez Gene ID	Gene description	Normalized Fold Change LOG2(Cryo-2 / Fresh-1)	FPKM Mean of six samples	p-value of Cryo-2 vs Fresh-1
Slc25a22	68267	solute carrier family 25 (mitochondrial carrier, glutamate), member 22	0.774	1.56	3.91E-04
AA465934	613254	expressed sequence AA465934	0.774	1.27	1.37E-03
Zc3hc1	232679	zinc finger, C3HC type 1	0.774	7.30	1.26E-08
Snord118	100216530	small nucleolar RNA, C/D box 118	0.773	114.04	9.72E-11
Bok	51800	BCL2-related ovarian killer	0.773	5.26	7.81E-06
LOC105247207	105247207	-	0.771	9.38	1.27E-06
Tnk2	51789	tyrosine kinase, non-receptor, 2	0.770	0.88	1.99E-04
Dzip3	224170	DAZ interacting protein 3, zinc finger	0.770	1.38	3.40E-07
Phf11d	219132	PHD finger protein 11D	0.770	3.93	1.37E-08
Rpl13	270106	ribosomal protein L13	0.768	364.98	8.20E-68
Crem	12916	cAMP responsive element modulator	0.767	1.27	1.83E-08
Chrac1	93696	chromatin accessibility complex 1	0.765	4.37	1.85E-03
Gas5	14455	growth arrest specific 5	0.765	84.59	5.35E-55
Ppp2r5e	26932	protein phosphatase 2, regulatory subunit B', epsilon	0.765	6.30	1.38E-24
Id2	15902	inhibitor of DNA binding 2	0.764	90.61	2.54E-50
Bbx	70508	bobby sox homolog (Drosophila)	0.763	4.19	1.79E-23
Coq4	227683	coenzyme Q4 homolog (yeast)	0.762	1.30	2.08E-04
Tmem179b	67706	transmembrane protein 179B	0.762	7.17	2.06E-04
Dnlz	52838	DNL-type zinc finger	0.760	1.32	2.21E-03
LOC102637839	102637839	-	0.759	6.60	3.61E-12
Plekhg2	101497	pleckstrin homology domain containing, family G (with RhoGef domain) member 2	0.759	0.97	1.00E-04
Rpl38-ps2	664868	ribosomal protein L38, pseudogene 2	0.759	57.24	1.07E-12
Zcchc11	230594	zinc finger, CCHC domain containing 11	0.759	6.33	3.63E-23
Hist2h4	97122	histone cluster 2, H4	0.757	18.34	1.29E-05
Atad2b	320817	ATPase family, AAA domain containing 2B	0.756	12.57	7.20E-52
Grhpr	76238	glyoxylate reductase/hydroxypyruvate reductase	0.756	5.11	4.41E-05
Tle3	21887	transducin-like enhancer of split 3, homolog of Drosophila E(spl)	0.755	6.57	4.61E-21
Mknk1	17346	MAP kinase-interacting serine/threonine kinase 1	0.755	1.36	3.12E-04
Oraov1	72284	oral cancer overexpressed 1	0.755	1.61	2.08E-05
Panx1	55991	pannexin 1	0.755	2.84	1.49E-04
Qser1	99003	glutamine and serine rich 1	0.754	2.50	8.47E-14
Calcoco1	67488	calcium binding and coiled coil domain 1	0.754	4.21	1.75E-08
Fam213a	70564	family with sequence similarity 213, member A	0.754	6.78	7.75E-10
Crtc3	70461	CREB regulated transcription coactivator 3	0.754	0.55	2.06E-02
Bet1l	54399	blocked early in transport 1 homolog (S. cerevisiae)-like	0.754	3.27	2.63E-04
Zfp346	26919	zinc finger protein 346	0.753	0.88	5.70E-03
Rfxank	19727	regulatory factor X-associated ankyrin-containing protein	0.752	1.34	1.01E-03
Maml1	103806	mastermind like 1 (Drosophila)	0.752	1.57	7.30E-06
Hmga1	15361	high mobility group AT-hook 1	0.752	8.22	3.72E-08
Tbc1d12	209478	TBC1D12: TBC1 domain family, member 12	0.750	0.70	4.70E-03
Pex16	18633	peroxisomal biogenesis factor 16	0.748	1.26	2.28E-02
Cox20	66359	COX20 Cox2 chaperone	0.748	15.53	7.45E-05
Ryr2	20191	ryanodine receptor 2, cardiac	0.745	0.64	7.09E-08
LOC105244944	105244944	-	0.744	0.65	3.55E-13
Cttnbp2nl	80281	CTTNBP2 N-terminal like	0.744	1.63	4.03E-06
Zbtb8a	73680	zinc finger and BTB domain containing 8a	0.742	1.69	1.12E-02

Official Gene Symbol	Entrez Gene ID	Gene description	Normalized Fold Change LOG2(Cryo-2 / Fresh-1)	FPKM Mean of six samples	p-value of Cryo-2 vs Fresh-1
Znrd1	66136	zinc ribbon domain containing, 1	0.741	14.12	2.07E-06
Wdr53	68980	WD repeat domain 53	0.741	2.81	7.94E-04
Cyb5r3	109754	cytochrome b5 reductase 3	0.741	15.78	3.67E-20
Tnfrsf9	21942	tumor necrosis factor receptor superfamily, member 9	0.740	1.22	1.03E-02
Pde8a	18584	phosphodiesterase 8A	0.740	3.48	3.73E-06
Zfp384	269800	zinc finger protein 384	0.739	6.51	3.16E-14
Naf1	234344	nuclear assembly factor 1 homolog (S. cerevisiae)	0.739	4.79	1.75E-10
Ptplb	70757	protein tyrosine phosphatase-like (proline instead of catalytic arginine), member b	0.738	6.20	6.14E-15
Pip5k1b	18719	phosphatidylinositol-4-phosphate 5-kinase, type 1 beta	0.737	1.09	1.29E-02
Rpgrip1l	244585	Rpgrip1-like	0.737	0.55	1.57E-03
Rspry1	67610	ring finger and SPRY domain containing 1	0.737	1.71	6.48E-05
Atp2a3	53313	ATPase, Ca++ transporting, ubiquitous	0.737	0.78	3.03E-03
Nupl2	231042	nucleoporin like 2	0.733	2.80	3.39E-06
Tmem181a	77106	transmembrane protein 181A	0.733	2.01	8.63E-07
Gprc5c	70355	G protein-coupled receptor, family C, group 5, member C	0.731	3.05	5.74E-10
Arhgdig	14570	Rho GDP dissociation inhibitor (GDI) gamma	0.731	4.67	2.92E-03
Sde2	208768	SDE2 telomere maintenance homolog (S. pombe)	0.731	8.42	1.94E-15
Slc35f2	72022	solute carrier family 35, member F2	0.730	10.96	8.43E-20
Pdcd4	18569	programmed cell death 4	0.730	10.36	8.99E-18
Dapp1	26377	dual adaptor for phosphotyrosine and 3-phosphoinositides 1	0.730	0.84	1.45E-02
Lsm2	27756	LSM2 homolog, U6 small nuclear RNA associated (S. cerevisiae)	0.729	22.49	3.61E-15
Daxx	13163	Fas death domain-associated protein	0.729	4.84	3.32E-09
Pex26	74043	peroxisomal biogenesis factor 26	0.729	1.39	9.96E-05
Fra10ac1	70567	FRA10AC1 homolog (human)	0.728	8.53	2.50E-09
Dnajb14	70604	DnaJ (Hsp40) homolog, subfamily B, member 14	0.728	0.79	1.88E-05
Prpf4b	19134	PRP4 pre-mRNA processing factor 4 homolog B (yeast)	0.728	15.65	6.09E-35
Pask	269224	PAS domain containing serine/threonine kinase	0.727	1.31	7.02E-05
Cacnb3	12297	calcium channel, voltage-dependent, beta 3 subunit	0.726	2.64	3.08E-07
Lancl1	14768	LanC (bacterial lantibiotic synthetase component C)-like 1	0.725	1.24	4.20E-04
Rassf1	56289	Ras association (RalGDS/AF-6) domain family member 1	0.724	3.65	3.34E-05
Ndufa6	67130	NADH dehydrogenase (ubiquinone) 1 alpha subcomplex, 6 (B14)	0.723	70.03	8.74E-21
LOC105244834	105244834	-	0.722	60.78	3.78E-32
Mcts2	66405	malignant T cell amplified sequence 2	0.722	7.87	1.59E-04
Tmem147	69804	transmembrane protein 147	0.721	18.49	2.77E-08
Zfp182	319535	zinc finger protein 182	0.721	1.01	1.82E-04
Mex3b	108797	mex3 homolog B (C. elegans)	0.720	1.29	1.26E-03
Mzt1	76789	mitotic spindle organizing protein 1	0.720	9.70	1.24E-10
Myo1d	338367	myosin ID	0.718	1.23	4.99E-06
Magea8	17144	melanoma antigen, family A, 8	0.716	2.61	2.79E-03
Smdt1	69029	single-pass membrane protein with aspartate rich tail 1	0.716	20.38	5.29E-07
Tmem38b	52076	transmembrane protein 38B	0.714	3.73	8.31E-07

Official Gene Symbol	Entrez Gene ID	Gene description	Normalized Fold Change LOG2(Cryo-2 / Fresh-1)	FPKM Mean of six samples	p-value of Cryo-2 vs Fresh-1
Dcbld2	73379	discoidin, CUB and LCCL domain containing 2	0.713	1.35	8.64E-06
Hpdl	242642	4-hydroxyphenylpyruvate dioxygenase-like	0.713	7.73	6.21E-07
Hmgxb3	106894	HMG box domain containing 3	0.712	2.86	1.80E-09
Uvssa	71101	UV stimulated scaffold protein A	0.711	0.94	1.48E-04
Senp6	215351	SUMO/sentrin specific peptidase 6	0.711	14.50	2.01E-33
Praf2	54637	PRA1 domain family 2	0.711	7.05	2.17E-05
Snx12	55988	sorting nexin 12	0.710	3.76	1.04E-08
Snhg4	100503380	small nucleolar RNA host gene 4	0.710	9.37	4.43E-20
Litaf	56722	LPS-induced TN factor	0.710	15.52	5.27E-19
Mrps24	64660	mitochondrial ribosomal protein S24	0.709	23.75	8.17E-14
Cox8a	12868	cytochrome c oxidase subunit VIIIa	0.709	67.43	1.98E-19
Slc15a2	57738	solute carrier family 15 (H+/peptide transporter), member 2	0.708	3.22	1.57E-08
Rsbn1	229675	rosbin, round spermatid basic protein 1	0.708	2.93	6.20E-11
Akap17b	338351	A kinase (PRKA) anchor protein 17B	0.708	1.12	1.21E-04
Ttl	69737	tubulin tyrosine ligase	0.707	2.54	2.28E-07
Bcr	110279	breakpoint cluster region	0.706	2.20	1.01E-09
Plod1	18822	procollagen-lysine, 2-oxoglutarate 5-dioxygenase 1	0.705	4.01	4.43E-08
Mpp1	17524	membrane protein, palmitoylated	0.703	11.86	3.80E-14
Cnpy3	72029	canopy 3 homolog (zebrafish)	0.702	1.69	1.47E-02
Xpr1	19775	xenotropic and polytropic retrovirus receptor 1	0.702	5.58	4.45E-19
LOC102634296	102634296	-	0.700	2.97	2.58E-04
Zrsr2	22184	zinc finger (CCCH type), RNA binding motif and serine/arginine rich 2	0.700	4.09	4.06E-16
Uhmk1	16589	U2AF homology motif (UHM) kinase 1	0.698	6.82	1.71E-09
Klf3	16599	Kruppel-like factor 3 (basic)	0.697	1.04	1.03E-03
Myg1	60315	melanocyte proliferating gene 1	0.697	7.09	1.63E-06
Adk	11534	adenosine kinase	0.697	4.87	3.02E-06
Tmem2	83921	transmembrane protein 2	0.697	0.57	4.97E-03
Zbtb26	320633	zinc finger and BTB domain containing 26	0.697	1.22	1.10E-03
Srp14	20813	signal recognition particle 14	0.696	51.70	1.30E-18
Ypel3	66090	yippee-like 3 (Drosophila)	0.695	2.41	2.08E-02
Ifi30	65972	interferon gamma inducible protein 30	0.695	25.13	2.50E-13
Ap1m2	11768	adaptor protein complex AP-1, mu 2 subunit	0.694	10.37	1.89E-14
Chpf2	100910	chondroitin polymerizing factor 2	0.694	0.53	2.84E-02
Abhd17a	216169	abhydrolase domain containing 17A	0.692	9.68	5.51E-07
Dmd	13405	dystrophin, muscular dystrophy	0.691	0.70	1.23E-07
Zfp869	66869	zinc finger protein 869	0.691	3.38	2.52E-08
Slc9a3r1	26941	solute carrier family 9 (sodium/hydrogen exchanger), member 3 regulator 1	0.691	13.98	5.94E-15
Runx1	12394	runt related transcription factor 1	0.690	1.32	5.74E-06
Tmem37	170706	transmembrane protein 37	0.690	5.22	1.15E-03
Zfp553	233887	zinc finger protein 553	0.689	3.90	8.01E-07
Cdkn2aip	70925	CDKN2A interacting protein	0.689	3.64	1.11E-08
Tmem102	380705	transmembrane protein 102	0.686	3.13	2.80E-04
Cdkal1	68916	CDK5 regulatory subunit associated protein 1-like 1	0.686	1.93	2.15E-08
Eci2	23986	enoyl-Coenzyme A delta isomerase 2	0.686	6.75	3.41E-06
Rab43	69834	RAB43, member RAS oncogene family	0.685	0.63	1.29E-02
Fam161b	217705	family with sequence similarity 161, member B	0.685	0.65	3.50E-02
Hist3h2ba	78303	histone cluster 3, H2ba	0.684	5.29	3.12E-02
Hist1h3b	319150	histone cluster 1, H3b	0.684	66.30	6.47E-16
Ccdc53	67282	coiled-coil domain containing 53	0.684	9.40	2.31E-09

Official Gene Symbol	Entrez Gene ID	Gene description	Normalized Fold Change LOG2(Cryo-2 / Fresh-1)	FPKM Mean of six samples	p-value of Cryo-2 vs Fresh-1
Xylb	102448	xylulokinase homolog (H. influenzae)	0.683	1.39	2.43E-04
Tmem230	70612	transmembrane protein 230	0.682	3.20	7.15E-05
Tmsb4x	19241	thymosin, beta 4, X chromosome	0.682	24.49	3.13E-10
Brf1	72308	BRF1 homolog, subunit of RNA polymerase III transcription initiation factor IIIB (S. cerevisiae)	0.681	0.71	6.54E-04
Rnf169	108937	ring finger protein 169	0.680	0.95	3.74E-06
Vav2	22325	vav 2 oncogene	0.680	5.62	2.30E-13
Fam46c	74645	family with sequence similarity 46, member C	0.680	1.74	6.48E-06
Ube2a	22209	ubiquitin-conjugating enzyme E2A	0.679	14.62	1.62E-12
Rnf38	73469	ring finger protein 38	0.679	6.17	3.37E-16
Ada	11486	adenosine deaminase	0.679	1.24	4.21E-02
Cnpy2	56530	canopy 2 homolog (zebrafish)	0.679	9.11	1.06E-04
Dennd1a	227801	DENN/MADD domain containing 1A	0.678	1.66	8.69E-05
Cryab	12955	crystallin, alpha B	0.678	7.89	2.12E-06
Zfp526	210172	zinc finger protein 526	0.677	0.62	2.03E-02
Xkrx	331524	X Kell blood group precursor related X linked	0.677	0.68	9.34E-03
Cpeb4	67579	cytoplasmic polyadenylation element binding protein 4	0.675	2.60	4.18E-09
Lin37	75660	lin-37 homolog (C. elegans)	0.675	10.66	1.20E-07
Hist1h2ab	319172	histone cluster 1, H2ab	0.675	81.93	5.55E-04
Prickle1	106042	prickle homolog 1 (Drosophila)	0.673	0.84	5.39E-05
Foxred1	235169	FAD-dependent oxidoreductase domain containing 1	0.672	3.13	1.19E-04
Gabpb1	14391	GA repeat binding protein, beta 1	0.671	3.33	1.02E-08
Hist1h2af	319173	histone cluster 1, H2af	0.670	48.44	2.30E-03
Klhl2_2	77113	kelch-like 2, Mayven	0.670	5.83	1.27E-02
Cog5	238123	component of oligomeric golgi complex 5	0.669	1.64	2.39E-06
Ift88	21821	intraflagellar transport 88	0.669	2.10	2.09E-05
Ube2d1	216080	ubiquitin-conjugating enzyme E2D 1	0.668	5.99	5.32E-04
Ormdl2	66844	ORM1-like 2 (S. cerevisiae)	0.668	7.86	1.73E-08
Bcl2l13	94044	BCL2-like 13 (apoptosis facilitator)	0.668	3.62	6.55E-13
Ice2	93697	interactor of little elongation complex ELL subunit 2	0.667	2.46	3.95E-06
Ovol1	18426	OVO homolog-like 1 (Drosophila)	0.667	0.61	3.82E-02
Nle1	217011	notchless homolog 1 (Drosophila)	0.667	2.42	1.87E-03
Asz1	74068	ankyrin repeat, SAM and basic leucine zipper domain containing 1	0.667	5.42	6.20E-06
Wipf1	215280	WAS/WASL interacting protein family, member 1	0.667	3.55	2.00E-10
Mrpl4	66163	mitochondrial ribosomal protein L4	0.667	13.95	8.81E-10
Mef2d	17261	myocyte enhancer factor 2D	0.667	3.73	3.44E-10
Pnrc1	108767	proline-rich nuclear receptor coactivator 1	0.666	2.50	3.10E-03
Snrnp27	66618	small nuclear ribonucleoprotein 27 (U4/U6.U5)	0.666	27.81	3.53E-12
Pqlc2	212555	PQ loop repeat containing 2	0.666	0.97	4.29E-04
Gemin7	69731	gem (nuclear organelle) associated protein 7	0.665	11.16	2.36E-05
Tmem9	66241	transmembrane protein 9	0.665	25.03	8.64E-17
Susd6	217684	sushi domain containing 6	0.665	4.32	2.68E-11
Adipor2	68465	adiponectin receptor 2	0.664	2.98	7.07E-07
Ier2	15936	immediate early response 2	0.664	5.30	1.36E-04
Slc38a7	234595	solute carrier family 38, member 7	0.664	4.87	1.82E-09
Zswim5	74464	zinc finger SWIM-type containing 5	0.663	0.62	2.73E-02
Lzic	69151	leucine zipper and CTNNBIP1 domain containing	0.662	5.87	1.19E-06
Mdh1	17449	malate dehydrogenase 1, NAD (soluble)	0.662	15.47	1.33E-12
Dock5	68813	dedicator of cytokinesis 5	0.662	1.44	3.86E-08
Eif3g	53356	eukaryotic translation initiation factor 3, subunit G	0.661	49.14	8.31E-22

Official Gene Symbol	Entrez Gene ID	Gene description	Normalized Fold Change LOG2(Cryo-2 / Fresh-1)	FPKM Mean of six samples	p-value of Cryo-2 vs Fresh-1
Ston1	77057	stonin 1	0.661	1.07	1.57E-02
Lonrf3	74365	LON peptidase N-terminal domain and ring finger 3	0.661	1.51	1.34E-05
Ylpm1	56531	YLP motif containing 1	0.661	3.07	1.16E-16
Kbtbd4	67136	kelch repeat and BTB (POZ) domain containing 4	0.659	2.37	2.04E-03
Thumpd2	72167	THUMP domain containing 2	0.658	0.64	4.55E-02
Fzr1	56371	fizzy/cell division cycle 20 related 1 (Drosophila)	0.658	5.25	8.70E-07
Dusp14	56405	dual specificity phosphatase 14	0.658	18.36	5.14E-19
Lonp2	66887	lon peptidase 2, peroxisomal	0.657	8.43	1.46E-12
Atpif1	11983	ATPase inhibitory factor 1	0.656	24.89	1.99E-14
Sycn	68416	syncollin	0.656	8.90	2.22E-03
Zfp777	72306	zinc finger protein 777	0.656	1.62	8.39E-06
Sdhaf2	66072	succinate dehydrogenase complex assembly factor 2	0.656	5.85	5.42E-09
Myl12a	67268	myosin, light chain 12A, regulatory, non-sarcomeric	0.656	42.34	9.21E-28
Nkap	67050	NFKB activating protein	0.655	8.06	6.81E-14
Cables2	252966	CDK5 and Abl enzyme substrate 2	0.654	2.04	3.23E-04
Ajuba	16475	ajuba LIM protein	0.654	3.45	1.09E-06
Pagr1a	67278	PAXIP1 associated glutamate rich protein 1A	0.654	6.97	2.53E-05
Card6	239319	caspase recruitment domain family, member 6	0.654	0.69	9.40E-03
Asf1a	66403	anti-silencing function 1A histone chaperone	0.653	14.97	6.67E-14
Vps26b	69091	vacuolar protein sorting 26 homolog B (yeast)	0.653	2.48	1.55E-08
Tyw5	68736	tRNA-yW synthesizing protein 5	0.653	3.53	9.17E-05
Lrch3	70144	leucine-rich repeats and calponin homology (CH) domain containing 3	0.653	1.47	6.35E-07
Ahsa2	268390	AHA1, activator of heat shock protein ATPase 2	0.652	2.98	2.31E-06
Zdhhc21	68268	zinc finger, DHHC domain containing 21	0.651	3.03	6.40E-13
Btg1	12226	B cell translocation gene 1, anti-proliferative	0.651	6.31	2.59E-11
Usp33	170822	ubiquitin specific peptidase 33	0.650	16.01	4.39E-25
Pced1b	239647	PC-esterase domain containing 1B	0.650	2.68	1.61E-09
Pag1	94212	phosphoprotein associated with glycosphingolipid microdomains 1	0.650	0.93	1.72E-04
Rps6kc1	320119	ribosomal protein S6 kinase polypeptide 1	0.650	0.65	3.84E-02
Upf3b	68134	UPF3 regulator of nonsense transcripts homolog B (yeast)	0.650	13.56	4.10E-15
Cyr61	16007	cysteine rich protein 61	0.650	4.82	1.89E-05
Rhno1	72440	RAD9-HUS1-RAD1 interacting nuclear orphan 1	0.649	1.84	4.10E-06
Ndufa11	69875	NADH dehydrogenase (ubiquinone) 1 alpha subcomplex 11	0.648	11.34	2.48E-13
Fgfr1	14182	fibroblast growth factor receptor 1	0.648	4.64	3.35E-12
Arid1a	93760	AT rich interactive domain 1A (SWI-like)	0.647	13.27	3.66E-34
Vmn2r-ps100	100041855	vomeronasal 2, receptor, pseudogene 100	0.646	1.41	4.15E-04
Pih1d1	68845	PIH1 domain containing 1	0.645	3.86	4.36E-04
Setd1a	233904	SET domain containing 1A	0.645	4.99	1.30E-12
Snrpc	20630	U1 small nuclear ribonucleoprotein C	0.645	30.41	1.17E-10
LOC105246960	105246960	-	0.645	1.12	3.39E-02
LOC102638745	102638745	-	0.644	6.42	7.92E-05
Rdm1	66599	RAD52 motif 1	0.644	272.48	5.13E-11
Myh14	71960	myosin, heavy polypeptide 14	0.643	0.65	4.51E-03
Ptch1	19206	patched homolog 1	0.642	0.79	9.07E-05
Rcor1	217864	REST corepressor 1	0.642	10.12	1.37E-25
Apopt1	68020	apoptogenic, mitochondrial 1	0.642	2.16	1.41E-02
Slc35e3	215436	solute carrier family 35, member E3	0.642	2.05	4.24E-04

Official Gene Symbol	Entrez Gene ID	Gene description	Normalized Fold Change LOG2(Cryo-2 / Fresh-1)	FPKM Mean of six samples	p-value of Cryo-2 vs Fresh-1
Tdh	58865	L-threonine dehydrogenase	0.641	2.14	8.04E-03
Gjb5	14622	gap junction protein, beta 5	0.641	5.69	2.39E-05
Siva1	30954	SIVA1, apoptosis-inducing factor	0.639	22.31	5.97E-08
Il6ra	16194	interleukin 6 receptor, alpha	0.639	1.08	3.84E-03
Slc7a5	20539	solute carrier family 7 (cationic amino acid transporter, y+ system), member 5	0.639	5.00	4.04E-08
Serinc1	56442	serine incorporator 1	0.638	17.80	3.61E-18
Ube3b	117146	ubiquitin protein ligase E3B	0.638	2.98	3.43E-07
Dpy30	66310	dpy-30 homolog (C. elegans)	0.638	49.04	8.20E-19
Gpt2	108682	glutamic pyruvate transaminase (alanine aminotransferase) 2	0.638	1.03	1.55E-03
Ndufaf2	75597	NADH dehydrogenase (ubiquinone) 1 alpha subcomplex, assembly factor 2	0.637	19.76	6.70E-08
Mospd3	68929	motile sperm domain containing 3	0.637	1.76	1.77E-02
Mycn	18109	v-myc myelocytomatosis viral related oncogene, neuroblastoma derived (avian)	0.636	1.09	2.61E-02
Foxk2	68837	forkhead box K2	0.636	2.23	7.74E-06
Skida1	72668	SKI/DACH domain containing 1	0.636	1.29	2.21E-04
B4galt1	14595	UDP-Gal:betaGlcNAc beta 1,4-galactosyltransferase, polypeptide 1	0.635	6.18	1.93E-13
Bambi	68010	BMP and activin membrane-bound inhibitor	0.635	0.65	1.97E-02
Cbll1	104836	Casitas B-lineage lymphoma-like 1	0.635	3.41	6.30E-06
Pik3r3	18710	phosphatidylinositol 3 kinase, regulatory subunit, polypeptide 3 (p55)	0.634	0.92	3.06E-03
Mrpl45	67036	mitochondrial ribosomal protein L45	0.633	19.72	2.55E-13
Pla2g4c	232889	phospholipase A2, group IVC (cytosolic, calcium-independent)	0.632	0.53	2.36E-02
Atp5sl	66349	ATP5S-like	0.632	2.22	1.75E-03
Tma7	66167	translational machinery associated 7 homolog (S. cerevisiae)	0.631	112.40	4.99E-23
Zfp770	228491	zinc finger protein 770	0.630	1.34	1.15E-03
Efhb	211482	EF hand domain family, member B	0.630	0.85	2.40E-02
Zfp68	24135	zinc finger protein 68	0.630	2.18	1.73E-05
Lnpep	240028	leucyl/cystinyl aminopeptidase	0.630	4.96	1.50E-09
Baiap2l1	66898	BAI1-associated protein 2-like 1	0.629	6.31	1.16E-09
Arhgap32	330914	Rho GTPase activating protein 32	0.629	2.14	2.20E-13
Asap2	211914	ArfGAP with SH3 domain, ankyrin repeat and PH domain 2	0.629	3.11	7.08E-09
Hist1h2ao	665433	histone cluster 1, H2ao	0.629	264.56	3.55E-19
Ndufa2	17991	NADH dehydrogenase (ubiquinone) 1 alpha subcomplex, 2	0.629	24.14	6.51E-07
Smurf1	75788	SMAD specific E3 ubiquitin protein ligase 1	0.629	1.44	2.16E-04
Cdk2	12566	cyclin-dependent kinase 2	0.628	11.01	2.06E-09
LOC105244398	105244398	-	0.628	0.54	3.26E-04
Zfp763	73451	zinc finger protein 763	0.628	2.83	4.26E-05
Ttc39b	69863	tetratricopeptide repeat domain 39B	0.627	3.13	4.58E-11
Cd74	16149	CD74 antigen (invariant polypeptide of major histocompatibility complex, class II antigen-associated)	0.627	3.03	7.10E-03
Zfp738	408068	zinc finger protein 738	0.627	2.69	8.95E-06
Tsc22d2	72033	TSC22 domain family, member 2	0.626	1.73	3.20E-07
Btg3	12228	B cell translocation gene 3	0.625	5.02	3.55E-04

Official Gene Symbol	Entrez Gene ID	Gene description	Normalized Fold Change LOG2(Cryo-2 / Fresh-1)	FPKM Mean of six samples	p-value of Cryo-2 vs Fresh-1
Wnk3	279561	WNK lysine deficient protein kinase 3	0.624	2.87	7.67E-15
Gipc1	67903	GIPC PDZ domain containing family, member 1	0.624	4.99	2.06E-04
Cdk18	18557	cyclin-dependent kinase 18	0.623	2.06	9.29E-04
Snhg6	73824	small nucleolar RNA host gene 6	0.622	11.66	3.16E-10
AK129341	234915	cDNA sequence AK129341	0.622	0.78	2.45E-04
Paxbp1	67367	PAX3 and PAX7 binding protein 1	0.621	5.11	1.06E-08
LOC105245116	105245116	-	0.620	0.63	6.31E-04
Bloc1s4	117197	biogenesis of lysosomal organelles complex-1, subunit 4, cappuccino	0.620	3.92	4.16E-03
Alkbh1	211064	alkB, alkylation repair homolog 1 (E. coli)	0.620	5.36	1.31E-04
Bnip2	12175	BCL2/adenovirus E1B interacting protein 2	0.620	1.94	2.95E-05
Dppa1	347708	developmental pluripotency associated 1	0.619	23.44	3.33E-21
Sh3kbp1	58194	SH3-domain kinase binding protein 1	0.619	0.51	2.02E-02
Gdi1	14567	guanosine diphosphate (GDP) dissociation inhibitor 1	0.619	4.30	1.21E-05
AU019823	270156	expressed sequence AU019823	0.618	2.40	4.72E-05
Sgce	20392	sarcoglycan, epsilon	0.618	1.23	3.10E-02
Cope	59042	coatomer protein complex, subunit epsilon	0.618	25.13	7.00E-10
Arv1	68865	ARV1 homolog (yeast)	0.618	0.72	2.58E-03
Bcat2	12036	branched chain aminotransferase 2, mitochondrial	0.617	3.20	2.52E-04
Usp53	99526	ubiquitin specific peptidase 53	0.616	3.91	4.35E-12
Hmgcl	15356	3-hydroxy-3-methylglutaryl-Coenzyme A lyase	0.616	5.97	1.42E-06
Patz1	56218	POZ (BTB) and AT hook containing zinc finger 1	0.616	1.51	4.38E-03
Dpp4	13482	dipeptidylpeptidase 4	0.615	0.88	5.38E-04
Uqcc2	67267	ubiquinol-cytochrome c reductase complex assembly factor 2	0.615	8.26	1.23E-03
Gosr1	53334	golgi SNAP receptor complex member 1	0.614	4.31	4.14E-08
Sart1	20227	squamous cell carcinoma antigen recognized by T cells 1	0.614	4.83	3.91E-09
Nfkb2	18034	nuclear factor of kappa light polypeptide gene enhancer in B cells 2, p49/p100	0.614	1.17	2.93E-03
Rps28	54127	ribosomal protein S28	0.612	200.15	6.23E-22
Zfp212	232784	Zinc finger protein 212	0.612	2.59	7.67E-04
Tbc1d20	67231	TBC1 domain family, member 20	0.612	4.09	3.41E-04
Pon2	330260	paraoxonase 2	0.611	4.31	1.64E-04
Mea1	17256	male enhanced antigen 1	0.611	10.64	2.10E-06
Wnt3a	22416	wingless-type MMTV integration site family, member 3A	0.611	2.45	1.46E-03
Tfap2c	21420	transcription factor AP-2, gamma	0.610	11.92	2.02E-15
Dnttip1	76233	deoxynucleotidyltransferase, terminal, interacting protein 1	0.610	4.92	1.68E-03
Snx16	74718	sorting nexin 16	0.610	8.65	7.98E-09
Bloc1s1	14533	biogenesis of lysosomal organelles complex-1, subunit 1	0.610	11.47	7.14E-04
Ing5	66262	inhibitor of growth family, member 5	0.610	2.93	5.39E-08
Trim38	214158	tripartite motif-containing 38	0.609	3.69	3.23E-04
Ndufaf1	69702	NADH dehydrogenase (ubiquinone) 1 alpha subcomplex, assembly factor 1	0.608	4.35	6.53E-04
Sox2	20674	SRY (sex determining region Y)-box 2	0.608	9.78	1.79E-09
Ppp1r37	232947	protein phosphatase 1, regulatory subunit 37	0.608	2.79	3.36E-05
Slc9a6	236794	solute carrier family 9 (sodium/hydrogen exchanger), member 6	0.607	2.90	2.02E-05

Official Gene Symbol	Entrez Gene ID	Gene description	Normalized Fold Change LOG2(Cryo-2 / Fresh-1)	FPKM Mean of six samples	p-value of Cryo-2 vs Fresh-1
Mrpl21	353242	mitochondrial ribosomal protein L21	0.607	20.89	2.52E-14
Sema5b	20357	sema domain, seven thrombospondin repeats (type 1 and type 1-like), transmembrane domain (TM) and short cytoplasmic domain, (semaphorin) 5B	0.607	0.84	1.18E-02
Klhl20	226541	kelch-like 20	0.607	2.41	7.15E-05
Ubqln2	54609	ubiquilin 2	0.607	14.22	3.17E-16
Pole3	59001	polymerase (DNA directed), epsilon 3 (p17 subunit)	0.606	24.83	1.20E-05
Manea	242362	mannosidase, endo-alpha	0.605	0.85	1.05E-02
Szt2	230676	seizure threshold 2	0.605	0.91	4.90E-05
Wbp5	22381	WW domain binding protein 5	0.605	4.13	1.27E-02
C87436	232196	expressed sequence C87436	0.604	0.64	4.63E-02
Rltpr	234695	RGD motif, leucine rich repeats, tropomodulin domain and proline-rich containing	0.604	0.57	4.80E-02
Trmt44	78890	tRNA methyltransferase 44	0.604	1.79	9.64E-04
Stac2	217154	SH3 and cysteine rich domain 2	0.603	2.83	1.55E-04
Yrdc	230734	yrdC domain containing (E.coli)	0.602	4.88	6.01E-04
Fancb	237211	Fanconi anemia, complementation group B	0.602	1.98	1.69E-03
Zbtb10	229055	zinc finger and BTB domain containing 10	0.602	18.50	1.63E-25
Entpd7	93685	ectonucleoside triphosphate diphosphohydrolase 7	0.602	1.36	3.04E-04
Scarb1	20778	scavenger receptor class B, member 1	0.602	2.19	6.61E-04
Ccny	67974	cyclin Y	0.601	3.42	1.40E-08
Mul1	68350	mitochondrial ubiquitin ligase activator of NFKB 1	0.600	1.71	2.15E-03
Mettl18	69962	methyltransferase like 18	0.599	3.18	7.54E-03
Nrip1	268903	nuclear receptor interacting protein 1	0.599	1.58	2.00E-09
Crebbp	12914	CREB binding protein	0.598	12.27	4.49E-32
Tfdp2	211586	transcription factor Dp 2	0.598	1.93	5.76E-07
Fermt2	218952	fermitin family homolog 2 (Drosophila)	0.598	11.65	1.10E-13
Bag3	29810	BCL2-associated athanogene 3	0.597	18.79	1.02E-14
Fbxo33	70611	F-box protein 33	0.597	4.65	3.14E-07
Klhl26	234378	kelch-like 26	0.597	0.80	4.36E-02
Ufm1	67890	ubiquitin-fold modifier 1	0.596	7.55	1.88E-10
Ndufb4	68194	NADH dehydrogenase (ubiquinone) 1 beta subcomplex 4	0.595	30.03	1.42E-05
Fyttd1	69823	forty-two-three domain containing 1	0.595	6.56	1.62E-09
Lrrc75a	192976	leucine rich repeat containing 75A	0.595	0.97	4.39E-02
Txndc17	52700	thioredoxin domain containing 17	0.595	15.03	9.49E-08
Fkbp11	66120	FK506 binding protein 11	0.595	5.33	4.44E-03
Ndufa7	66416	NADH dehydrogenase (ubiquinone) 1 alpha subcomplex, 7 (B14.5a)	0.595	45.63	3.15E-09
Arid3b	56380	AT rich interactive domain 3B (BRIGHT-like)	0.595	1.63	1.31E-03
Traf4	22032	TNF receptor associated factor 4	0.594	8.42	2.39E-10
Tbc1d24	224617	TBC1 domain family, member 24	0.594	0.64	3.83E-03
Rpl17-ps3	100040745	ribosomal protein L17, pseudogene 3	0.594	19.03	4.04E-04
Gls	14660	glutaminase	0.593	5.85	3.72E-22
Blvrb	233016	biliverdin reductase B (flavin reductase (NADPH))	0.593	7.13	1.14E-03
Mad2l2	71890	MAD2 mitotic arrest deficient-like 2	0.592	3.05	4.31E-03
Ccdc130	67736	coiled-coil domain containing 130	0.591	1.90	3.29E-03
ND2	17717	URF2	0.591	######	8.84E-57
Prss35	244954	protease, serine 35	0.591	0.77	3.73E-02
Wdr62	233064	WD repeat domain 62	0.590	1.89	1.99E-04
Smg9	71997	smg-9 homolog, nonsense mediated mRNA decay factor (C. elegans)	0.590	1.67	6.67E-03

Official Gene Symbol	Entrez Gene ID	Gene description	Normalized Fold Change LOG2(Cryo-2 / Fresh-1)	FPKM Mean of six samples	p-value of Cryo-2 vs Fresh-1
Nlrp4f	97895	NLR family, pyrin domain containing 4F	0.590	2.23	8.10E-04
Sp3os	100503849	trans-acting transcription factor 3, opposite strand	0.589	0.52	4.97E-03
Adtrp	109254	androgen dependent TFPI regulating protein	0.589	1.39	1.71E-02
Nt5c3	107569	5'-nucleotidase, cytosolic III	0.589	4.28	6.35E-04
Gusb	110006	glucuronidase, beta	0.589	2.28	6.07E-03
Ftl1	14325	ferritin light chain 1	0.589	250.78	2.97E-30
Gpatch1	67471	G patch domain containing 1	0.589	4.39	5.61E-06
Zfp369	170936	zinc finger protein 369	0.588	0.72	2.42E-02
Cr1l	12946	complement component (3b/4b) receptor 1-like	0.587	3.51	4.58E-03
Rft1	328370	RFT1 homolog (S. cerevisiae)	0.587	2.04	7.91E-03
Nme1	18102	NME/NM23 nucleoside diphosphate kinase 1	0.586	50.74	6.30E-16
Rdh10	98711	retinol dehydrogenase 10 (all-trans)	0.586	3.62	4.19E-04
Mlh3	217716	mutL homolog 3 (E coli)	0.586	1.99	5.48E-05
Aurkaip1	66077	aurora kinase A interacting protein 1	0.586	18.41	9.59E-08
Gpatch11	53951	G patch domain containing 11	0.585	3.25	3.78E-06
Slc10a3-ubl4	100169864	Slc10a3-Ubl4 readthrough	0.585	7.09	9.58E-04
Ngrn	83485	neugrin, neurite outgrowth associated	(0.585)	9.50	2.19E-08
Tctn3	67590	tectonic family member 3	(0.586)	0.98	3.72E-02
Umps	22247	uridine monophosphate synthetase	(0.586)	11.30	1.50E-21
Slc25a30	67554	solute carrier family 25, member 30	(0.586)	0.68	1.36E-02
Fkbp8	14232	FK506 binding protein 8	(0.587)	11.17	1.91E-18
Alg9	102580	asparagine-linked glycosylation 9 (alpha 1,2 mannosyltransferase)	(0.588)	3.34	2.89E-09
Pnp	18950	purine-nucleoside phosphorylase	(0.589)	9.68	8.12E-17
Xrcc1	22594	X-ray repair complementing defective repair in Chinese hamster cells 1	(0.589)	7.39	1.83E-10
Fam57b	68952	family with sequence similarity 57, member B	(0.589)	0.54	3.62E-02
LOC105245545	105245545	-	(0.589)	4.78	3.10E-05
Ptpra	19262	protein tyrosine phosphatase, receptor type, A	(0.590)	9.71	3.57E-20
Gna11	14672	guanine nucleotide binding protein, alpha 11	(0.591)	4.14	1.69E-08
Mon1b	270096	MON1 homolog b (yeast)	(0.592)	2.02	1.56E-06
Mbtps2	270669	membrane-bound transcription factor peptidase, site 2	(0.592)	4.85	4.30E-14
Synj1	104015	synaptojanin 1	(0.593)	2.06	2.41E-11
Clk4	12750	CDC like kinase 4	(0.593)	1.99	1.98E-05
Dync1li2	234663	dynein, cytoplasmic 1 light intermediate chain 2	(0.593)	5.51	7.67E-17
Mapk8	26419	mitogen-activated protein kinase 8	(0.594)	2.63	9.39E-10
Mbnl3	171170	muscleblind-like 3 (Drosophila)	(0.594)	1.97	1.92E-11
Ddx20	53975	DEAD (Asp-Glu-Ala-Asp) box polypeptide 20	(0.594)	13.21	2.82E-23
Ube2b	22210	ubiquitin-conjugating enzyme E2B	(0.594)	12.61	7.19E-16
Trp53inp2	68728	transformation related protein 53 inducible nuclear protein 2	(0.595)	3.26	2.20E-10
Zfp345	545471	zinc finger protein 345	(0.595)	5.34	5.58E-10
Zfp410	52708	zinc finger protein 410	(0.595)	2.20	7.54E-08
Epn3	71889	epsin 3	(0.596)	1.90	2.14E-06
Mapk9	26420	mitogen-activated protein kinase 9	(0.596)	3.28	1.20E-10
Suds3	71954	suppressor of defective silencing 3 homolog (S. cerevisiae)	(0.596)	7.87	4.54E-12
Secisbp2l	70354	SECIS binding protein 2-like	(0.597)	4.03	1.98E-16
Dr1	13486	down-regulator of transcription 1	(0.597)	13.50	8.37E-21
Zfp687	78266	zinc finger protein 687	(0.597)	1.90	3.47E-06
Ccnf	12449	cyclin F	(0.597)	8.83	4.78E-15

Official Gene Symbol	Entrez Gene ID	Gene description	Normalized Fold Change LOG2(Cryo-2 / Fresh-1)	FPKM Mean of six samples	p-value of Cryo-2 vs Fresh-1
Micu1	216001	mitochondrial calcium uptake 1	(0.597)	3.48	6.92E-06
Cul5	75717	cullin 5	(0.598)	69.19	1.81E-74
Dtnb	13528	dystrobrevin, beta	(0.598)	2.05	4.86E-07
Kifc1	100502766	kinesin family member C1	(0.598)	9.71	3.51E-12
Nbeal2	235627	neurobeachin-like 2	(0.598)	0.74	9.87E-05
Eri1	67276	exoribonuclease 1	(0.598)	6.03	7.19E-16
Arf1	11840	ADP-ribosylation factor 1	(0.599)	41.36	1.25E-37
Agpat2	67512	1-acylglycerol-3-phosphate O-acyltransferase 2 (lysophosphatidic acid acyltransferase, beta)	(0.599)	28.00	5.43E-25
Gemin4	276919	gem (nuclear organelle) associated protein 4	(0.599)	4.12	1.26E-09
Mast2	17776	microtubule associated serine/threonine kinase 2	(0.600)	2.27	1.09E-10
Zfp276	57247	zinc finger protein (C2H2 type) 276	(0.601)	0.58	8.41E-03
Noxred1	71275	NADP+ dependent oxidoreductase domain containing 1	(0.601)	0.72	3.42E-02
Cfap36	216618	cilia and flagella associated protein 36	(0.602)	2.65	2.97E-03
Fam114a2	67726	family with sequence similarity 114, member A2	(0.602)	7.03	7.22E-13
Tbc1d25	209815	TBC1 domain family, member 25	(0.602)	1.71	2.21E-03
Zfp39	22698	zinc finger protein 39	(0.602)	1.21	6.83E-04
Ap4e1	108011	adaptor-related protein complex AP-4, epsilon 1	(0.602)	2.03	1.40E-09
Gosr2	56494	golgi SNAP receptor complex member 2	(0.602)	6.22	9.71E-12
Zfp566	72556	zinc finger protein 566	(0.602)	1.48	2.17E-02
Ddx3y	26900	DEAD (Asp-Glu-Ala-Asp) box polypeptide 3, Y-linked	(0.603)	8.68	2.96E-23
Prcp	72461	prolylcarboxypeptidase (angiotensinase C)	(0.603)	1.21	2.09E-03
Rffl	67338	ring finger and FYVE like domain containing protein	(0.604)	3.34	1.41E-09
Add3	27360	adducin 3 (gamma)	(0.604)	3.06	2.94E-09
Fbxo18	50755	F-box protein 18	(0.605)	2.18	1.83E-08
Zfp418	232854	zinc finger protein 418	(0.605)	1.43	2.61E-03
Aasdhppt	67618	aminoadipate-semialdehyde dehydrogenase-phosphopantetheinyl transferase	(0.606)	3.00	4.75E-05
Lpin1	14245	lipin 1	(0.606)	0.82	2.25E-04
Cep89	72140	centrosomal protein 89	(0.606)	1.86	4.63E-04
Nup35	69482	nucleoporin 35	(0.606)	9.26	9.41E-04
Cdc40	71713	cell division cycle 40	(0.607)	6.92	2.94E-14
Gpatch2l	70373	G patch domain containing 2 like	(0.607)	2.78	6.87E-08
Tsc1	64930	tuberous sclerosis 1	(0.607)	3.13	1.84E-13
Tmx3	67988	thioredoxin-related transmembrane protein 3	(0.608)	8.47	1.53E-20
Stt3b	68292	STT3, subunit of the oligosaccharyltransferase complex, homolog B (S. cerevisiae)	(0.608)	18.21	1.25E-36
Fech	14151	ferrochelatase	(0.608)	3.71	7.68E-07
G6pdx	14381	glucose-6-phosphate dehydrogenase X-linked	(0.609)	7.12	6.89E-12
Tufm	233870	Tu translation elongation factor, mitochondrial	(0.609)	25.46	7.11E-25
Rin2	74030	Ras and Rab interactor 2	(0.610)	0.98	3.11E-04
Fam175a	70681	family with sequence similarity 175, member A	(0.610)	4.89	2.63E-09
Trappc12	217449	trafficking protein particle complex 12	(0.610)	2.59	6.20E-08
Mccc1	72039	methylcrotonoyl-Coenzyme A carboxylase 1 (alpha)	(0.610)	5.04	1.08E-09
Pebp1	23980	phosphatidylethanolamine binding protein 1	(0.610)	9.98	2.56E-08
Mapre3	100732	microtubule-associated protein, RP/EB family, member 3	(0.610)	3.26	1.46E-05
Vwa9	69882	von Willebrand factor A domain containing 9	(0.610)	5.57	1.21E-09
Nln	75805	neurolysin (metallopeptidase M3 family)	(0.611)	5.41	1.12E-14
Tspyl1	22110	testis-specific protein, Y-encoded-like 1	(0.611)	6.96	3.08E-11

Official Gene Symbol	Entrez Gene ID	Gene description	Normalized Fold Change LOG2(Cryo-2 / Fresh-1)	FPKM Mean of six samples	p-value of Cryo-2 vs Fresh-1
Inpp5d	16331	inositol polyphosphate-5-phosphatase D	(0.611)	7.80	4.02E-19
Hdx	245596	highly divergent homeobox	(0.611)	0.93	5.38E-03
Aen	68048	apoptosis enhancing nuclease	(0.611)	3.93	2.37E-07
Sympk	68188	symplekin	(0.612)	8.73	4.01E-26
Cpsf3l	71957	cleavage and polyadenylation specific factor 3-like	(0.612)	8.38	2.33E-12
Stat2	20847	signal transducer and activator of transcription 2	(0.613)	1.13	4.69E-04
Stard9	668880	-	(0.613)	0.73	2.02E-08
Atf7	223922	activating transcription factor 7	(0.613)	1.28	4.36E-03
Rpl35a-ps4	100043423	ribosomal protein 35A, pseudogene 4	(0.613)	4.37	4.64E-02
Spata13	219140	spermatogenesis associated 13	(0.614)	2.25	6.25E-12
Ctdnep1	67181	CTD nuclear envelope phosphatase 1	(0.614)	10.18	8.85E-10
Urod	22275	uroporphyrinogen decarboxylase	(0.615)	5.77	2.03E-05
Akap11	219181	A kinase (PRKA) anchor protein 11	(0.615)	0.51	4.90E-04
Rad17	19356	RAD17 homolog (S. pombe)	(0.616)	4.64	1.42E-09
Rnf25	57751	ring finger protein 25	(0.616)	3.69	2.71E-05
Esyt3	272636	extended synaptotagmin-like protein 3	(0.617)	1.41	1.71E-06
Creld2	76737	cysteine-rich with EGF-like domains 2	(0.617)	22.44	8.64E-17
Pdzk1	59020	PDZ domain containing 1	(0.617)	4.05	3.68E-09
Syvn1	74126	synovial apoptosis inhibitor 1, synoviolin	(0.618)	1.03	2.09E-04
Zfp583	213011	zinc finger protein 583	(0.618)	0.69	2.88E-03
Nadk2	68646	NAD kinase 2, mitochondrial	(0.619)	2.04	1.72E-05
S1pr2	14739	sphingosine-1-phosphate receptor 2	(0.620)	0.88	3.38E-03
Crkl	12929	v-crk sarcoma virus CT10 oncogene homolog (avian)-like	(0.620)	5.27	1.11E-14
Gsdma3	450219	gasdermin A3	(0.620)	1.12	3.37E-02
Bloc1s5	17828	biogenesis of lysosomal organelles complex-1, subunit 5, muted	(0.620)	3.54	4.08E-07
Hapln4	330790	hyaluronan and proteoglycan link protein 4	(0.621)	2.19	1.45E-05
Cpxm1	56264	carboxypeptidase X 1 (M14 family)	(0.621)	2.66	1.02E-04
Irak2	108960	interleukin-1 receptor-associated kinase 2	(0.621)	6.84	1.45E-15
Prkacb	18749	protein kinase, cAMP dependent, catalytic, beta	(0.621)	2.74	8.53E-09
Slc2a8	56017	solute carrier family 2, (facilitated glucose transporter), member 8	(0.622)	4.10	2.08E-06
Yaf2	67057	YY1 associated factor 2	(0.622)	2.54	1.85E-04
Ergic3	66366	ERGIC and golgi 3	(0.622)	9.67	5.11E-08
Gab2	14389	growth factor receptor bound protein 2-associated protein 2	(0.623)	1.40	8.84E-06
Sfrp1	20377	secreted frizzled-related protein 1	(0.623)	2.55	3.15E-07
Scp2	20280	sterol carrier protein 2, liver	(0.623)	16.97	1.20E-21
Synrg	217030	synergin, gamma	(0.623)	2.15	4.41E-11
Mrps22	64655	mitochondrial ribosomal protein S22	(0.623)	29.99	9.51E-19
Rxrb	20182	retinoid X receptor beta	(0.623)	2.68	1.45E-05
Fry	320365	furry homolog (Drosophila)	(0.624)	0.71	2.56E-06
Gapvd1	66691	GTPase activating protein and VPS9 domains 1	(0.624)	6.43	8.97E-23
Gas2	14453	growth arrest specific 2	(0.625)	0.55	1.35E-02
Asb6	72323	ankyrin repeat and SOCS box-containing 6	(0.625)	1.97	1.01E-03
Sfxn1	14057	sideroflexin 1	(0.625)	5.71	2.21E-10
Tmem11	216821	transmembrane protein 11	(0.625)	5.86	1.06E-05
Dusp6	67603	dual specificity phosphatase 6	(0.626)	3.44	5.18E-07
Spsb4	211949	splA/ryanodine receptor domain and SOCS box containing 4	(0.626)	4.03	8.53E-07
Pmpcb	73078	peptidase (mitochondrial processing) beta	(0.626)	23.23	7.01E-17

Official Gene Symbol	Entrez Gene ID	Gene description	Normalized Fold Change LOG2(Cryo-2 / Fresh-1)	FPKM Mean of six samples	p-value of Cryo-2 vs Fresh-1
Dffb	13368	DNA fragmentation factor, beta subunit	(0.626)	3.64	5.54E-07
Prkar1a	19084	protein kinase, cAMP dependent regulatory, type I, alpha	(0.626)	10.12	2.34E-24
Map3k3	26406	mitogen-activated protein kinase kinase kinase 3	(0.627)	5.72	6.38E-17
Adprh	11544	ADP-ribosylarginine hydrolase	(0.627)	10.41	2.77E-11
Pde7a	18583	phosphodiesterase 7A	(0.627)	2.15	1.07E-11
Cnnm2	94219	cyclin M2	(0.628)	1.49	2.30E-08
AU022252	230696	expressed sequence AU022252	(0.628)	1.02	1.27E-04
Bcl7b	12054	B cell CLL/lymphoma 7B	(0.629)	3.15	4.29E-04
Stambp	70527	STAM binding protein	(0.629)	1.82	1.63E-04
Ndnl2	66647	necdin-like 2	(0.630)	8.37	3.74E-08
Golga2	99412	golgi autoantigen, golgin subfamily a, 2	(0.630)	4.94	1.72E-14
Atad1	67979	ATPase family, AAA domain containing 1	(0.630)	10.63	7.50E-20
Ankrd49	56503	ankyrin repeat domain 49	(0.631)	6.03	7.40E-11
Tfrc	22042	transferrin receptor	(0.632)	7.29	2.46E-19
Lsg1	224092	large subunit GTPase 1 homolog (S. cerevisiae)	(0.633)	14.51	5.24E-20
Gga3	260302	golgi associated, gamma adaptin ear containing, ARF binding protein 3	(0.633)	3.17	3.44E-08
Dhcr24	74754	24-dehydrocholesterol reductase	(0.634)	4.21	6.18E-12
Commd6	66200	COMM domain containing 6	(0.634)	16.43	3.04E-10
Ilf2	67781	interleukin enhancer binding factor 2	(0.634)	12.84	5.68E-12
Crnkl1	66877	Crn, crooked neck-like 1 (Drosophila)	(0.634)	15.58	3.23E-22
Spc25	66442	SPC25, NDC80 kinetochore complex component, homolog (S. cerevisiae)	(0.635)	13.98	1.16E-15
Tmem29	382245	transmembrane protein 29	(0.635)	3.24	9.33E-08
Nktr	18087	natural killer tumor recognition sequence	(0.636)	5.77	2.17E-30
Rnaset2b	68195	ribonuclease T2B	(0.636)	20.01	6.24E-11
Gopc	94221	golgi associated PDZ and coiled-coil motif containing	(0.637)	1.71	7.13E-06
Zfp639	67778	zinc finger protein 639	(0.637)	2.41	2.39E-06
Tmem164	209497	transmembrane protein 164	(0.637)	0.81	6.58E-05
Slc23a2	54338	solute carrier family 23 (nucleobase transporters), member 2	(0.637)	2.26	1.92E-11
Lnp	69605	limb and neural patterns	(0.637)	2.45	5.30E-12
Kbtbd8	243574	kelch repeat and BTB (POZ) domain containing 8	(0.638)	2.54	1.89E-06
Git2	26431	G protein-coupled receptor kinase-interactor 2	(0.638)	4.35	1.56E-15
Tut1	70044	terminal uridylyl transferase 1, U6 snRNA-specific	(0.638)	2.27	4.05E-05
Cttn	13043	cortactin	(0.638)	34.25	1.13E-51
Tmed7	66676	transmembrane emp24 protein transport domain containing 7	(0.639)	11.80	5.78E-21
Spty2d1	101685	SPT2, Suppressor of Ty, domain containing 1 (S. cerevisiae)	(0.639)	5.97	1.52E-18
Man2b2	17160	mannosidase 2, alpha B2	(0.640)	1.71	4.81E-05
Sumf1	58911	sulfatase modifying factor 1	(0.640)	2.13	6.54E-06
Pramef12	77632	PRAME family member 12	(0.641)	6.05	1.62E-11
Primpol	408022	primase and polymerase (DNA-directed)	(0.641)	2.16	5.87E-07
C2	12263	complement component 2 (within H-2S)	(0.642)	1.82	3.77E-04
Fastkd2	75619	FAST kinase domains 2	(0.643)	6.74	4.66E-14
Gca	227960	grancalcin	(0.644)	8.03	1.37E-14
Cdkn2d	12581	cyclin-dependent kinase inhibitor 2D (p19, inhibits CDK4)	(0.644)	1.79	1.30E-02
Inadl	12695	InaD-like (Drosophila)	(0.644)	2.36	3.86E-16

Official Gene Symbol	Entrez Gene ID	Gene description	Normalized Fold Change LOG2(Cryo-2 / Fresh-1)	FPKM Mean of six samples	p-value of Cryo-2 vs Fresh-1
Grpel2	17714	GrpE-like 2, mitochondrial	(0.645)	3.21	7.47E-10
Dync2h1	110350	dynein cytoplasmic 2 heavy chain 1	(0.645)	1.00	1.28E-09
Nme4	56520	NME/NM23 nucleoside diphosphate kinase 4	(0.645)	8.37	2.66E-07
Cd81	12520	CD81 antigen	(0.646)	3.51	5.64E-04
Tex19.2	70956	testis expressed gene 19.2	(0.646)	3.77	2.79E-06
Uba5	66663	ubiquitin-like modifier activating enzyme 5	(0.646)	7.11	1.02E-02
Pbk	52033	PDZ binding kinase	(0.647)	13.97	5.36E-16
LOC102636110	102636110	-	(0.647)	1.47	1.10E-09
Myo6	17920	myosin VI	(0.647)	1.94	4.24E-11
Hs2st1	23908	heparan sulfate 2-O-sulfotransferase 1	(0.647)	3.41	6.23E-16
Tsr3	68327	TSR3 20S rRNA accumulation	(0.647)	8.10	1.04E-07
Kctd2	70382	potassium channel tetramerisation domain containing 2	(0.647)	1.79	8.96E-04
Eral1	57837	Era (G-protein)-like 1 (E. coli)	(0.648)	2.39	2.41E-04
Bora	77744	bora, aurora kinase A activator	(0.649)	2.76	3.09E-10
Phyh	16922	phytanoyl-CoA hydroxylase	(0.649)	20.83	2.80E-16
Spopl	76857	speckle-type POZ protein-like	(0.649)	1.62	2.79E-07
Neto2	74513	neuropilin (NRP) and tolloid (TLL)-like 2	(0.649)	1.44	4.83E-07
Eda2r	245527	ectodysplasin A2 receptor	(0.649)	1.84	1.18E-08
Cpsf4	54188	cleavage and polyadenylation specific factor 4	(0.650)	4.77	1.06E-06
Helb	117599	helicase (DNA) B	(0.651)	2.24	5.06E-08
Smoc1	64075	SPARC related modular calcium binding 1	(0.651)	2.67	7.89E-08
Ascc2	75452	activating signal cointegrator 1 complex subunit 2	(0.651)	6.80	7.44E-23
Vezt	215008	vezatin, adherens junctions transmembrane protein	(0.652)	1.08	6.72E-10
Ecsit	26940	ECSIT homolog (Drosophila)	(0.652)	2.87	4.78E-05
Rbm15b	109095	RNA binding motif protein 15B	(0.652)	1.61	3.60E-04
Cubn	65969	cubilin (intrinsic factor-cobalamin receptor)	(0.652)	5.99	1.50E-33
Clasrp	53609	CLK4-associating serine/arginine rich protein	(0.653)	4.22	2.59E-07
Ap4b1	67489	adaptor-related protein complex AP-4, beta 1	(0.653)	4.17	1.60E-09
C87977	97187	expressed sequence C87977	(0.653)	1.22	1.36E-02
Taok2	381921	TAO kinase 2	(0.653)	2.15	2.28E-11
Usp29	57775	ubiquitin specific peptidase 29	(0.654)	0.68	8.99E-05
Thap1	73754	THAP domain containing, apoptosis associated protein 1	(0.654)	2.03	9.22E-05
Lgals7	16858	lectin, galactose binding, soluble 7	(0.654)	5.00	7.43E-03
Elk1	13712	ELK1, member of ETS oncogene family	(0.655)	0.92	6.88E-03
Mgat5	107895	mannoside acetylglucosaminyltransferase 5	(0.655)	1.08	1.40E-07
Nsf	18195	N-ethylmaleimide sensitive fusion protein	(0.655)	9.74	6.06E-23
Ppil2	66053	peptidylprolyl isomerase (cyclophilin)-like 2	(0.656)	11.29	8.90E-15
Ggct	110175	gamma-glutamyl cyclotransferase	(0.656)	1.09	4.55E-02
Pigg	433931	phosphatidylinositol glycan anchor biosynthesis, class G	(0.656)	1.89	1.14E-05
Cdc42se1	57912	CDC42 small effector 1	(0.656)	3.28	3.22E-08
Anapc16	52717	anaphase promoting complex subunit 16	(0.656)	5.30	7.58E-08
Thoc5	107829	THO complex 5	(0.657)	11.56	7.74E-19
Hist1h2bk	319184	histone cluster 1, H2bk	(0.657)	84.55	3.67E-25
Nup85	445007	nucleoporin 85	(0.657)	15.49	4.15E-24
Cdc25b	12531	cell division cycle 25B	(0.657)	1.98	3.44E-05
Bop1	12181	block of proliferation 1	(0.657)	7.50	6.04E-14
Ap3d1	11776	adaptor-related protein complex 3, delta 1 subunit	(0.657)	14.03	5.57E-35
Sec22b	20333	SEC22 vesicle trafficking protein homolog B (S. cerevisiae)	(0.657)	9.31	8.81E-13

Official Gene Symbol	Entrez Gene ID	Gene description	Normalized Fold Change LOG2(Cryo-2 / Fresh-1)	FPKM Mean of six samples	p-value of Cryo-2 vs Fresh-1
Mid1ip1	68041	Mid1 interacting protein 1 (gastrulation specific G12-like (zebrafish))	(0.657)	2.48	4.22E-04
Tonsl	72749	tonsoku-like, DNA repair protein	(0.657)	2.14	2.49E-07
Mtg1	212508	mitochondrial GTPase 1 homolog (S. cerevisiae)	(0.658)	5.61	8.00E-08
Fubp3	320267	far upstream element (FUSE) binding protein 3	(0.658)	14.07	1.62E-31
Bysl	53414	bystin-like	(0.659)	5.51	7.28E-15
Gbf1	107338	golgi-specific brefeldin A-resistance factor 1	(0.659)	3.41	7.21E-16
Dpp9	224897	dipeptidylpeptidase 9	(0.659)	2.86	6.19E-09
Atg7	74244	autophagy related 7	(0.660)	4.44	2.24E-13
Dus4l	71916	dihydrouridine synthase 4-like (S. cerevisiae)	(0.660)	4.13	4.81E-06
Lrrc47	72946	leucine rich repeat containing 47	(0.660)	10.92	1.89E-25
Prkdc	19090	protein kinase, DNA activated, catalytic polypeptide	(0.660)	1.65	9.65E-15
Dync1li1	235661	dynein cytoplasmic 1 light intermediate chain 1	(0.660)	19.67	4.78E-25
Nudcd1	67429	NudC domain containing 1	(0.661)	7.96	1.70E-19
Sri	109552	sorcin	(0.661)	3.59	6.11E-09
Fbxo6	50762	F-box protein 6	(0.661)	8.53	2.95E-13
AW209491	105351	expressed sequence AW209491	(0.662)	3.05	4.49E-05
Rnf220	66743	ring finger protein 220	(0.662)	2.83	9.65E-11
Ndufa4	17992	NADH dehydrogenase (ubiquinone) 1 alpha subcomplex, 4	(0.662)	71.10	1.28E-19
Polr2c	20021	polymerase (RNA) II (DNA directed) polypeptide C	(0.663)	15.83	4.35E-13
Tle6	114606	transducin-like enhancer of split 6, homolog of Drosophila E(spl)	(0.663)	0.84	1.53E-02
Mpg	268395	N-methylpurine-DNA glycosylase	(0.663)	1.11	1.03E-02
Nudt9	74167	nudix (nucleoside diphosphate linked moiety X)-type motif 9	(0.663)	7.96	4.72E-10
Sh3tc1	231147	SH3 domain and tetratricopeptide repeats 1	(0.664)	2.58	5.55E-09
Hist1h2bm	319186	histone cluster 1, H2bm	(0.665)	78.60	3.01E-23
Ubn1	170644	ubinuclein 1	(0.665)	2.25	3.21E-11
Exd2	97827	exonuclease 3'-5' domain containing 2	(0.665)	2.78	8.61E-08
D2Wsu81e	227695	DNA segment, Chr 2, Wayne State University 81, expressed	(0.665)	8.56	6.45E-10
Mtg2	52856	mitochondrial ribosome associated GTPase 2	(0.666)	2.64	4.71E-06
Slc8b1	170756	solute carrier family 8 (sodium/lithium/calcium exchanger), member B1	(0.666)	4.64	4.46E-10
Mageb16	71967	melanoma antigen family B, 16	(0.666)	9.23	2.08E-12
Oat	18242	ornithine aminotransferase	(0.666)	10.08	5.90E-15
Slc25a14	20523	solute carrier family 25 (mitochondrial carrier, brain), member 14	(0.667)	1.54	3.88E-03
Dlst	78920	dihydrolipoamide S-succinyltransferase (E2 component of 2-oxo-glutarate complex)	(0.667)	14.10	1.98E-19
Plau	18792	plasminogen activator, urokinase	(0.668)	2.41	3.79E-05
Acaa1a	113868	acetyl-Coenzyme A acyltransferase 1A	(0.668)	3.16	9.19E-05
Polr3e	26939	polymerase (RNA) III (DNA directed) polypeptide E	(0.669)	3.38	2.79E-11
Nup107	103468	nucleoporin 107	(0.669)	10.88	6.26E-19
Zfp185	22673	zinc finger protein 185	(0.669)	1.62	6.53E-05
Fam134a	227298	family with sequence similarity 134, member A	(0.669)	3.22	6.11E-07
Acadvl	11370	acyl-Coenzyme A dehydrogenase, very long chain	(0.669)	7.38	1.48E-11
Dda1	66498	DET1 and DDB1 associated 1	(0.669)	13.29	4.57E-17
Plcg1	18803	phospholipase C, gamma 1	(0.670)	2.51	4.64E-09
Ifitm2	80876	interferon induced transmembrane protein 2	(0.670)	4.24	6.34E-03
Rps6ka6	67071	ribosomal protein S6 kinase polypeptide 6	(0.670)	0.65	1.06E-03

Official Gene Symbol	Entrez Gene ID	Gene description	Normalized Fold Change LOG2(Cryo-2 / Fresh-1)	FPKM Mean of six samples	p-value of Cryo-2 vs Fresh-1
Ccdc71	72454	coiled-coil domain containing 71	(0.671)	6.29	4.35E-15
Necab3	56846	N-terminal EF-hand calcium binding protein 3	(0.671)	1.74	8.26E-04
Ddrgk1	77006	DDRGK domain containing 1	(0.672)	4.65	2.80E-12
Lurap1l	52829	leucine rich adaptor protein 1-like	(0.672)	2.47	3.66E-04
Plgrkt	67759	plasminogen receptor, C-terminal lysine transmembrane protein	(0.673)	3.44	1.86E-03
Cdk5rap3	80280	CDK5 regulatory subunit associated protein 3	(0.674)	4.03	1.03E-07
Bhlhb9	70237	basic helix-loop-helix domain containing, class B9	(0.674)	0.78	9.56E-03
Optn	71648	optineurin	(0.674)	6.03	4.09E-12
Ddr2	18214	discoidin domain receptor family, member 2	(0.675)	1.64	2.85E-11
Rint1	72772	RAD50 interactor 1	(0.675)	3.32	6.46E-09
Vill	22351	villin-like	(0.675)	3.23	1.83E-07
Duxbl1	278672	double homeobox B-like 1	(0.675)	0.62	8.27E-03
Cdca5	67849	cell division cycle associated 5	(0.675)	9.89	2.49E-14
Zfp719	210105	zinc finger protein 719	(0.675)	2.23	1.28E-07
Apobr	171504	apolipoprotein B receptor	(0.675)	0.73	5.15E-03
Elof1	66126	elongation factor 1 homolog (ELF1, S. cerevisiae)	(0.675)	10.54	8.93E-07
Dcun1d2	102323	DCN1, defective in cullin neddylation 1, domain containing 2 (S. cerevisiae)	(0.676)	1.78	8.29E-05
Ydjc	69101	YdjC homolog (bacterial)	(0.677)	2.67	3.44E-03
Nelfcd	57314	negative elongation factor complex member C/D, Th1l	(0.677)	4.60	1.13E-08
Uba3	22200	ubiquitin-like modifier activating enzyme 3	(0.678)	11.55	6.03E-23
Zfp944	319615	zinc finger protein 944	(0.678)	1.59	1.45E-04
Rn7s1	103948	7S RNA 1	(0.678)	######	3.85E-53
Bad	12015	BCL2-associated agonist of cell death	(0.679)	2.11	4.34E-03
Ccs	12460	copper chaperone for superoxide dismutase	(0.679)	2.83	2.71E-03
Mrm1	217038	mitochondrial rRNA methyltransferase 1 homolog (S. cerevisiae)	(0.679)	1.42	1.60E-03
Fig4	103199	FIG4 homolog (S. cerevisiae)	(0.680)	7.52	3.72E-17
Nol4l	329540	nucleolar protein 4-like	(0.680)	2.80	5.52E-17
Cxxc1	74322	CXXC finger 1 (PHD domain)	(0.681)	5.75	3.87E-12
Rpp25l	69961	ribonuclease P/MRP 25 subunit-like	(0.681)	3.91	3.49E-04
Ddx50	94213	DEAD (Asp-Glu-Ala-Asp) box polypeptide 50	(0.682)	6.88	4.16E-16
Dhx35	71715	DEAH (Asp-Glu-Ala-His) box polypeptide 35	(0.682)	1.85	1.27E-08
Hoxb9	15417	homeobox B9	(0.683)	0.52	3.24E-03
Zfp507	668501	zinc finger protein 507	(0.683)	3.27	2.43E-15
Gulo	268756	gulonolactone (L-) oxidase	(0.684)	27.27	5.89E-39
Gatad1	67210	GATA zinc finger domain containing 1	(0.684)	13.03	8.68E-22
Ifnar1	15975	interferon (alpha and beta) receptor 1	(0.684)	1.94	5.16E-11
Sugp1	70616	SURP and G patch domain containing 1	(0.685)	6.51	1.92E-12
Rad52	19365	RAD52 homolog (S. cerevisiae)	(0.686)	1.43	2.07E-06
Wdr83	67836	WD repeat domain containing 83	(0.687)	3.36	5.21E-04
Ivd	56357	isovaleryl coenzyme A dehydrogenase	(0.687)	1.19	3.08E-04
Wdr44	72404	WD repeat domain 44	(0.688)	2.90	2.21E-02
Fam92a	68099	family with sequence similarity 92, member A	(0.688)	0.90	1.95E-02
Vps51	68505	vacuolar protein sorting 51 homolog (S. cerevisiae)	(0.688)	3.13	2.17E-07
Serinc2	230779	serine incorporator 2	(0.689)	2.48	2.52E-05
Afmid	71562	arylformamidase	(0.690)	2.63	1.09E-07
Tor4a	227612	torsin family 4, member A	(0.690)	0.67	1.56E-02
Mrpl49	18120	mitochondrial ribosomal protein L49	(0.691)	12.29	5.15E-16

Official Gene Symbol	Entrez Gene ID	Gene description	Normalized Fold Change LOG2(Cryo-2 / Fresh-1)	FPKM Mean of six samples	p-value of Cryo-2 vs Fresh-1
Mafk	17135	v-maf musculoaponeurotic fibrosarcoma oncogene family, protein K (avian)	(0.691)	0.56	8.63E-03
Vps53	68299	vacuolar protein sorting 53 (yeast)	(0.692)	6.24	2.42E-12
DXBay18	574405	DNA segment, Chr X, Baylor 18	(0.694)	0.63	8.31E-03
Etfdh	66841	electron transferring flavoprotein, dehydrogenase	(0.694)	7.00	5.01E-13
Anks1	224650	ankyrin repeat and SAM domain containing 1	(0.694)	1.33	2.41E-08
Cep95	320162	centrosomal protein 95	(0.695)	1.44	1.28E-03
Smug1	71726	single-strand selective monofunctional uracil DNA glycosylase	(0.696)	0.68	1.78E-04
Vps11	71732	vacuolar protein sorting 11 (yeast)	(0.697)	8.31	4.94E-24
Rhbdd2	215160	rhomboid domain containing 2	(0.697)	1.17	9.04E-05
Mmgt2	216829	membrane magnesium transporter 2	(0.698)	2.39	3.51E-05
Cyb5r4	266690	cytochrome b5 reductase 4	(0.698)	9.09	1.64E-17
Elp2	58523	elongator acetyltransferase complex subunit 2	(0.699)	8.00	3.26E-20
D6Wsu163e	28040	DNA segment, Chr 6, Wayne State University 163, expressed	(0.699)	5.68	6.23E-12
Taf2	319944	TAF2 RNA polymerase II, TATA box binding protein (TBP)-associated factor	(0.700)	8.44	8.51E-31
Med17	234959	mediator complex subunit 17	(0.700)	7.35	4.95E-23
Ebna1bp2	69072	EBNA1 binding protein 2	(0.701)	23.69	1.07E-43
Uchl1	22223	ubiquitin carboxy-terminal hydrolase L1	(0.701)	5.36	7.00E-06
Atp6v1f	66144	ATPase, H+ transporting, lysosomal V1 subunit F	(0.701)	22.47	6.10E-12
Phldb2	208177	pleckstrin homology-like domain, family B, member 2	(0.702)	0.64	5.33E-04
Endod1	71946	endonuclease domain containing 1	(0.702)	2.19	2.22E-08
Ppan	235036	peter pan homolog (Drosophila)	(0.702)	10.68	7.93E-14
Cript	56724	cysteine-rich PDZ-binding protein	(0.702)	24.58	1.83E-18
Lphn1	330814	latrophilin 1	(0.704)	1.09	9.18E-08
Ppm1f	68606	protein phosphatase 1F (PP2C domain containing)	(0.704)	2.28	4.50E-09
Stra13	20892	stimulated by retinoic acid 13	(0.704)	3.81	2.99E-07
Myo5a	17918	myosin VA	(0.704)	1.51	1.13E-14
H2-K1	14972	histocompatibility 2, K1, K region	(0.705)	21.87	3.82E-33
Ankrd50	99696	ankyrin repeat domain 50	(0.706)	1.28	3.13E-09
Sp110	109032	Sp110 nuclear body protein	(0.706)	3.71	2.90E-10
Elovl1	54325	elongation of very long chain fatty acids (FEN1/Elo2, SUR4/Elo3, yeast)-like 1	(0.707)	2.71	1.36E-07
Stk38l	232533	serine/threonine kinase 38 like	(0.708)	3.31	5.62E-17
Rtkn	20166	rhotekin	(0.708)	0.59	1.73E-04
Dynlt1a	100310872	dynein light chain Tctex-type 1A	(0.708)	1.68	1.85E-07
Mkln1	27418	muskelin 1, intracellular mediator containing kelch motifs	(0.709)	4.90	2.26E-24
Tgfbrap1	73122	transforming growth factor, beta receptor associated protein 1	(0.709)	1.53	8.04E-07
Rnf128	66889	ring finger protein 128	(0.710)	4.94	2.43E-13
Rel	19696	reticuloendotheliosis oncogene	(0.710)	1.96	3.58E-05
Cgrrf1	68755	cell growth regulator with ring finger domain 1	(0.710)	2.24	2.15E-03
Plagl1	22634	pleiomorphic adenoma gene-like 1	(0.711)	0.52	2.88E-03
Hsd17b10	15108	hydroxysteroid (17-beta) dehydrogenase 10	(0.711)	9.26	1.01E-07
Herpud2	80517	HERPUD family member 2	(0.711)	4.75	5.46E-09
Hist1h2bc	68024	histone cluster 1, H2bc	(0.712)	68.81	2.13E-26
Dgat2	67800	diacylglycerol O-acyltransferase 2	(0.712)	1.34	1.67E-03
Nop2	110109	NOP2 nucleolar protein	(0.712)	10.86	1.65E-20

Official Gene Symbol	Entrez Gene ID	Gene description	Normalized Fold Change LOG2(Cryo-2 / Fresh-1)	FPKM Mean of six samples	p-value of Cryo-2 vs Fresh-1
Cwc22	80744	CWC22 spliceosome-associated protein homolog (S. cerevisiae)	(0.713)	9.93	2.34E-23
Crocc	230872	ciliary rootlet coiled-coil, rootletin	(0.713)	1.29	9.48E-08
Vimp	109815	VCP-interacting membrane protein	(0.713)	14.20	3.92E-14
Umad1	100036521	UMAP1-MVP12 associated (UMA) domain containing 1	(0.713)	0.86	2.14E-02
Tmeff1	230157	transmembrane protein with EGF-like and two follistatin-like domains 1	(0.713)	11.57	2.72E-23
Impa2	114663	inositol (myo)-1(or 4)-monophosphatase 2	(0.714)	7.36	1.77E-08
Suox	211389	sulfite oxidase	(0.714)	3.46	1.95E-07
Eif2ak1	15467	eukaryotic translation initiation factor 2 alpha kinase 1	(0.715)	9.06	8.93E-18
Zwilch	68014	zwilch kinetochore protein	(0.716)	15.17	3.11E-27
Zfp955b	100043468	zinc finger protein 955B	(0.716)	3.34	1.11E-09
Vapb	56491	vesicle-associated membrane protein, associated protein B and C	(0.717)	2.41	3.19E-14
Bnip1	224630	BCL2/adenovirus E1B interacting protein 1	(0.717)	3.84	1.28E-06
A4galt	239559	alpha 1,4-galactosyltransferase	(0.719)	8.69	2.22E-15
Ndufa9	66108	NADH dehydrogenase (ubiquinone) 1 alpha subcomplex, 9	(0.719)	18.48	5.77E-19
Tango6	272538	transport and golgi organization 6	(0.719)	0.92	2.16E-04
Aktip	14339	thymoma viral proto-oncogene 1 interacting protein	(0.720)	3.68	9.43E-17
Hibch	227095	3-hydroxyisobutyryl-Coenzyme A hydrolase	(0.721)	4.82	1.23E-07
Cidea	12683	cell death-inducing DNA fragmentation factor, alpha subunit-like effector A	(0.721)	2.47	3.07E-03
Chtf18	214901	CTF18, chromosome transmission fidelity factor 18	(0.721)	3.24	1.54E-09
LOC102639713	102639713	-	(0.722)	1.51	1.13E-05
Eepd1	67484	endonuclease/exonuclease/phosphatase family domain containing 1	(0.722)	0.59	2.10E-02
Atp11a	50770	ATPase, class VI, type 11A	(0.722)	1.11	6.30E-10
Kifc3	16582	kinesin family member C3	(0.723)	2.98	1.41E-10
Ammecr1	56068	Alport syndrome, mental retardation, midface hypoplasia and elliptocytosis chromosomal region gene 1	(0.723)	2.23	8.59E-06
Slc25a38	208638	solute carrier family 25, member 38	(0.723)	1.32	9.25E-05
Pfdn6	14976	prefoldin subunit 6	(0.724)	43.05	5.67E-23
Cuedc2	67116	CUE domain containing 2	(0.724)	2.99	8.65E-06
Bloc1s2	73689	biogenesis of lysosomal organelles complex-1, subunit 2	(0.725)	17.24	4.62E-14
Phtf1	18685	putative homeodomain transcription factor 1	(0.725)	1.43	3.29E-05
Trak2	70827	trafficking protein, kinesin binding 2	(0.726)	5.31	5.53E-18
Zfp759	268670	zinc finger protein 759	(0.726)	2.16	1.74E-08
Eaf1	74427	ELL associated factor 1	(0.727)	3.03	1.68E-09
Ufl1	67490	UFM1 specific ligase 1	(0.727)	5.77	8.89E-25
Gpr4	319197	G protein-coupled receptor 4	(0.728)	2.69	3.77E-07
Pkig	18769	protein kinase inhibitor, gamma	(0.728)	9.07	3.64E-11
Zbtb22	81630	zinc finger and BTB domain containing 22	(0.728)	0.94	7.16E-03
Arl6ip6	65103	ADP-ribosylation factor-like 6 interacting protein 6	(0.729)	5.73	1.12E-10
Rab24	19336	RAB24, member RAS oncogene family	(0.729)	3.90	2.05E-06
Uvrag	78610	UV radiation resistance associated gene	(0.729)	2.29	1.54E-07
Grb7	14786	growth factor receptor bound protein 7	(0.729)	2.97	5.02E-07
Glrx2	69367	glutaredoxin 2 (thioltransferase)	(0.730)	1.03	2.54E-05

Official Gene Symbol	Entrez Gene ID	Gene description	Normalized Fold Change LOG2(Cryo-2 / Fresh-1)	FPKM Mean of six samples	p-value of Cryo-2 vs Fresh-1
Klc1	16593	kinesin light chain 1	(0.730)	2.04	2.19E-09
Bhmt-ps1	57421	betaine-homocysteine methyltransferase, pseudogene 1	(0.730)	1.62	1.57E-03
Zfp800	627049	zinc finger protein 800	(0.731)	2.41	3.53E-19
Rab28	100972	RAB28, member RAS oncogene family	(0.732)	1.35	4.25E-03
Shcbp1	20419	Shc SH2-domain binding protein 1	(0.732)	11.44	2.74E-19
Hmbs	15288	hydroxymethylbilane synthase	(0.733)	5.25	2.52E-08
Erlin2	244373	ER lipid raft associated 2	(0.734)	6.04	2.00E-23
Spire1	68166	spire homolog 1 (Drosophila)	(0.734)	0.54	1.85E-03
Zfp54	22712	zinc finger protein 54	(0.734)	1.19	9.86E-05
Polg	18975	polymerase (DNA directed), gamma	(0.734)	3.50	3.06E-13
Dedd	21945	death effector domain-containing	(0.735)	5.99	4.49E-20
Dusp12	80915	dual specificity phosphatase 12	(0.735)	4.55	1.79E-06
Aifm1	26926	apoptosis-inducing factor, mitochondrion-associated 1	(0.736)	9.74	5.03E-19
Phf21b	271305	PHD finger protein 21B	(0.737)	0.52	1.03E-02
Stx18	71116	syntaxin 18	(0.737)	3.06	4.01E-09
Enthd2	78777	ENTH domain containing 2	(0.738)	1.40	2.31E-04
Clmp	71566	CXADR-like membrane protein	(0.739)	1.24	4.70E-06
Riok1	71340	RIO kinase 1 (yeast)	(0.739)	9.30	4.59E-18
Tab1	66513	TGF-beta activated kinase 1/MAP3K7 binding protein 1	(0.739)	0.51	2.71E-02
Prr7	432763	proline rich 7 (synaptic)	(0.740)	1.24	2.26E-02
Gimap9	317758	GTPase, IMAP family member 9	(0.740)	1.77	1.01E-02
Tbc1d10a	103724	TBC1 domain family, member 10a	(0.740)	2.07	9.09E-05
Qrsl1	76563	glutaminyl-tRNA synthase (glutamine-hydrolyzing)-like 1	(0.741)	6.11	4.34E-11
Kif18a	228421	kinesin family member 18A	(0.741)	6.01	5.54E-05
P4ha1	18451	procollagen-proline, 2-oxoglutarate 4-dioxygenase (proline 4-hydroxylase), alpha 1 polypeptide	(0.742)	5.99	9.65E-19
Fbxl6	30840	F-box and leucine-rich repeat protein 6	(0.742)	3.80	1.14E-06
Tmx4	52837	thioredoxin-related transmembrane protein 4	(0.742)	4.52	2.31E-19
Pfkfb1	18639	6-phosphofructo-2-kinase/fructose-2,6-biphosphatase 1	(0.743)	1.11	7.36E-04
Rab4a	19341	RAB4A, member RAS oncogene family	(0.743)	6.28	3.48E-09
E2f4	104394	E2F transcription factor 4	(0.743)	12.43	4.64E-19
Rae1	66679	RAE1 RNA export 1 homolog (S. pombe)	(0.743)	18.18	8.11E-26
Fmnl3	22379	formin-like 3	(0.743)	3.13	5.99E-13
Pmm2	54128	phosphomannomutase 2	(0.744)	9.01	3.22E-14
Cep70	68121	centrosomal protein 70	(0.744)	2.18	8.48E-11
Rwdd4a	192174	RWD domain containing 4A	(0.744)	5.94	1.01E-13
Dpp7	83768	dipeptidylpeptidase 7	(0.745)	4.29	2.84E-06
Gtf2e2	68153	general transcription factor II E, polypeptide 2 (beta subunit)	(0.746)	4.43	1.50E-17
Als2	74018	amyotrophic lateral sclerosis 2 (juvenile)	(0.747)	3.44	4.39E-21
Bcl9l	80288	B cell CLL/lymphoma 9-like	(0.748)	1.35	6.92E-10
Gmfg	63986	glia maturation factor, gamma	(0.749)	1.26	1.69E-03
Amdhd2	245847	amidohydrolase domain containing 2	(0.749)	6.58	2.40E-09
Zfp790	233056	zinc finger protein 790	(0.750)	1.29	4.87E-09
Prodh	19125	proline dehydrogenase	(0.750)	0.68	1.89E-02
Klhl18	270201	kelch-like 18	(0.750)	0.82	3.89E-04
Scamp1	107767	secretory carrier membrane protein 1	(0.752)	4.66	1.55E-14

Official Gene Symbol	Entrez Gene ID	Gene description	Normalized Fold Change LOG2(Cryo-2 / Fresh-1)	FPKM Mean of six samples	p-value of Cryo-2 vs Fresh-1
Prkaa2	108079	protein kinase, AMP-activated, alpha 2 catalytic subunit	(0.753)	0.91	2.64E-06
U2af1l4	233073	U2 small nuclear RNA auxiliary factor 1-like 4	(0.753)	5.54	1.92E-04
Guca1a	14913	guanylate cyclase activator 1a (retina)	(0.753)	14.36	1.25E-12
R3hcc1l	52013	R3H domain and coiled-coil containing 1 like	(0.753)	1.71	1.41E-07
Yars2	70120	tyrosyl-tRNA synthetase 2 (mitochondrial)	(0.754)	6.74	5.28E-10
Numbl	18223	numb-like	(0.755)	0.64	1.06E-03
Tusc2	80385	tumor suppressor candidate 2	(0.755)	4.31	7.44E-07
Taf1c	21341	TATA box binding protein (Tbp)-associated factor, RNA polymerase I, C	(0.755)	1.72	1.88E-06
Tmem194	210035	transmembrane protein 194	(0.756)	2.16	8.82E-12
Aar2	68295	AAR2 splicing factor homolog (S. cerevisiae)	(0.756)	2.08	2.33E-07
Lmf1	76483	lipase maturation factor 1	(0.756)	0.74	4.42E-02
Dsc2	13506	desmocollin 2	(0.756)	3.39	7.42E-14
Tspyl2	52808	TSPY-like 2	(0.757)	1.16	7.66E-04
Mvd	192156	mevalonate (diphospho) decarboxylase	(0.757)	1.86	6.24E-04
Dapk3	13144	death-associated protein kinase 3	(0.758)	6.04	2.55E-11
Zfp709	236193	zinc finger protein 709	(0.758)	0.86	7.94E-04
Ece2	107522	endothelin converting enzyme 2	(0.759)	0.98	3.12E-07
Nudt12	67993	nudix (nucleoside diphosphate linked moiety X)-type motif 12	(0.759)	4.58	8.50E-15
Lman2l	214895	lectin, mannose-binding 2-like	(0.761)	0.73	1.74E-02
Eda	13607	ectodysplasin-A	(0.761)	0.85	5.14E-05
Nrde2	217827	nrde-2 necessary for RNA interference, domain containing	(0.761)	3.94	1.74E-15
Ddx23	74351	DEAD (Asp-Glu-Ala-Asp) box polypeptide 23	(0.761)	17.48	4.77E-35
Lhfpl2	218454	lipoma HMGIC fusion partner-like 2	(0.761)	0.76	2.96E-06
Exo5	73172	exonuclease 5	(0.762)	1.74	5.37E-04
Zfp599	235048	zinc finger protein 599	(0.762)	7.08	8.68E-22
Tmem216	68642	transmembrane protein 216	(0.763)	2.01	4.78E-03
Zfp341	228807	zinc finger protein 341	(0.764)	0.92	1.13E-03
Nop9	67842	NOP9 nucleolar protein	(0.764)	3.86	1.05E-13
Wrap73	59002	WD repeat domain 8	(0.765)	1.48	1.90E-04
Zbtb40	230848	zinc finger and BTB domain containing 40	(0.766)	0.90	2.04E-07
Tpgs1	110012	tubulin polyglutamylase complex subunit 1	(0.767)	2.88	1.02E-03
Kcnu1	16532	potassium channel, subfamily U, member 1	(0.767)	1.07	8.22E-06
Ptgis	19223	prostaglandin I2 (prostacyclin) synthase	(0.768)	3.37	2.77E-06
Prmt3	71974	protein arginine N-methyltransferase 3	(0.770)	5.51	1.35E-12
Chmp6	208092	charged multivesicular body protein 6	(0.770)	3.11	1.34E-05
Ip6k2	76500	inositol hexaphosphate kinase 2	(0.770)	1.96	1.36E-06
Guf1	231279	GUF1 GTPase homolog (S. cerevisiae)	(0.771)	1.47	8.08E-06
Gpr108	78308	G protein-coupled receptor 108	(0.771)	3.09	8.25E-09
Nacc1	66830	nucleus accumbens associated 1, BEN and BTB (POZ) domain containing	(0.772)	7.82	7.49E-27
Zfyve1	217695	zinc finger, FYVE domain containing 1	(0.772)	3.68	9.31E-17
Cactin	70312	cactin, spliceosome C complex subunit	(0.773)	2.70	1.55E-07
Rn7s2	103949	7S RNA 2	(0.773)	384.21	5.50E-52
Scyl1	78891	SCY1-like 1 (S. cerevisiae)	(0.774)	4.95	3.58E-13
Ly6f	17071	lymphocyte antigen 6 complex, locus F	(0.774)	3.36	1.42E-03
Ptplad1	57874	protein tyrosine phosphatase-like A domain containing 1	(0.774)	3.82	1.73E-12
Zdhhc18	503610	zinc finger, DHHC domain containing 18	(0.774)	1.00	3.76E-05

Official Gene Symbol	Entrez Gene ID	Gene description	Normalized Fold Change LOG2(Cryo-2 / Fresh-1)	FPKM Mean of six samples	p-value of Cryo-2 vs Fresh-1
Zfp33b	69623	-	(0.774)	6.38	1.66E-21
Zdhhc16	74168	zinc finger, DHHC domain containing 16	(0.775)	4.93	4.36E-09
E2f8	108961	E2F transcription factor 8	(0.776)	2.16	1.49E-08
Ubash3b	72828	ubiquitin associated and SH3 domain containing, B	(0.776)	0.65	1.55E-05
Sohlh2	74434	spermatogenesis and oogenesis specific basic helix-loop-helix 2	(0.776)	1.61	1.58E-04
Sapcd2	72080	suppressor APC domain containing 2	(0.777)	2.16	1.30E-07
Tmem234	76799	transmembrane protein 234	(0.777)	12.63	1.85E-17
Pla2g6	53357	phospholipase A2, group VI	(0.777)	1.71	2.67E-07
Psmd9	67151	proteasome (prosome, macropain) 26S subunit, non-ATPase, 9	(0.778)	5.22	2.88E-13
Efr3a	76740	EFR3 homolog A (S. cerevisiae)	(0.778)	8.93	3.39E-37
Ddx52	78394	DEAD (Asp-Glu-Ala-Asp) box polypeptide 52	(0.779)	14.08	9.74E-37
Rgl2	19732	ral guanine nucleotide dissociation stimulator-like 2	(0.779)	1.04	2.31E-05
Kdm5d	20592	lysine (K)-specific demethylase 5D	(0.781)	0.90	1.05E-05
Cwc25	67480	CWC25 spliceosome-associated protein homolog (S. cerevisiae)	(0.781)	3.86	2.83E-12
Pacsin3	80708	protein kinase C and casein kinase substrate in neurons 3	(0.781)	1.94	1.21E-06
Timeless	21853	timeless circadian clock 1	(0.782)	3.03	4.16E-19
Nanp	67311	N-acetylneuraminic acid phosphatase	(0.782)	2.98	2.72E-05
Rbck1	24105	RanBP-type and C3HC4-type zinc finger containing 1	(0.782)	3.58	1.23E-09
Ska3	219114	spindle and kinetochore associated complex subunit 3	(0.782)	7.66	6.71E-17
Nagk	56174	N-acetylglucosamine kinase	(0.783)	6.61	1.62E-10
Hmox1	15368	heme oxygenase (decycling) 1	(0.784)	5.27	6.81E-07
Slc12a6	107723	solute carrier family 12, member 6	(0.786)	1.62	3.65E-12
Ttc39c	72747	tetratricopeptide repeat domain 39C	(0.787)	1.17	1.38E-03
Stat5a	20850	signal transducer and activator of transcription 5A	(0.788)	1.28	8.04E-06
Diap2	54004	diaphanous homolog 2 (Drosophila)	(0.790)	3.63	3.49E-28
Prmt5	27374	protein arginine N-methyltransferase 5	(0.790)	29.84	5.09E-52
Ddx55	67848	DEAD (Asp-Glu-Ala-Asp) box polypeptide 55	(0.790)	4.07	1.20E-10
Paqr4	76498	progestin and adipoQ receptor family member IV	(0.791)	1.59	7.87E-04
Eif5a2	208691	eukaryotic translation initiation factor 5A2	(0.791)	0.51	2.15E-03
Cpeb3	208922	cytoplasmic polyadenylation element binding protein 3	(0.792)	1.51	8.39E-12
Asrgl1	66514	asparaginase like 1	(0.792)	1.15	1.48E-03
Map3k9	338372	mitogen-activated protein kinase kinase kinase 9	(0.793)	0.97	1.49E-11
Gps2	56310	G protein pathway suppressor 2	(0.795)	3.94	3.58E-08
As3mt	57344	arsenic (+3 oxidation state) methyltransferase	(0.796)	3.98	1.79E-06
Abhd5	67469	abhydrolase domain containing 5	(0.798)	3.16	1.61E-10
Zfp414	328801	zinc finger protein 414	(0.798)	5.31	1.73E-06
Map3k4	26407	mitogen-activated protein kinase kinase kinase 4	(0.798)	4.56	6.00E-24
Tmem101	76547	transmembrane protein 101	(0.798)	1.57	3.55E-03
Disp1	68897	dispatched homolog 1 (Drosophila)	(0.798)	0.52	9.51E-03
Nfyb	18045	nuclear transcription factor-Y beta	(0.799)	11.46	8.59E-30
Phgdh	236539	3-phosphoglycerate dehydrogenase	(0.799)	49.96	1.21E-68
Fam118a	73225	family with sequence similarity 118, member A	(0.801)	4.64	1.54E-17
Cul4a	99375	cullin 4A	(0.801)	3.94	2.72E-15
Arhgap19	71085	Rho GTPase activating protein 19	(0.801)	3.94	4.02E-18
Ppp1r21	73825	protein phosphatase 1, regulatory subunit 21	(0.802)	3.96	3.67E-10

Official Gene Symbol	Entrez Gene ID	Gene description	Normalized Fold Change LOG2(Cryo-2 / Fresh-1)	FPKM Mean of six samples	p-value of Cryo-2 vs Fresh-1
Ano9	71345	anoctamin 9	(0.802)	0.96	4.28E-04
Prpf4	70052	PRP4 pre-mRNA processing factor 4 homolog (yeast)	(0.803)	4.53	4.10E-22
Polr1a	20019	polymerase (RNA) I polypeptide A	(0.803)	8.35	3.81E-42
Gtpbp2	56055	GTP binding protein 2	(0.804)	1.14	2.44E-04
Sh3yl1	24057	Sh3 domain YSC-like 1	(0.804)	3.14	2.10E-06
Tnfrsf22	79202	tumor necrosis factor receptor superfamily, member 22	(0.804)	0.85	2.07E-04
Nars2	244141	asparaginyl-tRNA synthetase 2 (mitochondrial)(putative)	(0.804)	1.25	2.02E-05
Fam3a	66294	family with sequence similarity 3, member A	(0.805)	1.85	1.11E-04
Aph1a	226548	anterior pharynx defective 1a homolog (C. elegans)	(0.806)	4.82	6.82E-16
Stxbp6	217517	syntaxin binding protein 6 (amisyn)	(0.807)	0.53	1.86E-03
Rad9a	19367	RAD9 homolog A	(0.807)	4.52	4.60E-10
Rnf215	71673	ring finger protein 215	(0.808)	0.66	3.96E-02
Clk3	102414	CDC-like kinase 3	(0.810)	3.79	2.82E-09
Svil	225115	supervillin	(0.810)	1.15	3.14E-12
Fgd4	224014	FYVE, RhoGEF and PH domain containing 4	(0.811)	1.75	3.09E-17
Cnnm3	94218	cyclin M3	(0.813)	1.35	2.24E-08
Rtn4r	65079	reticulon 4 receptor	(0.813)	1.09	8.37E-03
Tk2	57813	thymidine kinase 2, mitochondrial	(0.813)	0.78	3.59E-03
Crebzf	233490	CREB/ATF bZIP transcription factor	(0.814)	3.57	5.50E-19
Zfp383	73729	zinc finger protein 383	(0.815)	0.75	6.52E-03
Ddx19a	13680	DEAD (Asp-Glu-Ala-Asp) box polypeptide 19a	(0.815)	6.89	2.33E-19
LOC105244505	105244505	-	(0.815)	0.61	2.51E-02
Rpl39-ps	100503815	ribosomal protein L39, pseudogene	(0.816)	13.44	2.22E-05
Pcnxl3	104401	pecanex-like 3 (Drosophila)	(0.816)	1.79	1.22E-13
Btbd10	68815	BTB (POZ) domain containing 10	(0.817)	3.56	3.13E-12
Arhgef18	102098	rho/rac guanine nucleotide exchange factor (GEF) 18	(0.817)	2.48	9.67E-14
Atp8b1	54670	ATPase, class I, type 8B, member 1	(0.818)	1.17	2.84E-09
Pisd-ps2	328734	phosphatidylserine decarboxylase, pseudogene 2	(0.818)	0.57	8.31E-12
Snai3	30927	snail family zinc finger 3	(0.818)	0.71	3.15E-02
Scamp4	56214	secretory carrier membrane protein 4	(0.819)	10.17	1.01E-17
Deb1	26901	differentially expressed in B16F10 1	(0.821)	13.02	3.92E-07
Cep135	381644	centrosomal protein 135	(0.822)	1.52	2.12E-13
Ldoc1	434784	leucine zipper, down-regulated in cancer 1	(0.824)	0.94	3.00E-02
Kif22	110033	kinesin family member 22	(0.826)	19.33	1.41E-33
Pold1	18971	polymerase (DNA directed), delta 1, catalytic subunit	(0.827)	4.36	9.86E-14
S100a14	66166	S100 calcium binding protein A14	(0.827)	3.61	9.66E-05
Kap	16483	kidney androgen regulated protein	(0.827)	3.16	5.87E-03
Psmb10	19171	proteasome (prosome, macropain) subunit, beta type 10	(0.829)	1.28	7.38E-03
Fbxl18	231863	F-box and leucine-rich repeat protein 18	(0.830)	1.87	4.03E-07
Mmgt1	236792	membrane magnesium transporter 1	(0.830)	6.33	1.47E-25
Hectd3	76608	HECT domain containing 3	(0.831)	1.42	2.03E-07
Phykpl	72947	5-phosphohydroxy-L-lysine phospholyase	(0.831)	0.99	4.51E-04
Gabrd	14403	gamma-aminobutyric acid (GABA) A receptor, subunit delta	(0.833)	0.80	1.60E-02
AI464131	329828	expressed sequence AI464131	(0.833)	5.26	4.14E-26

Official Gene Symbol	Entrez Gene ID	Gene description	Normalized Fold Change LOG2(Cryo-2 / Fresh-1)	FPKM Mean of six samples	p-value of Cryo-2 vs Fresh-1
Pip4k2a	18718	phosphatidylinositol-5-phosphate 4-kinase, type II, alpha	(0.834)	0.89	2.41E-04
Pick1	18693	protein interacting with C kinase 1	(0.834)	3.73	9.98E-10
Btbd6	399566	BTB (POZ) domain containing 6	(0.834)	0.90	9.58E-05
Cenpk	60411	centromere protein K	(0.836)	3.22	6.93E-08
Trmu	72026	tRNA 5-methylaminomethyl-2-thiouridylate methyltransferase	(0.836)	0.72	1.47E-02
Naa60	74763	N(alpha)-acetyltransferase 60, NatF catalytic subunit	(0.837)	3.16	1.95E-10
Zbtb7a	16969	zinc finger and BTB domain containing 7a	(0.838)	0.86	1.18E-07
Tbc1d10b	68449	TBC1 domain family, member 10b	(0.838)	7.21	5.16E-26
Champ1	101994	chromosome alignment maintaining phosphoprotein 1	(0.838)	4.75	7.35E-21
C78339	97863	expressed sequence C78339	(0.840)	6.30	5.23E-23
Enpp3	209558	ectonucleotide pyrophosphatase/phosphodiesterase 3	(0.841)	1.17	1.62E-04
Guk1	14923	guanylate kinase 1	(0.843)	8.32	4.84E-10
Limd1	29806	LIM domains containing 1	(0.844)	2.46	2.62E-14
Piwil2	57746	piwi-like RNA-mediated gene silencing 2	(0.844)	2.85	8.82E-20
Trmt1	212528	tRNA methyltransferase 1	(0.847)	4.93	1.99E-18
Cep72	74470	centrosomal protein 72	(0.849)	0.83	3.10E-05
Lin52	217708	lin-52 homolog (C. elegans)	(0.850)	1.21	4.01E-05
Gtpbp6	107999	GTP binding protein 6 (putative)	(0.851)	2.01	1.02E-04
Lrrc41	230654	leucine rich repeat containing 41	(0.852)	4.02	1.69E-14
Ube2f	67921	ubiquitin-conjugating enzyme E2F (putative)	(0.852)	5.01	6.45E-13
Rnf31	268749	ring finger protein 31	(0.852)	1.56	1.55E-06
Hist1h2bj	319183	histone cluster 1, H2bj	(0.852)	55.62	1.19E-06
Klhl24	75785	kelch-like 24	(0.852)	1.65	1.78E-15
Pigo	56703	phosphatidylinositol glycan anchor biosynthesis, class O	(0.853)	0.84	7.19E-06
Kdm5c	20591	lysine (K)-specific demethylase 5C	(0.854)	3.31	1.08E-36
Man1b1	227619	mannosidase, alpha, class 1B, member 1	(0.855)	3.55	1.35E-15
Cpt2	12896	carnitine palmitoyltransferase 2	(0.856)	4.07	2.39E-11
Rbm10	236732	RNA binding motif protein 10	(0.856)	7.61	3.42E-28
Taco1	70207	translational activator of mitochondrially encoded cytochrome c oxidase I	(0.856)	3.07	1.33E-06
Pcmt1	18537	protein-L-isoaspartate (D-aspartate) O-methyltransferase 1	(0.859)	3.36	2.18E-18
Serac1	321007	serine active site containing 1	(0.859)	1.62	4.36E-10
Cetn2	26370	centrin 2	(0.860)	4.69	2.14E-06
Mgme1	74528	mitochondrial genome maintainance exonuclease 1	(0.861)	2.71	1.55E-09
Tbl3	213773	transducin (beta)-like 3	(0.861)	8.75	2.74E-23
Rmnd5b	66089	required for meiotic nuclear division 5 homolog B (S. cerevisiae)	(0.862)	3.75	8.09E-09
L3mbtl3	237339	l(3)mbt-like 3 (Drosophila)	(0.862)	1.96	1.41E-12
Mcrs1	51812	microspherule protein 1	(0.862)	10.37	1.03E-21
Smim8	66291	small integral membrane protein 8	(0.864)	4.52	8.42E-06
D7Ertd128e	52222	-	(0.865)	1.95	1.25E-10
Aagab	66939	alpha- and gamma-adaptin binding protein	(0.867)	1.90	2.80E-06
Klc4	74764	kinesin light chain 4	(0.868)	2.08	2.57E-07
Zfp59	22717	zinc finger protein 59	(0.869)	1.46	1.33E-09
Wdr81	192652	WD repeat domain 81	(0.870)	3.13	2.59E-23

Official Gene Symbol	Entrez Gene ID	Gene description	Normalized Fold Change LOG2(Cryo-2 / Fresh-1)	FPKM Mean of six samples	p-value of Cryo-2 vs Fresh-1
Cdc7	12545	cell division cycle 7 (S. cerevisiae)	(0.870)	3.32	3.53E-13
Hpcal1	53602	hippocalcin-like 1	(0.871)	1.73	2.58E-06
Fndc3b	72007	fibronectin type III domain containing 3B	(0.871)	1.04	9.45E-10
Ap1ar	211556	adaptor-related protein complex 1 associated regulatory protein	(0.871)	6.64	7.82E-17
Mapk1ip1	69546	mitogen-activated protein kinase 1 interacting protein 1	(0.872)	2.73	1.08E-05
Gas8	104346	growth arrest specific 8	(0.872)	2.38	2.68E-05
Trim43b	666747	tripartite motif-containing 43B	(0.872)	5.23	5.09E-13
Pitx1	18740	paired-like homeodomain transcription factor 1	(0.873)	2.11	5.24E-07
Epha1	13835	Eph receptor A1	(0.873)	1.02	1.17E-04
Kif21a	16564	kinesin family member 21A	(0.874)	0.75	1.60E-06
Ppif	105675	peptidylprolyl isomerase F (cyclophilin F)	(0.874)	7.99	5.75E-15
Rgs14	51791	regulator of G-protein signaling 14	(0.875)	0.73	8.64E-03
Fam188a	66960	family with sequence similarity 188, member A	(0.875)	1.19	1.35E-09
Dmtf1	23857	cyclin D binding myb-like transcription factor 1	(0.875)	8.70	2.45E-40
Churc1	211151	churchill domain containing 1	(0.877)	10.60	2.87E-07
Fbxo3	57443	F-box protein 3	(0.878)	4.34	1.61E-22
Arrdc3	105171	arrestin domain containing 3	(0.879)	0.95	2.30E-06
Hist3h2a	319162	histone cluster 3, H2a	(0.879)	82.80	3.19E-42
Wdr72	546144	WD repeat domain 72	(0.879)	1.10	8.49E-08
Mob3a	208228	MOB kinase activator 3A	(0.879)	2.09	3.51E-06
Lgr4	107515	leucine-rich repeat-containing G protein-coupled receptor 4	(0.880)	2.00	7.67E-11
Fam117a	215512	family with sequence similarity 117, member A	(0.881)	2.19	1.01E-16
Tmem170b	621976	transmembrane protein 170B	(0.881)	0.80	6.00E-07
Rrp36	224823	ribosomal RNA processing 36 homolog (S. cerevisiae)	(0.883)	4.80	8.75E-09
Wdsub1	72137	WD repeat, SAM and U-box domain containing 1	(0.883)	1.33	1.46E-04
Arsa	11883	arylsulfatase A	(0.883)	0.87	1.32E-04
Sclt1	67161	sodium channel and clathrin linker 1	(0.885)	1.76	5.99E-06
Vars2	68915	valyl-tRNA synthetase 2, mitochondrial (putative)	(0.888)	1.79	2.94E-10
Rpl21-ps15	675278	ribosomal protein L21, pseudogene 15	(0.888)	1.93	6.31E-29
Parp16	214424	poly (ADP-ribose) polymerase family, member 16	(0.890)	0.73	3.25E-03
Cd97	26364	CD97 antigen	(0.892)	5.42	5.19E-21
Trpc5os	100503240	transient receptor potential cation channel, subfamily C, member 5, opposite strand	(0.892)	0.90	5.85E-06
Eif2s3y	26908	eukaryotic translation initiation factor 2, subunit 3, structural gene Y-linked	(0.892)	7.81	1.65E-24
Golim4	73124	golgi integral membrane protein 4	(0.894)	0.56	6.10E-04
Ankhd1	108857	ankyrin repeat and KH domain containing 1	(0.895)	6.11	2.98E-52
Pet100	100503890	PET100 homolog (S. cerevisiae)	(0.895)	11.21	3.47E-16
Acsl1	14081	acyl-CoA synthetase long-chain family member 1	(0.896)	0.87	1.20E-05
Ptrh1	329384	peptidyl-tRNA hydrolase 1 homolog (S. cerevisiae)	(0.896)	2.12	5.29E-03
Rbm4	19653	RNA binding motif protein 4	(0.897)	3.44	4.28E-10
Cdc42ep4	56699	CDC42 effector protein (Rho GTPase binding) 4	(0.897)	1.42	1.80E-08
Tmem43	74122	transmembrane protein 43	(0.900)	4.24	1.20E-14
Slc33a1	11416	solute carrier family 33 (acetyl-CoA transporter), member 1	(0.900)	5.53	2.15E-17
Alg11	207958	asparagine-linked glycosylation 11 (alpha-1,2-mannosyltransferase)	(0.900)	1.93	1.23E-12
Laptm5	16792	lysosomal-associated protein transmembrane 5	(0.901)	1.65	8.39E-06

Official Gene Symbol	Entrez Gene ID	Gene description	Normalized Fold Change LOG2(Cryo-2 / Fresh-1)	FPKM Mean of six samples	p-value of Cryo-2 vs Fresh-1
Creb3	12913	cAMP responsive element binding protein 3	(0.904)	0.82	2.46E-02
Nacc2	67991	nucleus accumbens associated 2, BEN and BTB (POZ) domain containing	(0.905)	0.99	5.18E-09
Xaf1	327959	XIAP associated factor 1	(0.905)	0.58	2.00E-03
Galnt6	207839	UDP-N-acetyl-alpha-D-galactosamine:polypeptide N-acetylgalactosaminyltransferase 6	(0.906)	1.85	8.32E-14
Tmem161b	72745	transmembrane protein 161B	(0.906)	4.55	2.16E-20
Zrsr1	22183	zinc finger (CCCH type), RNA binding motif and serine/arginine rich 1	(0.907)	2.34	5.32E-12
Gpa33	59290	glycoprotein A33 (transmembrane)	(0.907)	2.06	1.13E-06
Fh1	14194	fumarate hydratase 1	(0.908)	9.67	3.02E-17
Hspbap1	66667	Hspb associated protein 1	(0.908)	3.57	4.56E-26
Rad51ap1	19362	RAD51 associated protein 1	(0.908)	4.90	3.37E-11
Cds1	74596	CDP-diacylglycerol synthase 1	(0.909)	1.41	7.76E-07
Vps45	22365	vacuolar protein sorting 45 (yeast)	(0.909)	0.80	2.32E-07
Spice1	212514	spindle and centriole associated protein 1	(0.911)	1.51	1.04E-08
Qdpr	110391	quinoid dihydropteridine reductase	(0.912)	3.68	7.35E-07
Aaed1	66129	AhpC/TSA antioxidant enzyme domain containing 1	(0.912)	5.27	8.68E-07
Magea5	17141	melanoma antigen, family A, 5	(0.912)	3.98	3.92E-08
Cnppd1	69171	cyclin Pas1/PHO80 domain containing 1	(0.912)	3.06	5.12E-13
Zufsp	72580	zinc finger with UFM1-specific peptidase domain	(0.913)	4.39	1.60E-16
Piga	18700	phosphatidylinositol glycan anchor biosynthesis, class A	(0.914)	1.53	1.83E-07
Mir17hg	75957	Mir17 host gene (non-protein coding)	(0.916)	4.47	1.21E-13
Nkrf	77286	NF-kappaB repressing factor	(0.918)	2.01	1.55E-10
Supt3	109115	suppressor of Ty 3	(0.918)	1.40	8.72E-08
Endog	13804	endonuclease G	(0.919)	2.69	3.59E-04
Atg16l2	73683	autophagy related 16-like 2 (S. cerevisiae)	(0.921)	2.01	1.09E-15
Gtdc1	227835	glycosyltransferase-like domain containing 1	(0.921)	1.79	1.63E-08
Glt1d1	319804	glycosyltransferase 1 domain containing 1	(0.922)	0.54	5.90E-03
Alg8	381903	asparagine-linked glycosylation 8 (alpha-1,3-glucosyltransferase)	(0.923)	10.22	9.48E-25
Lyrm5	67636	LYR motif containing 5	(0.924)	3.84	4.09E-10
Irgq	210146	immunity-related GTPase family, Q	(0.924)	2.15	8.79E-17
Fancl	67030	Fanconi anemia, complementation group L	(0.925)	2.82	3.73E-07
Zscan21	22697	zinc finger and SCAN domain containing 21	(0.925)	4.49	1.52E-13
Dhrs7b	216820	dehydrogenase/reductase (SDR family) member 7B	(0.926)	1.47	5.23E-04
Cd28	12487	CD28 antigen	(0.927)	0.87	7.76E-06
Birc3	11796	baculoviral IAP repeat-containing 3	(0.928)	1.56	2.83E-11
Atrip	235610	ATR interacting protein	(0.928)	0.84	1.41E-05
Gstcd	67553	glutathione S-transferase, C-terminal domain containing	(0.928)	3.22	5.33E-16
Rgmb	68799	repulsive guidance molecule family member B	(0.928)	0.65	8.87E-05
Zfp444	72667	zinc finger protein 444	(0.928)	0.95	4.34E-06
Fam169b	434197	family with sequence similarity 169, member B	(0.929)	0.60	2.57E-03
Klf7	93691	Kruppel-like factor 7 (ubiquitous)	(0.930)	0.74	3.06E-04
Tcaim	382117	T cell activation inhibitor, mitochondrial	(0.931)	1.89	9.58E-10
Mif4gd	69674	MIF4G domain containing	(0.932)	3.90	4.06E-10
Uty	22290	ubiquitously transcribed tetratricopeptide repeat gene, Y chromosome	(0.933)	1.60	4.39E-12
Zmym6	100177	zinc finger, MYM-type 6	(0.935)	1.34	1.70E-09
Elp6	72341	elongator acetyltransferase complex subunit 6	(0.937)	1.69	4.06E-06

Official Gene Symbol	Entrez Gene ID	Gene description	Normalized Fold Change LOG2(Cryo-2 / Fresh-1)	FPKM Mean of six samples	p-value of Cryo-2 vs Fresh-1
Tmem167b	67495	transmembrane protein 167B	(0.938)	2.75	6.49E-11
Tnfrsf8	21941	tumor necrosis factor receptor superfamily, member 8	(0.938)	0.65	3.75E-02
Slc37a1	224674	solute carrier family 37 (glycerol-3-phosphate transporter), member 1	(0.939)	0.86	1.30E-06
Cyb5b	66427	cytochrome b5 type B	(0.940)	3.42	5.69E-18
Tmem14c	66154	transmembrane protein 14C	(0.940)	2.71	1.74E-04
Desi1	28075	desumoylating isopeptidase 1	(0.940)	3.17	1.22E-10
Gss	14854	glutathione synthetase	(0.940)	15.66	6.42E-36
Zbtb9	474156	zinc finger and BTB domain containing 9	(0.943)	2.09	1.27E-08
Pex5	19305	peroxisomal biogenesis factor 5	(0.943)	2.63	2.77E-11
Clpb	20480	ClpB caseinolytic peptidase B	(0.943)	3.16	8.87E-20
Zfat	380993	zinc finger and AT hook domain containing	(0.944)	0.75	1.94E-07
Osbpl6	99031	oxysterol binding protein-like 6	(0.945)	2.82	1.90E-28
Dvl1	13542	dishevelled, dsh homolog 1 (Drosophila)	(0.947)	0.92	3.42E-05
Pan2	103135	PAN2 polyA specific ribonuclease subunit homolog (S. cerevisiae)	(0.948)	4.83	3.19E-23
Birc2	11797	baculoviral IAP repeat-containing 2	(0.950)	3.16	9.62E-16
Trmt2a	15547	TRM2 tRNA methyltransferase 2A	(0.950)	3.67	1.14E-17
Fcgr3	14131	Fc receptor, IgG, low affinity III	(0.951)	1.34	5.83E-04
Rpp14	67053	ribonuclease P 14 subunit	(0.952)	6.83	2.78E-24
Rassf7	66985	Ras association (RalGDS/AF-6) domain family (N-terminal) member 7	(0.952)	5.91	9.03E-15
Ccdc73	211936	coiled-coil domain containing 73	(0.953)	0.71	1.21E-04
Etv6	14011	ets variant 6	(0.954)	0.88	4.10E-09
Pus3	67049	pseudouridine synthase 3	(0.955)	3.46	5.22E-11
Cpne1	266692	copine I	(0.955)	2.67	1.91E-13
Rwdd2b	53858	RWD domain containing 2B	(0.955)	1.26	2.51E-04
Ctnnbl1	66642	catenin, beta like 1	(0.957)	5.81	4.97E-19
Plekhm3	241075	pleckstrin homology domain containing, family M, member 3	(0.958)	1.96	2.46E-22
Sertad2	58172	SERTA domain containing 2	(0.960)	1.43	1.27E-10
Cyp4f16	70101	cytochrome P450, family 4, subfamily f, polypeptide 16	(0.961)	0.64	9.96E-03
Ap3b2	11775	adaptor-related protein complex 3, beta 2 subunit	(0.962)	0.74	6.10E-05
Lig4	319583	ligase IV, DNA, ATP-dependent	(0.962)	1.48	5.86E-09
Hagh	14651	hydroxyacyl glutathione hydrolase	(0.963)	1.77	1.33E-04
Fgf10	14165	fibroblast growth factor 10	(0.964)	1.83	5.52E-12
LOC105244431	105244431	-	(0.965)	0.58	1.50E-09
Klhl22	224023	kelch-like 22	(0.969)	4.77	4.28E-24
Gnptg	214505	N-acetylglucosamine-1-phosphotransferase, gamma subunit	(0.970)	5.06	3.93E-10
Vars	22321	valyl-tRNA synthetase	(0.970)	8.70	1.03E-45
Rpl14-ps1	100040970	ribosomal protein L14, pseudogene 1	(0.970)	25.29	1.12E-04
Acad11	102632	acyl-Coenzyme A dehydrogenase family, member 11	(0.971)	2.20	5.08E-11
Zfyve21	68520	zinc finger, FYVE domain containing 21	(0.971)	4.55	8.04E-10
Marveld2	218518	MARVEL (membrane-associating) domain containing 2	(0.971)	1.26	3.11E-06
Hesx1	15209	homeobox gene expressed in ES cells	(0.974)	0.84	3.64E-02
Mtfmt	69606	mitochondrial methionyl-tRNA formyltransferase	(0.975)	2.90	8.99E-14
Epm2aip1	77781	EPM2A (laforin) interacting protein 1	(0.978)	1.05	2.21E-11

Official Gene Symbol	Entrez Gene ID	Gene description	Normalized Fold Change LOG2(Cryo-2 / Fresh-1)	FPKM Mean of six samples	p-value of Cryo-2 vs Fresh-1
N6amt1	67768	N-6 adenine-specific DNA methyltransferase 1 (putative)	(0.978)	2.56	6.47E-07
Zfp13	22654	zinc finger protein 13	(0.981)	1.25	2.60E-05
Kdm1b	218214	lysine (K)-specific demethylase 1B	(0.981)	1.41	2.73E-09
Ccdc181	74895	coiled-coil domain containing 181	(0.982)	0.80	6.39E-03
Adprm	66358	ADP-ribose/CDP-alcohol diphosphatase, manganese dependent	(0.983)	2.20	2.19E-05
Timm21	67105	tranlocase of inner mitochondrial membrane 21	(0.984)	0.73	1.30E-03
Atg4c	242557	autophagy related 4C, cysteine peptidase	(0.984)	0.77	3.95E-07
Ticam1	106759	toll-like receptor adaptor molecule 1	(0.984)	1.94	1.61E-08
Gpr125	70693	G protein-coupled receptor 125	(0.984)	0.89	1.69E-05
Pigyl	66268	phosphatidylinositol glycan anchor biosynthesis, class Y-like	(0.985)	11.85	1.47E-12
Phf20	228829	PHD finger protein 20	(0.985)	2.69	2.16E-18
Srek1ip1	67288	splicing regulatory glutamine/lysine-rich protein 1interacting protein 1	(0.986)	3.99	5.78E-09
Rsl1	380855	regulator of sex limited protein 1	(0.986)	1.59	4.59E-05
Hmgcr	15357	3-hydroxy-3-methylglutaryl-Coenzyme A reductase	(0.986)	6.60	2.27E-41
Tmed1	17083	transmembrane emp24 domain containing 1	(0.987)	2.87	5.72E-07
Sh3tc2	225608	SH3 domain and tetratricopeptide repeats 2	(0.989)	1.41	1.99E-12
Inpp5f	101490	inositol polyphosphate-5-phosphatase F	(0.990)	1.06	3.33E-09
Dph1	116905	DPH1 homolog (S. cerevisiae)	(0.993)	2.69	1.42E-08
Eef2kmt	70511	eukaryotic elongation factor 2 lysine methyltransferase	(0.994)	3.16	3.18E-10
Mettl14	210529	methyltransferase like 14	(0.995)	5.16	2.30E-15
Snapc3	77634	small nuclear RNA activating complex, polypeptide 3	(0.997)	1.47	2.38E-07
Tfeb	21425	transcription factor EB	(0.998)	6.21	8.27E-28
Cbx2	12416	chromobox 2	(0.999)	0.60	4.82E-06
LOC105246429	105246429	-	(1.000)	0.87	3.69E-05
Alg10b	380959	asparagine-linked glycosylation 10B (alpha-1,2-glucosyltransferase)	(1.002)	4.91	7.97E-26
Zfp935	71508	zinc finger protein 935	(1.002)	7.73	7.79E-24
Zfp524	66056	zinc finger protein 524	(1.004)	0.93	3.86E-03
Deaf1	54006	deformed epidermal autoregulatory factor 1 (Drosophila)	(1.005)	1.69	6.95E-13
Chchd4	72170	coiled-coil-helix-coiled-coil-helix domain containing 4	(1.006)	6.59	4.63E-12
Socs6	54607	suppressor of cytokine signaling 6	(1.007)	2.94	1.15E-24
Klf11	194655	Kruppel-like factor 11	(1.007)	1.13	1.00E-06
Golga1	76899	golgi autoantigen, golgin subfamily a, 1	(1.007)	2.87	6.51E-21
Arel1	68497	apoptosis resistant E3 ubiquitin protein ligase 1	(1.009)	2.09	4.37E-17
Apip	56369	APAF1 interacting protein	(1.010)	3.27	3.01E-06
Sec14l1	74136	SEC14-like 1 (S. cerevisiae)	(1.010)	1.99	2.05E-14
Xylt2	217119	xylosyltransferase II	(1.011)	1.42	1.15E-07
Trub2	227682	TruB pseudouridine (psi) synthase homolog 2 (E. coli)	(1.011)	2.11	1.56E-15
Miip	28010	migration and invasion inhibitory protein	(1.011)	5.03	1.49E-13
Nsun3	106338	NOL1/NOP2/Sun domain family member 3	(1.014)	1.97	6.52E-12
Aplf	72103	aprataxin and PNKP like factor	(1.015)	1.23	1.80E-07
Idi1	319554	isopentenyl-diphosphate delta isomerase	(1.016)	6.27	3.84E-18
Avpi1	69534	arginine vasopressin-induced 1	(1.017)	8.46	2.45E-13
Gyk	14933	glycerol kinase	(1.018)	3.23	1.56E-16

Official Gene Symbol	Entrez Gene ID	Gene description	Normalized Fold Change LOG2(Cryo-2 / Fresh-1)	FPKM Mean of six samples	p-value of Cryo-2 vs Fresh-1
Slc37a2	56857	solute carrier family 37 (glycerol-3-phosphate transporter), member 2	(1.018)	2.10	1.58E-14
Irf2bp1	272359	interferon regulatory factor 2 binding protein 1	(1.019)	2.63	1.76E-11
Dak	225913	dihydroxyacetone kinase 2 homolog (yeast)	(1.023)	1.23	3.23E-06
Sdcbp2	228765	syndecan binding protein (syntenin) 2	(1.025)	0.64	2.15E-02
Wdr92	103784	WD repeat domain 92	(1.025)	2.97	1.42E-12
Gdf3	14562	growth differentiation factor 3	(1.027)	4.28	4.63E-15
Slc37a3	72144	solute carrier family 37 (glycerol-3-phosphate transporter), member 3	(1.027)	2.47	3.05E-16
Def8	23854	differentially expressed in FDCP 8	(1.028)	3.55	5.91E-27
Bin3	57784	bridging integrator 3	(1.029)	0.66	1.50E-04
Mogat2	233549	monoacylglycerol O-acyltransferase 2	(1.030)	1.33	3.46E-04
Snapc2	102209	small nuclear RNA activating complex, polypeptide 2	(1.032)	5.30	6.08E-14
Dgcr6	13353	DiGeorge syndrome critical region gene 6	(1.033)	0.79	2.08E-03
Dus3l	224907	dihydrouridine synthase 3-like (S. cerevisiae)	(1.034)	4.87	6.26E-18
LOC105245566	105245566	-	(1.034)	0.92	2.70E-02
C2cd3	277939	C2 calcium-dependent domain containing 3	(1.036)	1.15	2.06E-13
Casp9	12371	caspase 9	(1.037)	1.16	7.35E-08
Cluh	74148	clustered mitochondria (cluA/CLU1) homolog	(1.037)	8.40	1.23E-57
Dusp19	68082	dual specificity phosphatase 19	(1.038)	2.01	3.61E-05
Kbtbd13	74492	kelch repeat and BTB (POZ) domain containing 13	(1.039)	1.51	6.55E-07
Znf512b	269401	zinc finger protein 512B	(1.039)	0.71	1.49E-06
Cdc42ep1	104445	CDC42 effector protein (Rho GTPase binding) 1	(1.042)	2.17	4.08E-09
Pias3	229615	protein inhibitor of activated STAT 3	(1.042)	0.71	1.54E-04
Slc27a1	26457	solute carrier family 27 (fatty acid transporter), member 1	(1.044)	1.11	4.18E-08
BC048679	210321	cDNA sequence BC048679	(1.045)	4.78	5.01E-06
Pigl	327942	phosphatidylinositol glycan anchor biosynthesis, class L	(1.046)	2.27	1.78E-06
Zfp748	212276	zinc finger protein 748	(1.047)	2.76	2.99E-18
Pcgf2	22658	polycomb group ring finger 2	(1.047)	1.31	1.00E-08
Hist1h2be	319179	histone cluster 1, H2be	(1.048)	6.74	2.55E-25
Rps6kb2	58988	ribosomal protein S6 kinase, polypeptide 2	(1.048)	3.53	7.06E-11
Adprhl2	100206	ADP-ribosylhydrolase like 2	(1.048)	2.95	3.22E-07
Zfp954	232853	zinc finger protein 954	(1.049)	1.41	2.02E-05
Arhgap18	73910	Rho GTPase activating protein 18	(1.049)	2.04	1.90E-14
Smarcd3	66993	SWI/SNF related, matrix associated, actin dependent regulator of chromatin, subfamily d, member 3	(1.053)	0.61	2.17E-03
Mtmr2	77116	myotubularin related protein 2	(1.057)	4.12	6.92E-36
Tmem50a	71817	transmembrane protein 50A	(1.057)	9.64	2.16E-19
Edc3	353190	enhancer of mRNA decapping 3 homolog (S. cerevisiae)	(1.057)	1.36	6.69E-08
Xpc	22591	xeroderma pigmentosum, complementation group C	(1.059)	3.12	9.25E-18
Fabp3-ps1	14074	fatty acid binding protein 3, muscle and heart, pseudogene 1	(1.060)	22.86	2.12E-22
Mon1a	72825	MON1 homolog A (yeast)	(1.060)	4.36	3.93E-14
Zfp362	230761	zinc finger protein 362	(1.062)	0.82	1.37E-04
Plxnd1	67784	plexin D1	(1.062)	0.78	9.88E-09
Mus81	71711	MUS81 endonuclease homolog (yeast)	(1.063)	1.43	1.19E-05
Mrps36	66128	mitochondrial ribosomal protein S36	(1.063)	9.10	1.56E-10

Official Gene Symbol	Entrez Gene ID	Gene description	Normalized Fold Change LOG2(Cryo-2 / Fresh-1)	FPKM Mean of six samples	p-value of Cryo-2 vs Fresh-1
Adat3	100113398	adenosine deaminase, tRNA-specific 3	(1.063)	1.55	4.99E-04
Cntrob	216846	centrobin, centrosomal BRCA2 interacting protein	(1.063)	1.60	1.21E-12
Arsk	77041	arylsulfatase K	(1.064)	1.01	1.16E-06
Eif4a-ps4	13674	eukaryotic translation initiation factor 4A, pseudogene 4	(1.064)	2.29	2.24E-07
Fam195b	192173	family with sequence similarity 195, member B	(1.066)	0.57	4.30E-02
Rab3gap2	98732	RAB3 GTPase activating protein subunit 2	(1.067)	2.11	2.01E-24
Sptbn2	20743	spectrin beta, non-erythrocytic 2	(1.069)	2.97	1.15E-41
Tcf3	21423	transcription factor 3	(1.069)	2.25	1.97E-13
Chid1	68038	chitinase domain containing 1	(1.070)	2.04	1.19E-14
Nmral1	67824	NmrA-like family domain containing 1	(1.070)	1.63	1.94E-05
Acyp1	66204	acylphosphatase 1, erythrocyte (common) type	(1.072)	1.52	5.08E-03
Nthl1	18207	nth (endonuclease III)-like 1 (E.coli)	(1.073)	1.62	1.33E-05
Smim12	80284	small integral membrane protein 12	(1.073)	1.49	5.14E-03
Dnajc4	57431	DnaJ (Hsp40) homolog, subfamily C, member 4	(1.076)	2.00	1.97E-05
C1qtnf4	67445	C1q and tumor necrosis factor related protein 4	(1.077)	0.92	1.81E-06
Pik3r2	18709	phosphatidylinositol 3-kinase, regulatory subunit, polypeptide 2 (p85 beta)	(1.077)	3.92	7.43E-22
Sec13	110379	SEC13 homolog (S. cerevisiae)	(1.082)	29.76	2.15E-58
Hsd17b7	15490	hydroxysteroid (17-beta) dehydrogenase 7	(1.083)	0.60	2.13E-06
Dgcr2	13356	DiGeorge syndrome critical region gene 2	(1.085)	3.10	3.88E-22
Glod4	67201	glyoxalase domain containing 4	(1.086)	6.64	2.02E-33
Sec14l4	103655	SEC14-like 4 (S. cerevisiae)	(1.088)	2.41	6.93E-16
Gpam	14732	glycerol-3-phosphate acyltransferase, mitochondrial	(1.088)	0.92	5.61E-11
Mto1	68291	mitochondrial translation optimization 1 homolog (S. cerevisiae)	(1.089)	3.71	6.38E-13
Ndufaf4	68493	NADH dehydrogenase (ubiquinone) 1 alpha subcomplex, assembly factor 4	(1.090)	3.16	2.65E-19
Rabgap1	227800	RAB GTPase activating protein 1	(1.092)	3.19	1.38E-38
Map7d1	245877	MAP7 domain containing 1	(1.092)	0.94	3.05E-06
Nipsnap1	18082	4-nitrophenylphosphatase domain and non-neuronal SNAP25-like protein homolog 1 (C. elegans)	(1.093)	1.89	4.26E-08
Dis3l2	208718	DIS3 mitotic control homolog (S. cerevisiae)-like 2	(1.094)	1.43	1.48E-08
Stk25	59041	serine/threonine kinase 25 (yeast)	(1.095)	5.21	2.75E-31
Gatsl3	71962	GATS protein-like 3	(1.096)	4.68	2.49E-13
Arl16	70317	ADP-ribosylation factor-like 16	(1.096)	0.64	6.65E-03
Gtpbp3	70359	GTP binding protein 3	(1.098)	1.09	1.48E-06
Ift46	76568	intraflagellar transport 46	(1.099)	2.51	1.05E-16
Dok4	114255	docking protein 4	(1.101)	1.42	3.12E-07
Osbp	76303	oxysterol binding protein	(1.102)	3.21	8.36E-35
Ctu2	66965	cytosolic thiouridylase subunit 2 homolog (S. pombe)	(1.102)	2.33	4.43E-12
Ly6g6e	70274	lymphocyte antigen 6 complex, locus G6E	(1.109)	1.45	3.11E-04
Slc25a19	67283	solute carrier family 25 (mitochondrial thiamine pyrophosphate carrier), member 19	(1.109)	1.63	4.34E-09
Ccdc22	54638	coiled-coil domain containing 22	(1.110)	3.10	1.57E-12
Rab13	68328	RAB13, member RAS oncogene family	(1.110)	1.04	1.13E-03
Aaas	223921	achalasia, adrenocortical insufficiency, alacrimia	(1.113)	14.66	7.56E-42
Stim1	20866	stromal interaction molecule 1	(1.117)	1.12	3.98E-10
Taf1b	21340	TATA box binding protein (Tbp)-associated factor, RNA polymerase I, B	(1.117)	4.88	1.75E-20
Haus5	71909	HAUS augmin-like complex, subunit 5	(1.119)	4.95	1.49E-19

Official Gene Symbol	Entrez Gene ID	Gene description	Normalized Fold Change LOG2(Cryo-2 / Fresh-1)	FPKM Mean of six samples	p-value of Cryo-2 vs Fresh-1
Spg7	234847	spastic paraplegia 7 homolog (human)	(1.121)	3.23	2.60E-13
Cyb5r1	72017	cytochrome b5 reductase 1	(1.121)	8.39	3.76E-24
Zfp275	27081	zinc finger protein 275	(1.123)	1.00	9.63E-12
Lyrm4	380840	LYR motif containing 4	(1.123)	4.95	1.43E-13
Tada2b	231151	transcriptional adaptor 2B	(1.124)	1.78	3.00E-12
Gtsf1	74174	gametocyte specific factor 1	(1.125)	1.26	4.28E-03
Coq10b	67876	coenzyme Q10 homolog B (S. cerevisiae)	(1.129)	4.49	1.85E-11
Rab31	106572	RAB31, member RAS oncogene family	(1.133)	1.68	6.64E-11
Nckipsd	80987	NCK interacting protein with SH3 domain	(1.136)	0.56	1.72E-04
Mlkl	74568	mixed lineage kinase domain-like	(1.136)	1.34	3.81E-06
Ipp	16351	IAP promoted placental gene	(1.137)	1.23	1.66E-06
Ints5	109077	integrator complex subunit 5	(1.138)	3.20	7.59E-17
Ttc30b	72421	tetratricopeptide repeat domain 30B	(1.138)	0.87	1.02E-04
Acy1	109652	aminoacylase 1	(1.139)	0.73	9.77E-03
Vcpkmt	207965	valosin containing protein lysine (K) methyltransferase	(1.141)	1.05	7.60E-05
Tars2	71807	threonyl-tRNA synthetase 2, mitochondrial (putative)	(1.141)	3.07	1.33E-21
Dguok	27369	deoxyguanosine kinase	(1.144)	1.64	7.52E-06
Ppp1r13b	21981	protein phosphatase 1, regulatory (inhibitor) subunit 13B	(1.145)	1.20	2.15E-09
Snx19	102607	sorting nexin 19	(1.145)	0.68	3.64E-10
Mafa	378435	v-maf musculoaponeurotic fibrosarcoma oncogene family, protein A (avian)	(1.147)	1.18	3.84E-03
Gnb2	14693	guanine nucleotide binding protein (G protein), beta 2	(1.149)	2.09	2.92E-11
Usp11	236733	ubiquitin specific peptidase 11	(1.149)	0.75	1.99E-04
Fancd2os	70979	Fancd2 opposite strand	(1.153)	0.57	2.66E-02
Tcirg1	27060	T cell, immune regulator 1, ATPase, H+ transporting, lysosomal V0 protein A3	(1.153)	0.86	1.02E-05
Ell3	269344	elongation factor RNA polymerase II-like 3	(1.154)	2.56	4.69E-10
Ubiad1	71707	UbiA prenyltransferase domain containing 1	(1.160)	1.07	1.04E-06
Eif4ebp3	108112	eukaryotic translation initiation factor 4E binding protein 3	(1.163)	0.64	3.25E-02
Nsl1	381318	NSL1, MIND kinetochore complex component, homolog (S. cerevisiae)	(1.164)	1.88	1.54E-13
Pyroxd1	232491	pyridine nucleotide-disulphide oxidoreductase domain 1	(1.165)	2.41	2.41E-13
Thap3	69876	THAP domain containing, apoptosis associated protein 3	(1.166)	1.21	2.26E-03
LOC102639021	102639021	-	(1.166)	2.41	3.45E-04
Zfp956	101197	zinc finger protein 956	(1.170)	0.61	6.16E-04
Prom1	19126	prominin 1	(1.170)	0.84	1.25E-09
Dock10	210293	dedicator of cytokinesis 10	(1.172)	0.73	3.20E-13
Usp21	30941	ubiquitin specific peptidase 21	(1.172)	1.17	2.53E-04
Zswim8	268721	zinc finger SWIM-type containing 8	(1.173)	0.98	1.65E-12
Lif	16878	leukemia inhibitory factor	(1.173)	0.65	1.27E-08
Zfp329	67230	zinc finger protein 329	(1.175)	0.86	3.26E-14
Oplah	75475	5-oxoprolinase (ATP-hydrolysing)	(1.178)	0.96	1.03E-08
LOC105244426	105244426	-	(1.180)	1.39	4.80E-02
Bcdin3d	75284	BCDIN3 domain containing	(1.181)	1.30	9.10E-04

Official Gene Symbol	Entrez Gene ID	Gene description	Normalized Fold Change LOG2(Cryo-2 / Fresh-1)	FPKM Mean of six samples	p-value of Cryo-2 vs Fresh-1
Plekhg5	269608	pleckstrin homology domain containing, family G (with RhoGef domain) member 5	(1.181)	0.70	3.50E-07
Arfgap1	228998	ADP-ribosylation factor GTPase activating protein 1	(1.184)	5.50	2.13E-31
Decr1	67460	2,4-dienoyl CoA reductase 1, mitochondrial	(1.184)	1.62	5.87E-09
Dbr1	83703	debranching enzyme homolog 1 (S. cerevisiae)	(1.186)	4.60	3.67E-20
Ppp2r4	110854	protein phosphatase 2A activator, regulatory subunit B	(1.187)	3.96	1.57E-17
Nek8	140859	NIMA (never in mitosis gene a)-related expressed kinase 8	(1.188)	0.81	9.26E-06
Pi4kb	107650	phosphatidylinositol 4-kinase, catalytic, beta polypeptide	(1.188)	3.43	3.11E-25
Fbxo31	76454	F-box protein 31	(1.191)	2.34	5.48E-21
Mtrr	210009	5-methyltetrahydrofolate-homocysteine methyltransferase reductase	(1.192)	6.79	1.37E-55
Polr3d	67065	polymerase (RNA) III (DNA directed) polypeptide D	(1.192)	3.60	1.30E-14
Lmod3	320502	leiomodin 3 (fetal)	(1.192)	0.82	1.88E-04
Aim1	11630	absent in melanoma 1	(1.195)	1.61	4.21E-19
Dph5	69740	DPH5 homolog (S. cerevisiae)	(1.202)	2.83	2.31E-08
Depdc1b	218581	DEP domain containing 1B	(1.202)	0.88	1.86E-06
Mrps6	121022	mitochondrial ribosomal protein S6	(1.203)	10.92	1.93E-16
Slc38a11	320106	solute carrier family 38, member 11	(1.204)	1.48	4.06E-09
Mocs2	17434	molybdenum cofactor synthesis 2	(1.205)	2.10	2.09E-07
Hyls1	76832	hydrolethalus syndrome 1	(1.205)	2.63	3.11E-09
Alkbh4	72041	alkB, alkylation repair homolog 4 (E. coli)	(1.211)	1.50	5.66E-07
Trappc2	66226	trafficking protein particle complex 2	(1.214)	2.74	1.24E-04
Mettl13	71449	methyltransferase like 13	(1.215)	2.38	1.67E-13
Dhtkd1	209692	dehydrogenase E1 and transketolase domain containing 1	(1.215)	1.08	9.80E-09
Strip1	229707	striatin interacting protein 1	(1.216)	5.67	1.02E-33
Ext1	14042	exostoses (multiple) 1	(1.216)	1.40	1.71E-10
Rpl28-ps3	100039645	ribosomal protein L28, pseudogene 3	(1.218)	4.69	1.71E-05
Nit1	27045	nitrilase 1	(1.219)	6.38	4.39E-22
Nfkbil1	18038	nuclear factor of kappa light polypeptide gene enhancer in B cells inhibitor like 1	(1.220)	1.57	1.94E-05
Cnga3	12790	cyclic nucleotide gated channel alpha 3	(1.221)	0.53	9.66E-05
Clec2g	70809	C-type lectin domain family 2, member g	(1.223)	1.05	9.43E-06
Armc6	76813	armadillo repeat containing 6	(1.224)	1.09	3.69E-06
Pif1	208084	PIF1 5'-to-3' DNA helicase homolog (S. cerevisiae)	(1.228)	1.09	5.49E-10
Zfp868	234362	zinc finger protein 868	(1.230)	1.85	2.36E-11
Mael	98558	maelstrom homolog (Drosophila)	(1.231)	4.05	5.56E-11
Sars2	71984	seryl-aminoacyl-tRNA synthetase 2	(1.232)	3.64	9.92E-14
Phf11c	628705	PHD finger protein 11C	(1.234)	1.15	3.44E-17
Tmem189	407243	transmembrane protein 189	(1.235)	5.09	6.16E-18
Ccdc9	243846	coiled-coil domain containing 9	(1.236)	1.61	1.48E-10
Nsun5	100609	NOL1/NOP2/Sun domain family, member 5	(1.236)	2.74	3.18E-13
Ppp1r26	241289	protein phosphatase 1, regulatory subunit 26	(1.238)	1.56	1.82E-21
Hmg20a	66867	high mobility group 20A	(1.240)	2.37	3.03E-23
Tmtc2	278279	transmembrane and tetratricopeptide repeat containing 2	(1.243)	0.77	2.92E-09
Snip1	76793	Smad nuclear interacting protein 1	(1.247)	4.41	3.67E-21
Cobll1	319876	Cobl-like 1	(1.247)	0.87	2.18E-11
Eid2b	434156	EP300 interacting inhibitor of differentiation 2B	(1.249)	0.73	1.40E-04

Official Gene Symbol	Entrez Gene ID	Gene description	Normalized Fold Change LOG2(Cryo-2 / Fresh-1)	FPKM Mean of six samples	p-value of Cryo-2 vs Fresh-1
Myo18a	360013	myosin XVIIIA	(1.251)	0.77	2.45E-14
Eif4enif1	74203	eukaryotic translation initiation factor 4E nuclear import factor 1	(1.252)	6.14	4.94E-62
Pgm3	109785	phosphoglucomutase 3	(1.253)	2.16	1.02E-21
Pea15a	18611	phosphoprotein enriched in astrocytes 15A	(1.254)	1.41	1.14E-07
Yipf2	74766	Yip1 domain family, member 2	(1.261)	2.18	9.86E-11
Eftud1	101592	elongation factor Tu GTP binding domain containing 1	(1.261)	3.20	4.39E-22
Cphx3	100039280	-	(1.265)	0.83	1.55E-04
Hist2h2ab	621893	histone cluster 2, H2ab	(1.270)	3.85	2.41E-04
Tmem116	77462	transmembrane protein 116	(1.271)	0.78	1.31E-02
Zscan5b	170734	zinc finger and SCAN domain containing 5B	(1.272)	1.28	4.37E-06
Gphn	268566	gephyrin	(1.274)	0.56	7.31E-08
Tsen54	76265	tRNA splicing endonuclease 54 homolog (S. cerevisiae)	(1.275)	0.87	1.48E-05
Gtf2i	14886	general transcription factor II I	(1.277)	1.68	1.32E-19
Prkd2	101540	protein kinase D2	(1.277)	0.59	1.44E-05
Inpp5b	16330	inositol polyphosphate-5-phosphatase B	(1.278)	1.06	1.62E-11
D17H6S53E	114585	DNA segment, Chr 17, human D6S53E	(1.279)	0.56	4.75E-03
Bcl2l1	12048	BCL2-like 1	(1.279)	1.08	4.15E-10
Wash	68767	WAS protein family homolog	(1.280)	4.03	8.58E-25
Gadd45g	23882	growth arrest and DNA-damage-inducible 45 gamma	(1.281)	2.04	1.26E-05
LOC102635122	102635122	-	(1.281)	0.54	1.26E-02
Eif3j2	100042807	eukaryotic translation initiation factor 3, subunit J2	(1.281)	1.62	5.78E-08
Tasp1	75812	taspase, threonine aspartase 1	(1.284)	0.98	6.66E-12
Ago1	236511	argonaute RISC catalytic subunit 1	(1.284)	1.17	3.31E-19
Fam219b	78323	family with sequence similarity 219, member B	(1.285)	0.68	3.19E-05
Evl	14026	Ena-vasodilator stimulated phosphoprotein	(1.286)	0.82	1.02E-05
Pitpnc1	71795	phosphatidylinositol transfer protein, cytoplasmic 1	(1.286)	3.03	1.92E-43
Kdelc2	68304	KDEL (Lys-Asp-Glu-Leu) containing 2	(1.295)	0.81	2.73E-07
Slco4c1	227394	solute carrier organic anion transporter family, member 4C1	(1.296)	1.31	1.53E-07
Fn3krp	238024	fructosamine 3 kinase related protein	(1.297)	0.89	3.16E-07
Dgke	56077	diacylglycerol kinase, epsilon	(1.298)	2.02	7.32E-24
Rita1	100764	RBPJ interacting and tubulin associated 1	(1.298)	0.77	1.29E-03
Thra	21833	thyroid hormone receptor alpha	(1.298)	1.43	5.74E-08
Mapkapk3	102626	mitogen-activated protein kinase-activated protein kinase 3	(1.302)	1.63	1.77E-13
Gls2	216456	glutaminase 2 (liver, mitochondrial)	(1.304)	2.45	2.74E-15
LOC102641509	102641509	-	(1.304)	0.58	2.54E-08
Stk17b	98267	serine/threonine kinase 17b (apoptosis-inducing)	(1.306)	3.90	1.50E-26
Zfp771	244216	zinc finger protein 771	(1.307)	2.14	3.43E-07
Erlec1	66753	endoplasmic reticulum lectin 1	(1.308)	0.94	3.35E-15
Mettl3	56335	methyltransferase like 3	(1.309)	5.51	5.82E-25
Ciapin1	109006	cytokine induced apoptosis inhibitor 1	(1.315)	2.57	1.40E-26
Akr7a5	110198	aldo-keto reductase family 7, member A5 (aflatoxin aldehyde reductase)	(1.316)	3.01	1.05E-11
Ltbp4	108075	latent transforming growth factor beta binding protein 4	(1.319)	0.59	2.01E-08
Magea2	17138	melanoma antigen, family A, 2	(1.320)	1.20	1.93E-05
LOC105243212	105243212	-	(1.320)	1.37	1.24E-02
Lamb2	16779	laminin, beta 2	(1.325)	1.24	1.55E-15

Official Gene Symbol	Entrez Gene ID	Gene description	Normalized Fold Change LOG2(Cryo-2 / Fresh-1)	FPKM Mean of six samples	p-value of Cryo-2 vs Fresh-1
Pbx2	18515	pre B cell leukemia homeobox 2	(1.325)	4.47	2.54E-24
Tbccd1	70573	TBCC domain containing 1	(1.327)	2.29	1.46E-19
Slc35c2	228875	solute carrier family 35, member C2	(1.327)	1.83	9.11E-11
Srr	27364	serine racemase	(1.328)	1.81	2.81E-20
Lypd3	72434	Ly6/Plaur domain containing 3	(1.329)	0.53	1.00E-02
Nubp1	26425	nucleotide binding protein 1	(1.335)	5.83	2.78E-27
Polm	54125	polymerase (DNA directed), mu	(1.335)	1.68	3.65E-13
Kcnv2	240595	potassium channel, subfamily V, member 2	(1.335)	0.58	1.87E-08
Palb2	233826	partner and localizer of BRCA2	(1.337)	1.75	3.06E-14
Extl1	56219	exostoses (multiple)-like 1	(1.338)	0.82	2.20E-08
Ift172	67661	intraflagellar transport 172	(1.343)	1.63	1.06E-24
Neb	17996	nebulin	(1.348)	0.90	3.60E-47
Phkg2	68961	phosphorylase kinase, gamma 2 (testis)	(1.351)	2.59	1.86E-11
Dnajb3	15504	DnaJ (Hsp40) homolog, subfamily B, member 3	(1.353)	0.90	6.24E-03
Slc25a26	67582	solute carrier family 25 (mitochondrial carrier, phosphate carrier), member 26	(1.355)	1.71	2.18E-14
Ap5z1	231855	adaptor-related protein complex 5, zeta 1 subunit	(1.358)	1.03	6.46E-12
Fam193b	212483	family with sequence similarity 193, member B	(1.361)	1.86	2.95E-20
Taf6l	225895	TAF6-like RNA polymerase II, p300/CBP-associated factor (PCAF)-associated factor	(1.362)	1.11	2.19E-07
Adck1	72113	aarF domain containing kinase 1	(1.364)	1.11	8.50E-11
Cmklr1	14747	chemokine-like receptor 1	(1.367)	1.16	2.32E-10
Rhbdf2	217344	rhomboid 5 homolog 2 (Drosophila)	(1.368)	1.93	4.04E-19
BC051226	407803	cDNA sequence BC051226	(1.370)	0.95	5.04E-03
Rabep2	70314	rabaptin, RAB GTPase binding effector protein 2	(1.370)	2.20	3.69E-14
Ubox5	140629	U box domain containing 5	(1.371)	0.95	2.02E-09
Slc36a1	215335	solute carrier family 36 (proton/amino acid symporter), member 1	(1.375)	0.80	3.32E-11
Usb1	101985	U6 snRNA biogenesis 1	(1.375)	3.17	3.08E-14
Zfp623	78834	zinc finger protein 623	(1.375)	1.09	6.95E-08
Tnfaip8	106869	tumor necrosis factor, alpha-induced protein 8	(1.379)	2.06	4.10E-21
Sgsm1	52850	small G protein signaling modulator 1	(1.380)	0.57	1.93E-09
Vps37c	107305	vacuolar protein sorting 37C (yeast)	(1.380)	1.29	1.18E-12
Foxh1	14106	forkhead box H1	(1.382)	1.34	1.49E-07
Ttc30a1	78802	tetratricopeptide repeat domain 30A1	(1.384)	0.58	2.02E-04
Car9	230099	carbonic anhydrase 9	(1.389)	0.58	6.66E-04
Efcab2	68226	EF-hand calcium binding domain 2	(1.392)	0.68	4.40E-05
Rbbp9	26450	retinoblastoma binding protein 9	(1.392)	2.76	3.34E-15
Stard3	59045	START domain containing 3	(1.393)	0.57	7.57E-04
Ska1	66468	spindle and kinetochore associated complex subunit 1	(1.394)	10.46	2.29E-61
Tyw3	209584	tRNA-yW synthesizing protein 3 homolog (S. cerevisiae)	(1.396)	1.35	9.22E-13
Prdm14	383491	PR domain containing 14	(1.398)	0.97	1.19E-06
Zbtb5	230119	zinc finger and BTB domain containing 5	(1.400)	1.21	2.26E-17
Rbm48	269623	RNA binding motif protein 48	(1.402)	2.15	2.37E-12
Csad	246277	cysteine sulfinic acid decarboxylase	(1.402)	0.84	1.44E-06
Prr15l	217138	proline rich 15-like	(1.408)	0.59	1.06E-04
Dph2	67728	DPH2 homolog (S. cerevisiae)	(1.409)	0.87	1.89E-06
Cd79b	15985	CD79B antigen	(1.410)	1.89	1.45E-06
Aspscr1	68938	alveolar soft part sarcoma chromosome region, candidate 1 (human)	(1.413)	3.87	2.61E-27

Official Gene Symbol	Entrez Gene ID	Gene description	Normalized Fold Change LOG2(Cryo-2 / Fresh-1)	FPKM Mean of six samples	p-value of Cryo-2 vs Fresh-1
Slc15a4	100561	solute carrier family 15, member 4	(1.417)	2.03	9.31E-17
Fam135a	68187	family with sequence similarity 135, member A	(1.417)	2.16	1.89E-28
Capn10	23830	calpain 10	(1.418)	2.77	9.11E-21
Zfp143	20841	zinc finger protein 143	(1.418)	9.22	3.07E-65
Apela	100038489	apelin receptor early endogenous ligand	(1.421)	1.30	2.98E-04
Lmbr1l	74775	limb region 1 like	(1.422)	1.50	9.97E-12
Scx	20289	scleraxis	(1.423)	0.59	9.08E-05
Phf1	21652	PHD finger protein 1	(1.424)	0.74	1.32E-04
Snai1	20613	snail family zinc finger 1	(1.424)	1.14	3.49E-05
Trp53i13	216964	transformation related protein 53 inducible protein 13	(1.424)	0.57	2.03E-03
Ccdc28a	215814	coiled-coil domain containing 28A	(1.426)	1.25	3.55E-05
Wdr46	57315	WD repeat domain 46	(1.426)	10.04	3.41E-48
Rarb	218772	retinoic acid receptor, beta	(1.428)	1.62	4.49E-14
Cyth3	19159	cytohesin 3	(1.428)	1.48	2.95E-15
Tnk1	83813	tyrosine kinase, non-receptor, 1	(1.431)	0.93	8.67E-09
Hdhd2	76987	haloacid dehalogenase-like hydrolase domain containing 2	(1.431)	1.66	8.55E-14
Pald1	27355	phosphatase domain containing, paladin 1	(1.432)	0.61	1.61E-07
B9d1	27078	B9 protein domain 1	(1.437)	0.66	1.14E-02
Ddx28	71986	DEAD (Asp-Glu-Ala-Asp) box polypeptide 28	(1.438)	1.80	6.04E-09
AW146154	101835	expressed sequence AW146154	(1.439)	1.12	2.85E-10
Pex11a	18631	peroxisomal biogenesis factor 11 alpha	(1.443)	0.96	1.15E-08
Pwwp2b	101631	PWWP domain containing 2B	(1.444)	1.51	2.94E-11
S1pr1	13609	sphingosine-1-phosphate receptor 1	(1.450)	0.66	9.49E-06
Ccdc107	622404	coiled-coil domain containing 107	(1.451)	2.04	1.33E-05
Cphx1	105594	cytoplasmic polyadenylated homeobox 1	(1.451)	0.83	1.82E-05
Glt8d1	76485	glycosyltransferase 8 domain containing 1	(1.452)	0.79	3.65E-06
Hsf1	15499	heat shock factor 1	(1.453)	2.68	4.30E-16
Ccdc150	78016	coiled-coil domain containing 150	(1.455)	0.71	6.69E-08
Mvk	17855	mevalonate kinase	(1.457)	1.87	9.38E-09
Plcg2	234779	phospholipase C, gamma 2	(1.457)	1.40	1.48E-16
Zfp84	74352	zinc finger protein 84	(1.457)	1.38	2.11E-20
Eng	13805	endoglin	(1.459)	0.65	4.60E-07
Zbtb33	56805	zinc finger and BTB domain containing 33	(1.462)	0.73	7.80E-11
Senp7	66315	SUMO1/sentrin specific peptidase 7	(1.465)	0.78	1.45E-13
Pank2	74450	pantothenate kinase 2	(1.466)	0.54	1.94E-07
Eci3	69123	enoyl-Coenzyme A delta isomerase 3	(1.467)	0.59	4.77E-03
Thap11	59016	THAP domain containing 11	(1.469)	3.61	1.30E-17
Fam83g	69640	family with sequence similarity 83, member G	(1.476)	1.36	1.19E-15
Habp4	56541	hyaluronic acid binding protein 4	(1.477)	1.28	2.24E-09
Ptprn	19275	protein tyrosine phosphatase, receptor type, N	(1.478)	1.27	9.09E-15
Letmd1	68614	LETM1 domain containing 1	(1.481)	1.39	2.24E-13
Ap5s1	69596	adaptor-related protein 5 complex, sigma 1 subunit	(1.483)	1.28	3.25E-06
Nr2c1	22025	nuclear receptor subfamily 2, group C, member 1	(1.485)	0.74	3.58E-11
Atg4a_1	666468	autophagy related 4A, cysteine peptidase	(1.489)	2.22	2.44E-05
Acsbg1	94180	acyl-CoA synthetase bubblegum family member 1	(1.493)	1.13	1.04E-09
Atp6v0a2_1	21871	ATPase, H+ transporting, lysosomal V0 subunit A2	(1.498)	1.39	1.94E-04
Top1mt	72960	DNA topoisomerase 1, mitochondrial	(1.499)	1.67	3.47E-09
Sik1	17691	salt inducible kinase 1	(1.501)	1.44	1.28E-18
Tlcd1	68385	TLC domain containing 1	(1.501)	1.50	1.20E-14
Ankrd46	68839	ankyrin repeat domain 46	(1.505)	0.79	4.00E-11

Official Gene Symbol	Entrez Gene ID	Gene description	Normalized Fold Change LOG2(Cryo-2 / Fresh-1)	FPKM Mean of six samples	p-value of Cryo-2 vs Fresh-1
Spata5l1	214616	spermatogenesis associated 5-like 1	(1.507)	3.55	8.11E-14
Sox15	20670	SRY (sex determining region Y)-box 15	(1.513)	5.76	1.13E-17
Zfp939	233147	zinc finger protein 939	(1.514)	1.08	4.11E-16
Ap1s3	252903	adaptor-related protein complex AP-1, sigma 3	(1.514)	0.81	4.93E-07
Vps9d1	72325	VPS9 domain containing 1	(1.517)	0.81	5.37E-08
Tbc1d8b	245638	TBC1 domain family, member 8B	(1.520)	0.62	2.50E-11
E4f1	13560	E4F transcription factor 1	(1.521)	1.41	1.50E-12
Mov10l1	83456	Moloney leukemia virus 10-like 1	(1.522)	1.02	8.66E-12
Pdlim4	30794	PDZ and LIM domain 4	(1.524)	2.04	8.44E-07
Caskin2	140721	CASK-interacting protein 2	(1.527)	0.87	3.71E-13
Tpk1	29807	thiamine pyrophosphokinase	(1.530)	1.51	4.36E-04
Btk	12229	Bruton agammaglobulinemia tyrosine kinase	(1.530)	1.20	6.50E-11
Zfp62	22720	zinc finger protein 62	(1.534)	0.65	1.11E-10
Slc25a40	319653	solute carrier family 25, member 40	(1.536)	3.28	1.81E-27
Myrfl	237558	myelin regulatory factor-like	(1.536)	0.56	1.00E-05
Zfp335	329559	zinc finger protein 335	(1.536)	2.52	6.13E-36
Zfp426	235028	zinc finger protein 426	(1.537)	1.28	6.46E-25
Ccdc28b	66264	coiled coil domain containing 28B	(1.539)	1.06	3.82E-03
Cd59a	12509	CD59a antigen	(1.541)	2.71	1.44E-16
Odf2l	52184	outer dense fiber of sperm tails 2-like	(1.542)	2.19	1.35E-52
Manba	110173	mannosidase, beta A, lysosomal	(1.542)	0.56	3.79E-09
Gpatch3	242691	G patch domain containing 3	(1.546)	1.10	1.47E-06
Jam3	83964	junction adhesion molecule 3	(1.550)	0.51	2.14E-03
Pkn3	263803	protein kinase N3	(1.554)	0.61	1.26E-07
Ankrd26	232339	ankyrin repeat domain 26	(1.562)	1.10	2.62E-24
Dnajc5g	231098	DnaJ (Hsp40) homolog, subfamily C, member 5 gamma	(1.570)	0.84	1.13E-04
Thrsp	21835	thyroid hormone responsive	(1.574)	0.55	2.53E-02
Sumf2	67902	sulfatase modifying factor 2	(1.576)	2.83	5.35E-19
Mydgf	28106	DNA segment, Chr 17, Wayne State University 104, expressed	(1.577)	5.09	1.64E-13
Atg5	11793	autophagy related 5	(1.577)	9.27	4.63E-52
Aldh4a1	212647	aldehyde dehydrogenase 4 family, member A1	(1.586)	2.77	2.61E-29
Ntpcr	66566	nucleoside-triphosphatase, cancer-related	(1.593)	0.53	1.17E-05
Ptprj	19271	protein tyrosine phosphatase, receptor type, J	(1.594)	0.91	7.57E-23
Grhl1	195733	grainyhead-like 1 (Drosophila)	(1.594)	0.85	9.69E-10
Krt83	406219	keratin 83	(1.596)	0.55	5.12E-03
LOC102634499	102634499	-	(1.600)	2.62	8.37E-07
Ccnb1ip1	239083	cyclin B1 interacting protein 1	(1.605)	2.70	2.89E-14
LOC105247254	105247254	-	(1.605)	4.35	2.16E-33
Ankle1	234396	ankyrin repeat and LEM domain containing 1	(1.613)	0.56	2.60E-05
Abhd11os	69812	abhydrolase domain containing 11, opposite strand	(1.613)	0.77	3.60E-03
Zfp260	26466	zinc finger protein 260	(1.623)	3.09	6.12E-34
Prkcd	18753	protein kinase C, delta	(1.625)	0.84	4.01E-11
Cblc	80794	Casitas B-lineage lymphoma c	(1.632)	1.03	7.00E-06
Tysnd1	71767	trypsin domain containing 1	(1.637)	0.94	9.63E-08
Esrra	26379	estrogen related receptor, alpha	(1.637)	1.11	4.28E-09
Napb	17957	N-ethylmaleimide sensitive fusion protein attachment protein beta	(1.638)	0.80	1.55E-11
Nat9	66176	N-acetyltransferase 9 (GCN5-related, putative)	(1.645)	4.99	2.63E-19
Tubd1	56427	tubulin, delta 1	(1.646)	0.87	1.69E-06
Tbck	271981	TBC1 domain containing kinase	(1.649)	2.26	6.61E-66

Official Gene Symbol	Entrez Gene ID	Gene description	Normalized Fold Change LOG2(Cryo-2 / Fresh-1)	FPKM Mean of six samples	p-value of Cryo-2 vs Fresh-1
Ring1	19763	ring finger protein 1	(1.649)	0.64	3.38E-04
Sdhaf1	68332	succinate dehydrogenase complex assembly factor 1	(1.656)	2.30	2.01E-07
Zfp438	240186	zinc finger protein 438	(1.660)	0.85	1.50E-10
Anxa5	11747	annexin A5	(1.667)	1.63	1.16E-08
Gdf9	14566	growth differentiation factor 9	(1.668)	1.00	2.32E-07
Nxt2	237082	nuclear transport factor 2-like export factor 2	(1.672)	0.66	7.15E-07
Zfp941	407812	zinc finger protein 941	(1.683)	1.49	1.71E-16
Apobec3	80287	apolipoprotein B mRNA editing enzyme, catalytic polypeptide 3	(1.690)	1.21	1.56E-13
Rab39	270160	RAB39, member RAS oncogene family	(1.696)	1.03	1.02E-10
Slc12a4	20498	solute carrier family 12, member 4	(1.700)	1.43	5.22E-18
Sdr39u1	654795	short chain dehydrogenase/reductase family 39U, member 1	(1.701)	1.88	4.33E-11
Tsku	244152	tsukushi	(1.703)	1.16	2.20E-12
Pomt1	99011	protein-O-mannosyltransferase 1	(1.705)	1.31	9.35E-13
Plekho1	67220	pleckstrin homology domain containing, family O member 1	(1.712)	1.53	9.21E-08
Tmem81	74626	transmembrane protein 81	(1.721)	0.56	8.86E-04
Mettl15	76894	methyltransferase like 15	(1.726)	1.39	1.33E-10
BC033916	474160	cDNA sequence BC033916	(1.726)	0.59	1.38E-03
Poll	56626	polymerase (DNA directed), lambda	(1.727)	1.65	4.15E-14
Ina	226180	internexin neuronal intermediate filament protein, alpha	(1.729)	0.88	7.95E-07
Mtap	66902	methylthioadenosine phosphorylase	(1.746)	3.48	1.21E-31
Ybx1-ps2	94283	Y box protein 1, pseudogene 2	(1.749)	1.68	1.06E-10
Plekhd1	217682	pleckstrin homology domain containing, family D (with coiled-coil domains) member 1	(1.759)	0.54	9.45E-10
Rrp9	27966	RRP9, small subunit (SSU) processome component, homolog (yeast)	(1.769)	5.39	7.40E-29
Zfp772	232855	zinc finger protein 772	(1.770)	0.82	2.81E-08
Samhd1	56045	SAM domain and HD domain, 1	(1.775)	1.70	9.29E-31
Zfp101	22643	zinc finger protein 101	(1.778)	2.29	7.91E-25
Vgll1	170828	vestigial like 1 homolog (Drosophila)	(1.784)	1.54	8.70E-12
Zfp866	330788	zinc finger protein 866	(1.785)	1.55	1.73E-32
Tagap1	380608	T cell activation GTPase activating protein 1	(1.792)	4.16	1.83E-36
Slc35b3	108652	solute carrier family 35, member B3	(1.792)	0.78	9.17E-07
Malt1	240354	mucosa associated lymphoid tissue lymphoma translocation gene 1	(1.794)	0.87	5.33E-21
Trim14	74735	tripartite motif-containing 14	(1.794)	0.73	1.35E-08
Ocm	18261	oncomodulin	(1.797)	4.23	4.29E-20
Lyrm7	75530	LYR motif containing 7	(1.800)	1.14	2.81E-10
Bag2	213539	BCL2-associated athanogene 2	(1.800)	0.55	2.95E-04
Olfr836	258557	olfactory receptor 836	(1.802)	0.55	1.25E-02
Pcyt2	68671	phosphate cytidylyltransferase 2, ethanolamine	(1.802)	4.21	8.02E-34
Sqle	20775	squalene epoxidase	(1.807)	2.17	6.74E-23
Slc35a2	22232	solute carrier family 35 (UDP-galactose transporter), member A2	(1.809)	0.75	3.95E-09
Elmod3	232089	ELMO/CED-12 domain containing 3	(1.811)	1.25	8.50E-14
Sp140	434484	Sp140 nuclear body protein	(1.814)	2.20	1.44E-17
Asl	109900	argininosuccinate lyase	(1.832)	3.38	2.26E-20
Nprl3	17168	nitrogen permease regulator-like 3	(1.833)	0.85	1.32E-10

Official Gene Symbol	Entrez Gene ID	Gene description	Normalized Fold Change LOG2(Cryo-2 / Fresh-1)	FPKM Mean of six samples	p-value of Cryo-2 vs Fresh-1
Rsph3a	66832	radial spoke 3A homolog (Chlamydomonas)	(1.833)	1.07	3.42E-08
Ddr1	12305	discoidin domain receptor family, member 1	(1.833)	0.71	9.58E-16
Hoxb13	15408	homeobox B13	(1.836)	0.52	2.72E-03
Six1	20471	sine oculis-related homeobox 1	(1.846)	0.80	6.40E-09
Mybpc2	233199	myosin binding protein C, fast-type	(1.852)	0.74	2.85E-14
Ier5l	72500	immediate early response 5-like	(1.854)	0.95	5.69E-06
Gcc1	74375	golgi coiled coil 1	(1.860)	0.90	3.26E-14
Ccdc171	320226	coiled-coil domain containing 171	(1.860)	0.76	1.91E-19
Ap4m1	11781	adaptor-related protein complex AP-4, mu 1	(1.861)	2.36	1.31E-13
Anxa9	71790	annexin A9	(1.867)	2.29	4.45E-21
Rac3	170758	RAS-related C3 botulinum substrate 3	(1.871)	1.02	7.25E-05
Tcl1	21432	T cell lymphoma breakpoint 1	(1.886)	1.47	2.94E-08
Dolk	227697	dolichol kinase	(1.899)	1.02	1.62E-08
Mmab	77697	methylmalonic aciduria (cobalamin deficiency) type B homolog (human)	(1.901)	1.08	2.88E-13
Ccdc166	223648	coiled-coil domain containing 166	(1.901)	0.59	4.49E-06
Wnt6	22420	wingless-type MMTV integration site family, member 6	(1.917)	0.59	5.42E-06
LOC102632778	102632778	-	(1.920)	0.59	6.95E-04
Det1	76375	de-etiolated homolog 1 (Arabidopsis)	(1.937)	1.46	1.10E-15
Atg101	68118	autophagy related 101	(1.938)	3.88	2.12E-30
Hebp1	15199	heme binding protein 1	(1.945)	1.84	1.62E-10
Prom2	192212	prominin 2	(1.947)	0.61	5.43E-11
Cmbl	69574	carboxymethylenebutenolidase-like (Pseudomonas)	(1.952)	5.38	3.83E-46
Dnaaf1	68270	dynein, axonemal assembly factor 1	(1.956)	0.61	1.18E-07
Brat1	231841	BRCA1-associated ATM activator 1	(1.957)	0.93	1.71E-15
Hbegf	15200	heparin-binding EGF-like growth factor	(1.963)	0.62	1.88E-06
Hgh1	59053	HGH1 homolog	(1.966)	2.65	3.19E-22
Poc1b	382406	POC1 centriolar protein homolog B (Chlamydomonas)	(1.982)	1.75	1.04E-22
Spidr	224008	scaffolding protein involved in DNA repair	(2.004)	2.66	5.93E-33
Cycs-ps3	13066	cytochrome c, pseudogene 3	(2.011)	0.61	6.87E-03
Smim14	68552	small integral membrane protein 14	(2.029)	0.77	5.38E-18
Arhgef39	230098	Rho guanine nucleotide exchange factor (GEF) 39	(2.030)	1.45	1.09E-15
Sil1	81500	endoplasmic reticulum chaperone SIL1 homolog (S. cerevisiae)	(2.058)	1.28	2.74E-14
Eml3	225898	echinoderm microtubule associated protein like 3	(2.058)	0.90	6.30E-12
Dnajc27	217378	DnaJ (Hsp40) homolog, subfamily C, member 27	(2.059)	0.68	6.80E-17
Zgpat	229007	zinc finger, CCCH-type with G patch domain	(2.061)	1.61	7.73E-19
Fancf	100040608	Fanconi anemia, complementation group F	(2.075)	0.61	1.10E-04
Fbxo17	50760	F-box protein 17	(2.081)	0.54	2.14E-05
LOC105245334	105245334	-	(2.087)	1.89	6.44E-03
Npepl1	228961	aminopeptidase-like 1	(2.088)	0.58	4.90E-06
Flywch1	224613	FLYWCH-type zinc finger 1	(2.093)	0.51	5.65E-09
AW554918	225289	expressed sequence AW554918	(2.102)	0.58	2.62E-33
Cyb5d1	327951	cytochrome b5 domain containing 1	(2.104)	2.49	2.29E-39
Spata24	71242	spermatogenesis associated 24	(2.110)	0.70	6.49E-04
Thtpa	105663	thiamine triphosphatase	(2.127)	0.84	2.83E-11
Trmt2b	215201	TRM2 tRNA methyltransferase 2B	(2.130)	0.77	1.31E-13
Zfp12	231866	zinc finger protein 12	(2.140)	0.52	6.68E-13
Fbxo46	243867	F-box protein 46	(2.142)	0.74	1.35E-13
Ccar2	219158	cell cycle activator and apoptosis regulator 2	(2.147)	1.16	4.48E-24

Official Gene Symbol	Entrez Gene ID	Gene description	Normalized Fold Change LOG2(Cryo-2 / Fresh-1)	FPKM Mean of six samples	p-value of Cryo-2 vs Fresh-1
Cxx1a	66158	CAAX box 1A	(2.149)	0.73	2.05E-04
Igfbp7	29817	insulin-like growth factor binding protein 7	(2.151)	0.54	1.63E-03
Hal	15109	histidine ammonia lyase	(2.156)	0.65	6.00E-07
Mief2	237781	mitochondrial elongation factor 2	(2.158)	1.93	2.47E-23
Cptp	79554	ceramide-1-phosphate transfer protein	(2.174)	1.32	2.23E-17
Skiv2l	108077	superkiller viralicidic activity 2-like (S. cerevisiae)	(2.176)	0.97	1.34E-18
Dyrk1b	13549	dual-specificity tyrosine-(Y)-phosphorylation regulated kinase 1b	(2.185)	0.62	7.16E-11
Gramd4	223752	GRAM domain containing 4	(2.211)	0.92	4.27E-22
Prdm15	114604	PR domain containing 15	(2.212)	0.80	8.77E-28
Nqo1	18104	NAD(P)H dehydrogenase, quinone 1	(2.214)	1.08	2.08E-08
Grb14	50915	growth factor receptor bound protein 14	(2.217)	0.71	5.43E-08
Sntb2	20650	syntrophin, basic 2	(2.229)	2.10	2.39E-46
Zfp819	74400	zinc finger protein 819	(2.236)	0.55	4.57E-07
Sept1	54204	septin 1	(2.237)	0.76	1.16E-08
Zfp677	210503	zinc finger protein 677	(2.239)	0.83	3.74E-13
Nupr1	56312	nuclear protein transcription regulator 1	(2.243)	1.84	2.12E-06
Lpin3	64899	lipin 3	(2.246)	0.93	1.11E-16
Nsun4	72181	NOL1/NOP2/Sun domain family, member 4	(2.251)	0.92	9.87E-17
Rab38	72433	RAB38, member RAS oncogene family	(2.261)	2.04	1.20E-15
Pfkfb4	270198	6-phosphofructo-2-kinase/fructose-2,6-biphosphatase 4	(2.266)	0.67	4.30E-20
Rnd2	11858	Rho family GTPase 2	(2.268)	2.24	6.99E-08
D3Ertd751e	73852	DNA segment, Chr 3, ERATO Doi 751, expressed	(2.278)	0.52	2.92E-17
BC051142	407788	cDNA sequence BC051142	(2.286)	0.98	8.99E-14
Tmub1	64295	transmembrane and ubiquitin-like domain containing 1	(2.300)	0.62	2.33E-06
Cryl1	68631	crystallin, lambda 1	(2.306)	0.59	7.29E-05
LOC105244902	105244902	-	(2.323)	0.84	3.10E-04
Kng2	385643	kininogen 2	(2.340)	0.79	9.81E-11
Tubb3	22152	tubulin, beta 3 class III	(2.344)	1.08	3.42E-07
Zfp595	218314	zinc finger protein 595	(2.395)	0.82	2.40E-22
Cmss1	66497	cms small ribosomal subunit 1	(2.437)	2.49	1.90E-22
Tdpoz2	399673	TD and POZ domain containing 2	(2.449)	3.22	7.82E-21
Tdpoz1	207213	TD and POZ domain containing 1	(2.460)	0.60	7.05E-08
Aldh3b2	621603	aldehyde dehydrogenase 3 family, member B2	(2.494)	1.15	1.69E-14
Gemin8	237221	gem (nuclear organelle) associated protein 8	(2.496)	1.40	2.11E-16
P3h1	56401	prolyl 3-hydroxylase 1	(2.517)	0.80	6.82E-16
Slc27a4	26569	solute carrier family 27 (fatty acid transporter), member 4	(2.532)	1.58	7.43E-37
Arfgap3	66251	ADP-ribosylation factor GTPase activating protein 3	(2.535)	0.72	5.95E-03
Gdpd3	68616	glycerophosphodiester phosphodiesterase domain containing 3	(2.545)	0.51	1.35E-03
Prkra	23992	protein kinase, interferon inducible double stranded RNA dependent activator	(2.556)	0.57	2.07E-11
St3gal5	20454	ST3 beta-galactoside alpha-2,3-sialyltransferase 5	(2.565)	0.71	1.76E-10
Aspg	104816	asparaginase homolog (S. cerevisiae)	(2.580)	0.50	3.11E-12
Zfp82	330502	zinc finger protein 82	(2.581)	0.53	1.05E-10
Zscan10	332221	zinc finger and SCAN domain containing 10	(2.588)	0.64	8.63E-10
Qpctl	67369	glutaminyl-peptide cyclotransferase-like	(2.591)	0.55	3.08E-07
LOC105242877	105242877	-	(2.598)	0.55	1.39E-03
Fars2	69955	phenylalanine-tRNA synthetase 2 (mitochondrial)	(2.599)	1.40	4.64E-20

Official Gene Symbol	Entrez Gene ID	Gene description	Normalized Fold Change LOG2(Cryo-2 / Fresh-1)	FPKM Mean of six samples	p-value of Cryo-2 vs Fresh-1
Ticam2	225471	toll-like receptor adaptor molecule 2	(2.615)	0.51	2.49E-10
Ppp2r3d_2	19054	-	(2.625)	1.45	1.21E-04
Nup62cl	279706	nucleoporin 62 C-terminal like	(2.627)	0.71	2.90E-10
Morn2	378462	MORN repeat containing 2	(2.643)	0.62	4.63E-05
Hilpda	69573	hypoxia inducible lipid droplet associated	(2.644)	2.83	4.08E-19
BC017643	217370	cDNA sequence BC017643	(2.658)	1.03	3.94E-19
Thap7	69009	THAP domain containing 7	(2.659)	1.41	2.53E-19
Pigw	70325	phosphatidylinositol glycan anchor biosynthesis, class W	(2.665)	0.71	3.98E-12
Htr5b	15564	5-hydroxytryptamine (serotonin) receptor 5B	(2.684)	1.43	1.02E-17
Nhlrc3	212114	NHL repeat containing 3	(2.695)	0.79	1.21E-07
Pcbd1	13180	pterin 4 alpha carbinolamine dehydratase/dimerization cofactor of hepatocyte nuclear factor 1 alpha (TCF1) 1	(2.696)	1.16	6.75E-06
Itm2c	64294	integral membrane protein 2C	(2.696)	0.84	1.27E-09
Tmem52	69671	transmembrane protein 52	(2.701)	0.57	6.54E-04
Khdc1b	98582	KH domain containing 1B	(2.728)	0.53	1.24E-07
Zc3h10	103284	zinc finger CCCH type containing 10	(2.739)	1.20	6.11E-20
Eid3	66341	EP300 interacting inhibitor of differentiation 3	(2.752)	1.91	1.06E-15
Zfp324	243834	zinc finger protein 324	(2.753)	0.81	2.85E-25
Tmem82	213989	transmembrane protein 82	(2.763)	0.72	6.08E-10
Fundc2	67391	FUN14 domain containing 2	(2.960)	0.68	6.56E-14
Tex101	56746	testis expressed gene 101	(2.968)	0.83	2.60E-06
Tradd	71609	TNFRSF1A-associated via death domain	(2.981)	1.73	2.64E-16
Arntl2	272322	aryl hydrocarbon receptor nuclear translocator-like 2	(2.993)	1.53	4.46E-34
Ifi35	70110	interferon-induced protein 35	(3.025)	1.45	6.16E-14
LOC105246846	105246846	-	(3.057)	0.52	8.09E-07
Hspe1-rs1	628438	heat shock protein 1 (chaperonin 10), related sequence 1	(3.057)	0.74	2.72E-03
Tesc	57816	tescalcin	(3.075)	0.50	9.13E-04
Zp1	22786	zona pellucida glycoprotein 1	(3.094)	0.61	1.39E-08
Blcap	53619	bladder cancer associated protein homolog (human)	(3.122)	2.32	7.44E-44
Pla2g4f	271844	phospholipase A2, group IVF	(3.123)	0.73	9.72E-17
Cdc34-ps	111266	cell division cycle 34 homolog, pseudogene (S. cerevisiae)	(3.139)	0.60	1.39E-05
Tas2r108	57253	taste receptor, type 2, member 108	(3.165)	0.54	5.95E-04
Ddx11	320209	DEAD/H (Asp-Glu-Ala-Asp/His) box helicase 11	(3.196)	1.15	1.07E-36
Blnk	17060	B cell linker	(3.255)	1.19	2.01E-18
Lrrc48	74665	leucine rich repeat containing 48	(3.328)	0.60	4.42E-09
Phlda2	22113	pleckstrin homology-like domain, family A, member 2	(3.340)	0.79	2.74E-06
Pard6a	56513	par-6 family cell polarity regulator alpha	(3.393)	1.42	1.80E-14
Tmigd1	66601	transmembrane and immunoglobulin domain containing 1	(3.414)	0.61	6.64E-08
B3gnt2	53625	UDP-GlcNAc:betaGal beta-1,3-N-acetylglucosaminyltransferase 2	(3.430)	1.00	1.29E-20
Chit1	71884	chitinase 1 (chitotriosidase)	(3.431)	2.33	7.00E-31
Rec114	73673	REC114 meiotic recombination protein	(3.435)	0.55	1.79E-11
Snx21	101113	sorting nexin family member 21	(3.503)	0.65	1.14E-11
Otud6a	408193	OTU domain containing 6A	(3.515)	0.70	4.77E-05

Official Gene Symbol	Entrez Gene ID	Gene description	Normalized Fold Change LOG2(Cryo-2 / Fresh-1)	FPKM Mean of six samples	p-value of Cryo-2 vs Fresh-1
Csf2	12981	colony stimulating factor 2 (granulocyte-macrophage)	(3.543)	0.57	3.62E-06
Tuba3a	22144	tubulin, alpha 3A	(3.554)	0.97	5.06E-10
Tmsb15b2	100034363	thymosin beta 15b2	(3.610)	0.66	5.45E-04
Fgfr4	14186	fibroblast growth factor receptor 4	(3.615)	0.55	1.52E-13
Zfp791	244556	zinc finger protein 791	(3.627)	3.02	8.44E-52
Mthfs	107885	5, 10-methenyltetrahydrofolate synthetase	(3.672)	1.78	7.07E-11
Usp17le	625530	ubiquitin specific peptidase 17-like E	(3.691)	0.59	4.34E-03
D1Pas1	110957	DNA segment, Chr 1, Pasteur Institute 1	(3.696)	0.54	1.18E-13
Pla1a	85031	phospholipase A1 member A	(3.712)	0.51	1.93E-08
Slc2a5	56485	solute carrier family 2 (facilitated glucose transporter), member 5	(3.722)	0.52	7.47E-13
Ccnj	240665	cyclin J	(3.741)	0.96	1.29E-27
Fkbpl	56299	FK506 binding protein-like	(3.742)	0.61	1.62E-11
Sowahc	268301	sosondowah ankyrin repeat domain family member C	(3.752)	0.59	1.21E-20
Hyal1	15586	hyaluronoglucosaminidase 1	(3.776)	0.99	8.24E-17
Mpnd	68047	MPN domain containing	(3.779)	0.74	8.08E-11
Hormad1	67981	HORMA domain containing 1	(3.792)	0.64	1.83E-10
Guca2a	14915	guanylate cyclase activator 2a (guanylin)	(3.895)	0.54	4.58E-03
Gpr113	381628	G protein-coupled receptor 113	(3.902)	0.52	4.22E-16
Nudt17	78373	nudix (nucleoside diphosphate linked moiety X)-type motif 17	(3.947)	0.69	1.31E-09
Pts	19286	6-pyruvoyl-tetrahydropterin synthase	(3.957)	1.95	4.96E-16
Hpcal4	170638	hippocalcin-like 4	(3.979)	2.38	1.09E-84
LOC105245609	105245609	-	(4.026)	0.88	1.34E-03
H2-Ab1	14961	histocompatibility 2, class II antigen A, beta 1	(4.055)	1.39	4.48E-13
Calml4	75600	calmodulin-like 4	(4.189)	0.63	7.37E-08
Myd88	17874	myeloid differentiation primary response gene 88	(4.263)	0.55	1.05E-08
Hspa1b	15511	heat shock protein 1B	(4.298)	0.61	7.88E-13
Rbks	71336	ribokinase	(4.355)	0.58	2.39E-07
Zfp946	74149	zinc finger protein 946	(4.363)	0.91	1.01E-21
Xlr	22441	X-linked lymphocyte-regulated	(4.407)	0.57	7.98E-11
Tmem203	227615	transmembrane protein 203	(4.421)	0.51	2.85E-04
Scrn2	217140	secernin 2	(4.521)	0.52	4.85E-08
Pdk3	236900	pyruvate dehydrogenase kinase, isoenzyme 3	(4.655)	0.66	1.64E-10
LOC102637269	102637269	-	(4.772)	0.61	2.35E-03
Rps19-ps3	277692	ribosomal protein S19, pseudogene 3	(4.803)	2.10	2.24E-07
Zik1	22775	zinc finger protein interacting with K protein 1	(4.889)	0.53	5.02E-14
Trappc6a	67091	trafficking protein particle complex 6A	(5.029)	0.97	3.33E-08
Snx22	382083	sorting nexin 22	(5.169)	0.74	5.85E-14
Tssc1	380752	tumor suppressing subtransferable candidate 1	(5.180)	1.39	2.89E-21
LOC105243087	105243087	-	(5.201)	2.57	4.76E-06
Foxe1	110805	forkhead box E1	(5.211)	0.68	3.97E-06
Fbxo36	66153	F-box protein 36	(5.369)	1.01	1.54E-12
LOC105246843	105246843	-	(5.671)	0.53	1.14E-05
Hmgb1-ps2	100135427	high mobility group box 1, pseudogene 2	(5.773)	0.84	2.61E-05
Zfy1	22767	zinc finger protein 1, Y linked	(6.018)	0.51	1.14E-08
Acbd4	67131	acyl-Coenzyme A binding domain containing 4	(6.048)	0.65	1.74E-08
Twist1	22160	twist basic helix-loop-helix transcription factor 1	(6.055)	0.79	3.47E-08
Crygs	12970	crystallin, gamma S	(6.075)	0.83	5.14E-06
Sbp	20234	spermine binding protein	(19.745)	0.50	6.52E-06

Official Gene Symbol	Entrez Gene ID	Gene description	Normalized Fold Change LOG2(Cryo-2 / Fresh-1)	FPKM Mean of six samples	p-value of Cryo-2 vs Fresh-1
Rnase1	19752	ribonuclease, RNase A family, 1 (pancreatic)	(19.757)	0.51	1.46E-06
Ankrd39	109346	ankyrin repeat domain 39	(19.783)	0.52	4.89E-10
S100g	12309	S100 calcium binding protein G	(19.824)	0.53	5.07E-04
Hsd17b14	66065	hydroxysteroid (17-beta) dehydrogenase 14	(19.832)	0.54	4.02E-09
Zkscan6	52712	zinc finger with KRAB and SCAN domains 6	(19.890)	0.56	5.73E-11
Rhov	228543	ras homolog gene family, member V	(19.919)	0.57	2.03E-09
Rasl11a	68895	RAS-like, family 11, member A	(19.964)	0.59	9.29E-08
Krtap19-4	170654	keratin associated protein 19-4	(20.045)	0.62	3.32E-03
Pabpc1l2b-ps	668382	-	(20.110)	0.65	1.19E-07
Raet1d	56554	retinoic acid early transcript delta	(20.120)	0.65	7.10E-09
Ly6g	546644	-	(20.136)	0.66	2.00E-07
Bpifb1	228801	BPI fold containing family B, member 1	(20.171)	0.68	4.67E-10
Npb	208990	neuropeptide B	(20.206)	0.69	1.78E-05
LOC105245214	105245214	-	(20.210)	0.70	6.17E-05
Mrps36-ps1	100043826	mitichondrial ribosomal protein S36, pseudogene 1	(20.319)	0.75	2.06E-06
LOC105244402	105244402	-	(20.342)	0.76	1.22E-04
LOC105244781	105244781	-	(20.400)	0.79	9.69E-06
Nenf	66208	neuron derived neurotrophic factor	(20.446)	0.82	6.66E-07
Smim24	72273	small integral membrane protein 24	(20.456)	0.83	2.02E-06
Tmsb15l	399591	thymosin beta 15b like	(20.545)	0.88	4.20E-08
Chac2	68044	ChaC, cation transport regulator 2	(20.790)	1.04	6.86E-11
Krtdap	64661	keratinocyte differentiation associated protein	(20.793)	1.04	2.29E-06
Hist1h2aj	319174	histone cluster 1, H2aj	(20.834)	1.07	1.73E-05
Iah1	67732	isoamyl acetate-hydrolyzing esterase 1 homolog (S. cerevisiae)	(20.906)	1.13	4.24E-12
