# Supplementary material for: Transcriptomic Analysis of Vitrified–Warmed vs. Fresh Mouse Blastocysts: Cryo-Induced Physiological Mechanisms and Implantation Impact
Source: Int J Mol Sci. 2024 Aug 8;25(16):8658. doi: 10.3390/ijms25168658 (PMC11354596; doi:10.3390/ijms25168658)
Supplement: Supplementary file 1 [file ijms-25-08658-s001.zip › Supplementary Tables S2-S6.pdf]

Supplementary Table S2. Summary of most abundant Gene Ontology terms. Gene Ontology (GO) term analysis is uniquely enriched by differentially expressed genes (DEGs). Compared to fresh blastocysts, the upregulated DEGs in vitrified-warmed blastocysts were enriched for 32 GO terms, while the downregulated DEGs were enriched for 45 GO terms. BB: Biological Processes, CC: Cellular Components, and MF: Molecular Functions. The Up/Down-regulate DEGs column GO term is associated with upregulated or downregulated DEGs. Fold Enrichment indicates the ratio of the observed number of genes in the input gene set to the expected number in a random gene set. Enrichment FDR represents the statistical false discovery rate.

Gene Ontology Class	Up/Down Regulate DGEs	Fold Enrichment	GO Term Description	Enrichment FDR
BP	up	4.85	mitochondrial respiratory chain complex assembly	4.79E-07
BP	up	1.54	organonitrogen compound biosynthetic process	5.62E-04
BP	up	1.51	protein-containing complex assembly	3.89E-03
CC	up	1.72	mitochondrion	2.35E-10
CC	up	2.56	mitochondrial protein-containing complex	7.56E-06
CC	up	1.54	organelle subcompartment	3.01E-04
CC	up	3.34	mitochondrial ribosome	9.11E-04
CC	up	4.36	integral component of mitochondrial inner membrane	1.34E-03
CC	up	5.75	CENP-A containing chromatin	1.67E-03
BP	down	2.21	ncRNA metabolic process	8.91E-07
BP	down	1.84	cellular response to DNA damage stimulus	1.76E-05
BP	down	2.18	non-membrane-bounded organelle assembly	2.07E-04
BP	down	2.47	glycerolipid biosynthetic process	5.12E-04
BP	down	2.30	Golgi vesicle transport	5.12E-04
BP	down	1.53	protein modification by small protein conjugation or removal	6.22E-03
CC	down	1.62	mitochondrion	1.17E-08
CC	down	2.25	mitochondrial matrix	3.80E-05
CC	down	1.58	nucleolus	1.18E-03
CC	down	1.61	transferase complex	2.06E-03
CC	down	2.94	endoplasmic reticulum-Golgi intermediate compartment	2.22E-03
CC	down	1.53	endosome	3.93E-03
MF	down	2.39	catalytic activity acting on a nucleic acid	2.42E-10

MF	down	2.34	nuclease activity	1.97E-03
MF	down	1.52	RNA polymerase II cis-regulatory region sequence-specific DNA binding	1.58E-03

Note: This table includes the most significantly enriched GO terms. The filter criteria are Fold Enrichment greater than 1.5 and Enrichment FDR less than 0.01.

Supplementary Table S3. Summary of the Kyoto Encyclopedia of Genes and Genomes (KEGG) pathway enrichment results. Pathway enrichment of upregulated and downregulated genes was performed using the KEGG database. Fold Enrichment indicates the ratio of the observed number of genes in the input gene set to the expected number in a random gene set. Enrichment FDR represents the statistical false discovery rate.

Upregulated Pathway Name	Pathway ID	Enrichment FDR	Enrichment Fold	Altered Genes*
Thermogenesis	mmu04714	4.18E-03	2.38	12.28%
Chemical carcinogenesis-reactive oxygen species	mmu05208	4.18E-03	2.38	12.27%
Oxidative phosphorylation	mmu00190	1.33E-02	2.63	13.53%
Non-alcoholic fatty liver disease	mmu04932	6.38E-02	2.27	11.69%
Other types of O-glycan biosynthesis	mmu00514	7.51E-02	3.61	18.60%
MAPK signaling pathway	mmu04010	1.25E-01	1.78	9.18%
**Downregulated Pathway Name**	**Pathway ID**	**Enrichment FDR**	**Enrichment Fold**	**Altered Genes***
Herpes simplex virus 1 infection	mmu05168	3.84E-04	1.95	12.02%
NF-kappa B signaling pathway	mmu04064	2.37E-02	2.63	16.19%
Autophagy-animal	mmu04140	2.94E-02	2.32	14.29%
Aminoacyl-tRNA biosynthesis	mmu00970	7.92E-02	3.33	20.45%
Phosphatidylinositol signaling system	mmu04070	1.00E-01	2.37	14.58%
Fc epsilon RI signaling pathway	mmu04664	1.00E-01	2.71	16.67%
Glycosylphosphatidylinositol (GPI)-anchor	mmu00563	1.71E-01	3.75	23.08%

* % of genes in the pathway are present in the differential genes list (Vitrified-warmed vs. control blastocysts).

The filter criteria are Fold Enrichment greater than 1.5 and Enrichment FDR less than 0.2.

Supplementary Table S4. Primer list of reverse transcription polymerase chain reaction.

Gene symbol	Forward primer	Reverse primer	Accession number
GAPDH*	AACTTTGGCATTGTGGAAGG	ACACATTGGGGGTAGGAACA	GU214026
CDK6	TGTTTCAGCTTCTCCGAGGT	CTGGACTGGAGCAGGACTTC	BC120595
NFAT2	GGGTCAGTGTGACCGAAGAT	GGAAGTCAGAAGTGGGTGGA	NM_016791
DKK1	TTCTCTATGAGGGCGGGAAC	TGGTCAGAGGGCATGCATAT	AF030433
DKK3	ACCAGAGTGGACAGGTGGTC	TCACTGTCTCGGGTGCATAG	NM_015814
EGFR	CAGTGGGCAACCCTGAGTAT	GGGCCCTTAAATATGCCATT	NM_207655
MAPK10	TTGCCTTTTGTCAGGGATTC	CTGTCAAGGACAGCGTCGTA	NM_001081567

* GAPDH is used as a reference gene for RT-qPCR expression analysis.

Supplementary Table S5. The enrichment of 536 miRNAs with a *p*-value of 0.05 in vitrified-warmed blastocysts, as identified through GSEA v.4.1.0 analysis. The normalized enrichment score (NES) is a statistical score calculated based on the gene set, where a positive value indicates upregulation and a negative value indicates downregulation, although the magnitude does not correlate with miRNA expression levels. The NOM *p*-val. represents the normalized *p*-value.

Gene set	NES	NOM *p*-val.
MIR548AW	-1.1991	0.0365
MIR5582_5P	-1.2160	0.0408
MIR182_5P	-1.2166	0.0266
MIR6124	-1.2193	0.0408
MIR520A_3P	-1.2215	0.0475
TGGTGCT_MIR29A_MIR29B_MIR29C	-1.2216	0.0267
MIR4668_5P	-1.2258	0.0340
MIR4753_3P	-1.2272	0.0279
MIR5680	-1.2277	0.0364
MIR218_5P	-1.2308	0.0324
MIR4477A	-1.2312	0.0388
MIR3910	-1.2331	0.0365
MIR548AS_3P	-1.2346	0.0499
MIR6508_5P	-1.2372	0.0429
MIR3666	-1.2382	0.0392
MIR4719	-1.2390	0.0227
MIR4492	-1.2394	0.0325
MIR3714	-1.2394	0.0260
MIR23A_3P_MIR23B_3P	-1.2408	0.0112
MIR130B_3P	-1.2418	0.0209
TGCTTTG_MIR330	-1.2437	0.0415
MIR4269	-1.2444	0.0498
MIR454_3P	-1.2448	0.0260
MIR130A_5P	-1.2459	0.0357
MIR545_3P	-1.2467	0.0342
TGCCTTA_MIR124A	-1.2480	0.0133
MIR4295	-1.2484	0.0313
MIR4672	-1.2489	0.0303
MIR130A_3P	-1.2500	0.0253
MIR7856_5P	-1.2504	0.0281
MIR23C	-1.2510	0.0103
MIR135B_5P	-1.2511	0.0388
MIR4652_3P	-1.2515	0.0432
MIR301B_3P	-1.2515	0.0265
MIR5195_3P	-1.2515	0.0387

Gene set	NES	NOM *p*-val.
MIR301A_3P	-1.2521	0.0281
MIR32_3P	-1.2522	0.0347
MIR8067	-1.2528	0.0394
MIR410_3P	-1.2541	0.0351
AAGCCAT_MIR135A_MIR135B	-1.2544	0.0306
MIR6835_3P	-1.2563	0.0239
MIR135A_5P	-1.2602	0.0334
MIR3606_3P	-1.2605	0.0306
MIR5700	-1.2627	0.0383
MIR7110_3P	-1.2629	0.0235
MIR4496	-1.2637	0.0346
MIR494_3P	-1.2642	0.0200
MIR548P	-1.2644	0.0061
MIR519D_5P	-1.2646	0.0425
MIR16_2_3P	-1.2650	0.0351
MIR4251	-1.2662	0.0275
MIR543	-1.2665	0.0380
AAAGGGA_MIR204_MIR211	-1.2673	0.0391
MIR9_5P	-1.2684	0.0164
MIR5003_5P	-1.2686	0.0499
MIR302E	-1.2693	0.0165
TGCACTT_MIR519C_MIR519B_MIR519A	-1.2693	0.0113
MIR4422	-1.2704	0.0167
MIR485_3P	-1.2734	0.0477
CAGTATT_MIR200B_MIR200C_MIR429	-1.2735	0.0123
MIR548AA_MIR548AP_3P_MIR548T_3P	-1.2739	0.0051
MIR200C_3P	-1.2748	0.0092
MIR7154_5P	-1.2748	0.0286
MIR103A_3P_MIR107	-1.2749	0.0259
MIR145_5P	-1.2752	0.0258
MIR7157_5P	-1.2753	0.0379
TGAATGT_MIR181A_MIR181B_MIR181C_MIR181D	-1.2756	0.0165
MIR561_3P	-1.2768	0.0123
MIR6715B_5P	-1.2773	0.0394
MIR369_3P	-1.2795	0.0161
MIR200B_3P	-1.2806	0.0103
AAGCAAT_MIR137	-1.2807	0.0362
MIR510_3P	-1.2809	0.0210
MIR181D_5P	-1.2824	0.0092
MIR4328	-1.2831	0.0062
MIR4498	-1.2838	0.0403
MIR4796_3P	-1.2839	0.0329

Gene set	NES	NOM *p*-val.
MIR26B_5P	-1.2840	0.0123
MIR181C_5P	-1.2842	0.0020
CATTTCA_MIR203	-1.2847	0.0315
MIR499A_5P	-1.2849	0.0466
MIR539_3P	-1.2853	0.0474
MIR1200	-1.2855	0.0257
MIR646	-1.2856	0.0250
MIR181A_5P_MIR181B_5P	-1.2857	0.0052
MIR26A_5P	-1.2861	0.0125
MIR429	-1.2861	0.0093
MIR298	-1.2868	0.0418
MIR6739_5P	-1.2883	0.0235
MIR4311	-1.2890	0.0191
MIR6733_5P	-1.2902	0.0323
MIR2113	-1.2909	0.0260
MIR520E_5P	-1.2911	0.0478
MIR892C_3P	-1.2920	0.0353
MIR942_3P	-1.2926	0.0458
MIR148B_3P	-1.2941	0.0169
MIR1273H_5P	-1.2942	0.0389
MIR4275	-1.2945	0.0388
MIR452_5P	-1.2951	0.0424
MIR4447	-1.2959	0.0192
MIR148A_3P	-1.2967	0.0125
MIR1250_3P	-1.2980	0.0052
MIR548AQ_5P	-1.2984	0.0041
MIR4649_3P	-1.2988	0.0341
MIR656_3P	-1.2996	0.0114
MIR30D_3P	-1.3003	0.0063
MIR3160_5P	-1.3013	0.0346
MIR186_5P	-1.3036	0.0051
MIR518A_5P_MIR527	-1.3038	0.0185
MIR5189_5P	-1.3041	0.0433
MIR4738_3P	-1.3051	0.0380
MIR2054	-1.3062	0.0375
MIR152_3P	-1.3070	0.0150
MIR548L	-1.3073	0.0138
MIR1283	-1.3078	0.0031
MIR4676_3P	-1.3090	0.0285
MIR30E_3P	-1.3102	0.0073
MIR4760_3P	-1.3106	0.0163
MIR1303	-1.3123	0.0342

Gene set	NES	NOM *p*-val.
MIR4465	-1.3126	0.0075
GTCTTCC_MIR7	-1.3131	0.0388
MIR1285_3P	-1.3134	0.0381
MIR539_5P	-1.3137	0.0085
MIR6884_5P	-1.3144	0.0452
MIR4802_3P	-1.3150	0.0287
TCATCTC_MIR143	-1.3155	0.0368
MIR4291	-1.3157	0.0107
MIR499B_3P	-1.3158	0.0379
MIR548Y	-1.3161	0.0031
MIR7113_5P	-1.3170	0.0498
MIR6885_3P	-1.3170	0.0063
MIR3674	-1.3174	0.0379
MIR6073	-1.3180	0.0402
MIR605_3P	-1.3191	0.0240
MIR181B_2_3P_MIR181B_3P	-1.3193	0.0209
MIR548A_5P	-1.3209	0.0062
MIR4801	-1.3213	0.0264
MIR7844_5P	-1.3214	0.0380
MIR548O_5P_MIR548W	-1.3216	0.0031
MIR633	-1.3219	0.0448
MIR642A_3P_MIR642B_3P	-1.3221	0.0376
MIR29B_2_5P	-1.3229	0.0144
MIR548I	-1.3232	0.0010
MIR513A_3P_MIR513C_3P	-1.3232	0.0021
MIR8061	-1.3234	0.0292
MIR3140_3P	-1.3236	0.0226
MIR4478	-1.3236	0.0309
MIR6844	-1.3237	0.0076
MIR103A_1_5P	-1.3239	0.0436
MIR548AP_5P_MIR548J_5P	-1.3249	0.0031
MIR548AS_5P	-1.3256	0.0000
MIR3686	-1.3257	0.0107
MIR4429	-1.3258	0.0107
MIR208A_5P	-1.3259	0.0243
MIR208B_5P	-1.3262	0.0186
MIR4742_3P	-1.3262	0.0166
MIR499A_3P	-1.3268	0.0386
MIR190B_3P	-1.3271	0.0437
MIR106A_3P	-1.3273	0.0432
MIR103A_2_5P	-1.3276	0.0307
MIR509_3_5P	-1.3285	0.0359

Gene set	NES	NOM *p*-val.
MIR4261	-1.3288	0.0052
AACTGGA_MIR145	-1.3290	0.0187
MIR548AB	-1.3293	0.0021
MIR548AD_5P_MIR548AE_5P_MIR548AY_5P_MIR548B_5P_MIR548D_5P	-1.3295	0.0031
MIR30A_3P	-1.3298	0.0042
MIR548BC	-1.3300	0.0020
MIR4255	-1.3305	0.0230
MIR548AK_MIR548AM_5P_MIR548C_5P_MIR548H_5P_MIR548O_5P_MIR548AU_5P	-1.3308	0.0051
MIR5681A	-1.3310	0.0387
MIR125B_2_3P	-1.3321	0.0420
MIR3692_3P	-1.3329	0.0109
MIR514B_5P	-1.3334	0.0235
MIR1252_3P	-1.3334	0.0096
MIR421	-1.3335	0.0344
MIR526B_5P	-1.3336	0.0372
TAATAAT_MIR126	-1.3347	0.0135
MIR548BB_5P	-1.3357	0.0000
MIR3688_3P	-1.3357	0.0104
MIR4699_3P	-1.3360	0.0031
AACATTC_MIR4093P	-1.3362	0.0380
MIR548E_3P	-1.3363	0.0021
MIR548A_3P	-1.3364	0.0020
MIR548AR_5P	-1.3367	0.0010
MIR509_5P	-1.3367	0.0245
MIR323A_3P	-1.3368	0.0281
MIR7161_5P	-1.3369	0.0321
MIR12131	-1.3371	0.0289
MIR320A_3P_MIR320B_MIR320C_MIR320D	-1.3371	0.0098
MIR196A_1_3P	-1.3375	0.0094
MIR3190_5P	-1.3387	0.0403
MIR1253	-1.3399	0.0293
CAGTGTT_MIR141_MIR200A	-1.3400	0.0053
MIR548AN	-1.3409	0.0010
MIR3617_3P	-1.3412	0.0473
MIR5585_5P	-1.3416	0.0359
MIR4272	-1.3419	0.0192
MIR548F_3P	-1.3420	0.0031
MIR6778_3P	-1.3423	0.0230
MIR22_5P	-1.3424	0.0250
MIR487A_3P	-1.3428	0.0375
ATACTGT_MIR144	-1.3441	0.0216
MIR329_5P	-1.3444	0.0432

Gene set	NES	NOM *p*-val.
MIR3124_3P	-1.3447	0.0181
MIR4531	-1.3450	0.0106
MIR3941	-1.3461	0.0118
MIR517_5P	-1.3462	0.0419
MIR6760_5P	-1.3463	0.0417
MIR6088	-1.3467	0.0168
MIR4659B_5P	-1.3479	0.0449
MIR12123	-1.3481	0.0000
CTCCAAG_MIR432	-1.3483	0.0468
MIR10527_5P	-1.3489	0.0020
MIR183_3P	-1.3490	0.0305
MIR3187_5P	-1.3490	0.0387
MIR4680_3P	-1.3494	0.0130
MIR4530	-1.3499	0.0284
MIR3689A_5P_MIR3689B_5P_MIR3689E_MIR3689F	-1.3499	0.0443
MIR4696	-1.3504	0.0414
MIR216B_5P	-1.3504	0.0410
MIR3153	-1.3509	0.0153
MIR10393_5P	-1.3511	0.0369
MIR4643	-1.3512	0.0145
MIR4699_5P	-1.3513	0.0305
MIR548AZ_3P	-1.3515	0.0010
MIR498_3P	-1.3516	0.0164
MIR506_5P	-1.3537	0.0364
MIR6515_3P	-1.3554	0.0154
MIR6732_3P	-1.3563	0.0469
MIR6730_5P	-1.3563	0.0316
MIR7152_3P	-1.3564	0.0406
MIR6072	-1.3565	0.0458
MIR125A_5P	-1.3567	0.0064
MIR4647_MIR4662B	-1.3576	0.0441
MIR7853_5P	-1.3587	0.0110
MIR186_3P	-1.3587	0.0163
MIR12120	-1.3590	0.0204
TTTTGAG_MIR373	-1.3593	0.0111
MIR105_5P	-1.3599	0.0123
MIR155_3P	-1.3601	0.0393
MIR125B_5P	-1.3605	0.0043
MIR485_5P	-1.3607	0.0239
GTGCCAT_MIR183	-1.3609	0.0252
MIR4662A_5P	-1.3615	0.0345
MIR5583_5P	-1.3616	0.0367

Gene set	NES	NOM *p*-val.
MIR548F_5P	-1.3621	0.0000
MIR4797_3P	-1.3622	0.0470
MIR3192_3P	-1.3623	0.0431
MIR4319	-1.3626	0.0031
MIR664A_3P	-1.3629	0.0052
MIR361_5P	-1.3632	0.0196
MIR2115_3P	-1.3634	0.0100
MIR3140_5P	-1.3634	0.0356
MIR6860	-1.3642	0.0378
MIR4789_3P	-1.3648	0.0032
MIR4717_5P	-1.3654	0.0430
MIR3158_5P	-1.3656	0.0210
MIR513B_5P	-1.3656	0.0096
MIR4735_3P	-1.3667	0.0208
GAGACTG_MIR452	-1.3676	0.0300
MIR93_3P	-1.3678	0.0202
MIR3152_5P	-1.3688	0.0462
MIR558	-1.3690	0.0242
MIR3619_5P	-1.3691	0.0066
MIR466	-1.3694	0.0021
MIR1270	-1.3694	0.0436
MIR4677_5P	-1.3703	0.0065
MIR548AR_3P	-1.3706	0.0000
MIR371A_5P	-1.3706	0.0151
MIR100_3P	-1.3710	0.0431
MIR154_3P	-1.3716	0.0462
MIR892C_5P	-1.3719	0.0086
MIR5580_3P	-1.3728	0.0022
MIR1226_3P	-1.3728	0.0361
MIR9500	-1.3730	0.0092
MIR559	-1.3736	0.0010
MIR20B_3P	-1.3736	0.0349
MIR7151_3P	-1.3737	0.0178
MIR630	-1.3737	0.0354
MIR6074	-1.3738	0.0089
MIR548AJ_5P_MIR548G_5P_MIR548X_5P	-1.3742	0.0021
MIR4477B	-1.3744	0.0065
MIR3182	-1.3758	0.0098
ATGCAGT_MIR217	-1.3760	0.0275
MIR577	-1.3762	0.0022
MIR7973	-1.3765	0.0481
MIR548M	-1.3785	0.0139

Gene set	NES	NOM *p*-val.
MIR16_5P	-1.3785	0.0000
MIR3942_3P	-1.3790	0.0021
MIR4524A_3P	-1.3790	0.0055
MIR3935	-1.3795	0.0453
MIR5000_5P	-1.3796	0.0281
MIR148A_5P	-1.3817	0.0273
MIR6776_5P	-1.3817	0.0410
MIR606	-1.3818	0.0349
MIR21_3P	-1.3820	0.0214
MIR761	-1.3825	0.0067
MIR3150B_3P	-1.3826	0.0209
MIR4511	-1.3829	0.0186
MIR4799_5P	-1.3834	0.0000
ATACCTC_MIR202	-1.3846	0.0078
MIR8065	-1.3847	0.0107
MIR15B_5P	-1.3847	0.0010
ATTCTTT_MIR186	-1.3851	0.0044
MIR4709_3P	-1.3852	0.0128
MIR552_5P	-1.3853	0.0081
MIR6838_5P	-1.3857	0.0000
TTTGCAG_MIR518A2	-1.3863	0.0056
MIR5089_5P	-1.3864	0.0239
MIR7114_5P	-1.3867	0.0106
MIR214_3P	-1.3867	0.0089
MIR664B_3P	-1.3879	0.0010
MIR122B_3P	-1.3889	0.0224
MIR4653_3P	-1.3890	0.0461
MIR621	-1.3891	0.0496
MIR513B_3P	-1.3891	0.0154
GTTTGTT_MIR495	-1.3892	0.0044
MIR4694_3P	-1.3899	0.0032
MIR4524A_5P	-1.3900	0.0033
MIR2355_5P	-1.3902	0.0196
MIR4673	-1.3905	0.0197
MIR513C_5P	-1.3913	0.0162
MIR331_5P	-1.3917	0.0453
MIR302C_5P	-1.3919	0.0000
MIR6737_5P	-1.3933	0.0495
MIR202_5P	-1.3937	0.0230
MIR544B	-1.3938	0.0034
MIR205_3P	-1.3941	0.0000
AATGTGA_MIR23A_MIR23B	-1.3948	0.0010

Gene set	NES	NOM *p*-val.
MIR4683	-1.3954	0.0479
MIR6859_5P	-1.3959	0.0067
MIR653_3P	-1.3961	0.0154
MIR195_5P	-1.3963	0.0000
MIR4784	-1.3965	0.0203
MIR579_3P	-1.3966	0.0000
MIR4418	-1.3967	0.0168
MIR4420	-1.3970	0.0101
MIR6757_3P	-1.3975	0.0180
MIR500A_5P	-1.3981	0.0093
MIR10399_5P	-1.3982	0.0011
MIR142_5P	-1.3982	0.0011
MIR4701_3P	-1.3985	0.0486
MIR10395_3P	-1.3986	0.0153
MIR192_3P	-1.3994	0.0217
MIR3171	-1.3994	0.0322
MIR338_5P	-1.4000	0.0000
MIR8087	-1.4007	0.0179
MIR613	-1.4009	0.0011
CTCAAGA_MIR526B	-1.4009	0.0390
MIR3120_5P	-1.4021	0.0374
MIR4324	-1.4022	0.0033
MIR548AV_3P	-1.4026	0.0090
MIR612	-1.4030	0.0229
MIR3925_5P	-1.4031	0.0094
MIR4762_3P	-1.4032	0.0066
MIR3920	-1.4033	0.0157
MIR12125	-1.4034	0.0293
MIR3916	-1.4052	0.0044
MIR497_5P	-1.4060	0.0000
MIR1236_3P	-1.4075	0.0162
MIR31_3P	-1.4091	0.0390
MIR555	-1.4091	0.0474
MIR133A_3P_MIR133B	-1.4098	0.0033
MIR922	-1.4102	0.0056
MIR3074_3P	-1.4112	0.0203
MIR6513_3P	-1.4114	0.0153
MIR4276	-1.4114	0.0044
MIR424_5P	-1.4116	0.0000
MIR5589_5P	-1.4119	0.0332
MIR3125	-1.4123	0.0033
MIR5590_3P	-1.4128	0.0011

Gene set	NES	NOM *p*-val.
MIR6736_5P	-1.4129	0.0425
MIR7159_5P	-1.4132	0.0000
MIR3133	-1.4136	0.0000
MIR4670_3P	-1.4145	0.0120
MIR4789_5P	-1.4146	0.0000
MIR6747_3P	-1.4147	0.0157
MIR34A_3P	-1.4148	0.0268
MIR6833_5P	-1.4154	0.0108
MIR6504_3P	-1.4166	0.0000
MIR1262	-1.4172	0.0441
MIR3912_5P	-1.4177	0.0165
MIR6505_3P	-1.4183	0.0218
MIR6773_5P	-1.4183	0.0331
ACCAAAG_MIR9	-1.4184	0.0000
MIR215_5P	-1.4186	0.0401
MIR3130_5P	-1.4187	0.0356
MIR3913_3P	-1.4192	0.0227
MIR1912_3P	-1.4198	0.0259
MIR4540	-1.4199	0.0387
MIR4524B_5P	-1.4208	0.0033
MIR382_3P	-1.4215	0.0239
MIR5003_3P	-1.4228	0.0011
MIR1252_5P	-1.4229	0.0011
MIR548AO_5P_MIR548AX	-1.4235	0.0081
MIR4733_5P	-1.4236	0.0142
MIR580_5P	-1.4241	0.0046
MIR551B_5P	-1.4245	0.0000
MIR4504	-1.4248	0.0059
MIR4794	-1.4249	0.0098
MIR2114_5P	-1.4251	0.0398
MIR6828_5P	-1.4261	0.0092
MIR3143	-1.4261	0.0010
CTCAGGG_MIR125B_MIR125A	-1.4265	0.0032
MIR10523_5P	-1.4268	0.0011
MIR4782_5P	-1.4269	0.0104
MIR206	-1.4270	0.0021
MIR4426	-1.4276	0.0092
MIR3973	-1.4289	0.0022
MIR3529_3P	-1.4291	0.0000
MIR141_5P	-1.4293	0.0282
MIR4451	-1.4304	0.0118
MIR16_1_3P	-1.4307	0.0045

Gene set	NES	NOM *p*-val.
MIR12135	-1.4311	0.0033
MIR664A_5P	-1.4311	0.0230
MIR6819_5P	-1.4344	0.0248
MIR8080	-1.4349	0.0107
MIR4307	-1.4354	0.0000
MIR4778_5P	-1.4362	0.0057
MIR587	-1.4363	0.0000
MIR1306_5P	-1.4366	0.0310
MIR4309	-1.4366	0.0322
TCTGATA_MIR361	-1.4379	0.0147
MIR4773	-1.4391	0.0056
MIR4762_5P	-1.4392	0.0130
MIR3123	-1.4401	0.0000
MIR380_3P	-1.4409	0.0033
MIR12124	-1.4415	0.0000
MIR6760_3P	-1.4415	0.0147
MIR4639_3P	-1.4416	0.0246
MIR3977	-1.4420	0.0070
MIR1184	-1.4421	0.0023
MIR5706	-1.4436	0.0035
MIR3115	-1.4453	0.0370
MIR26A_1_3P	-1.4467	0.0156
MIR4684_5P	-1.4471	0.0035
MIR1_3P	-1.4480	0.0010
MIR4703_3P	-1.4483	0.0301
MIR5584_5P	-1.4519	0.0000
MIR136_5P	-1.4528	0.0122
MIR372_5P	-1.4531	0.0045
MIR7153_3P	-1.4531	0.0186
MIR499B_5P	-1.4533	0.0150
ACACTAC_MIR1423P	-1.4535	0.0071
MIR3678_3P	-1.4542	0.0098
MIR4768_3P	-1.4547	0.0034
MIR3685	-1.4561	0.0034
MIR4289	-1.4582	0.0183
MIR4666A_3P	-1.4583	0.0000
MIR520G_5P	-1.4598	0.0219
MIR655_3P	-1.4599	0.0010
MIR5699_5P	-1.4611	0.0084
MIR384	-1.4613	0.0061
MIR6770_5P	-1.4614	0.0011
MIR1908_5P	-1.4621	0.0413

Gene set	NES	NOM *p*-val.
MIR5009_3P	-1.4623	0.0023
ATGAAGG_MIR205	-1.4633	0.0023
MIR4482_3P	-1.4650	0.0000
MIR3198	-1.4653	0.0194
MIR26A_2_3P	-1.4659	0.0172
MIR216A_5P	-1.4661	0.0048
MIR4742_5P	-1.4664	0.0023
MIR365B_5P	-1.4666	0.0337
MIR374C_5P	-1.4672	0.0000
MIR3650	-1.4680	0.0226
MIR4494	-1.4686	0.0251
MIR4424	-1.4714	0.0035
MIR302A_5P	-1.4736	0.0023
CATGTAA_MIR496	-1.4736	0.0023
MIR1243	-1.4736	0.0174
MIR3663_5P	-1.4742	0.0218
MIR652_5P	-1.4753	0.0187
MIR616_3P	-1.4763	0.0179
MIR567	-1.4767	0.0036
MIR2467_5P	-1.4768	0.0147
MIR885_5P	-1.4795	0.0073
MIR4666A_5P	-1.4809	0.0033
TTCCGTT_MIR191	-1.4814	0.0294
MIR511_3P	-1.4848	0.0057
MIR365A_5P	-1.4872	0.0339
MIR4495	-1.4872	0.0010
MIR6772_3P	-1.4905	0.0143
MIR548AT_5P	-1.4922	0.0010
MIR4659A_5P	-1.4945	0.0103
MIR582_5P	-1.4951	0.0000
MIR4432	-1.4955	0.0132
MIR6820_3P	-1.4970	0.0087
MIR8057	-1.5000	0.0049
MIR300	-1.5019	0.0000
MIR6832_5P	-1.5019	0.0090
MIR3065_5P	-1.5026	0.0000
MIR888_5P	-1.5034	0.0000
MIR3670	-1.5038	0.0108
MIR4490	-1.5040	0.0036
MIR508_5P	-1.5041	0.0024
MIR224_5P	-1.5070	0.0012
MIR1304_3P	-1.5074	0.0098

Gene set	NES	NOM *p*-val.
MIR6781_3P	-1.5121	0.0066
MIR8063	-1.5124	0.0000
MIR1296_3P	-1.5158	0.0243
ATGTTAA_MIR302C	-1.5168	0.0000
MIR381_3P	-1.5174	0.0000
MIR5588_3P	-1.5181	0.0081
MIR6808_3P	-1.5213	0.0113
MIR33A_3P	-1.5256	0.0000
MIR5187_3P	-1.5257	0.0050
MIR4436B_5P	-1.5262	0.0024
MIR3164	-1.5326	0.0075
MIR150_5P	-1.5436	0.0011
MIR1193	-1.5469	0.0141
MIR519A_2_5P_MIR520B_5P	-1.5479	0.0000
MIR12127	-1.5511	0.0052
MIR4480	-1.5525	0.0081
MIR497_3P	-1.5549	0.0000
MIR504_3P	-1.5549	0.0023
MIR935	-1.5550	0.0024
MIR4536_5P	-1.5551	0.0153
MIR3929	-1.5634	0.0025
MIR1257	-1.5651	0.0038
MIR4263	-1.5656	0.0000
MIR1285_5P	-1.5709	0.0066
MIR10524_5P	-1.5801	0.0025
MIR6771_3P	-1.5803	0.0049
MIR6858_5P	-1.5865	0.0026
MIR548O_3P	-1.5897	0.0000
ACATTCC_MIR1_MIR206	-1.5925	0.0000
CCAGGTT_MIR490	-1.6144	0.0025
MIR1323	-1.6172	0.0000
MIR5100	-1.6242	0.0012
MIR4637	-1.6278	0.0013
MIR4274	-1.6502	0.0053
MIR496	-1.6535	0.0025
MIR556_3P	-1.6597	0.0000
MIR335_5P	-1.6890	0.0000
MIR197_3P	-1.7138	0.0000
MIR4689	-1.7749	0.0000

Supplementary Table S6. Supplementary Table 6. The results of the miRCURY experimental analysis identified miRNAs with differential expression between cryo-embryos and fresh embryos. With a fold-change cutoff of 1.5 and a quality threshold exceeding level "B," the analysis detected 97 out of 768 tested miRNAs as differentially expressed. Of these, 14 miRNAs were confirmed as potential candidates by intersecting the experimental results with the predicted results from gene set enrichment analysis (GSEA). The fold change was calculated as the ratio of miRNA expression in cryo-embryos to fresh embryos. The GSEA column indicates whether the miRNA appeared in the list of miRNAs predicted by GSEA, and the p-value represents the statistical significance of the GSEA prediction. The microRNA intensity quality categorizes the miRNA expression levels as follows:

S: CT values were <30, indicating relatively high microRNA expression in both fresh and vitrified-warmed blastocysts.

A: MicroRNA expression was relatively high in both samples, with CT values <30 in one sample and >30 in the other.

B: MicroRNA expression was relatively low in fresh and vitrified-warmed blastocysts, with CT values >30 in both samples.

C: The data exceeded the threshold cycle cutoff in one or both samples.

**microRNA**	**Fold Change**	**GSEA**	***p*-value of GSEA**	**microRNA intensity quality**
miR-503-5p	3.89	NF	--	B
miR-878-3p	3.31	NF	--	B
miR-881-3p	3.19	NF	--	B
miR-297a-3p	2.95	NF	--	B
miR-147b	2.93	NF	--	B
miR-186-5p	2.85	Intersection	0.005	B
miR-96-5p	2.42	NF	--	B
miR-669f-3p	1.98	NF	--	B
miR-466f	1.89	NF	--	B
miR-350	1.87	NF	--	B
miR-324-5p	1.78	NF	--	B
miR-423-5p	1.76	NF	--	B
miR-324-3p	1.76	NF	--	B
miR-335-3p	1.75	NF	--	B
miR-151a-5p	1.69	NF	--	B
miR-466d-3p	1.65	NF	--	B
miR-382-5p	1.64	NF	--	B
miR-15b-5p	1.6	Intersection	0.001	A
miR-742-3p	1.59	NF	--	B
miR-298-5p	1.55	NF	--	B
miR-296-5p	1.55	NF	--	B
miR-34a-5p	1.55	NF	--	B

microRNA	Fold Change	GSEA	*p*-value of GSEA	microRNA intensity quality
miR-466c-5p	1.53	NF	--	B
miR-105	0.67	NF	--	B
miR-200b-3p	0.66	Intersection	0.010	S
miR-484	0.66	NF	--	B
miR-191-5p	0.66	NF	--	S
miR-192-5p	0.65	NF	--	B
miR-320a	0.65	NF	--	S
miR-361-5p	0.65	Intersection	0.020	B
miR-93-3p	0.65	Intersection	0.020	B
miR-328-3p	0.64	NF	--	B
miR-15b-3p	0.62	NF	--	B
miR-340-5p	0.61	NF	--	B
miR-331-3p	0.61	NF	--	B
miR-28-5p	0.61	NF	--	B
miR-669l-5p	0.61	NF	--	B
miR-1895	0.61	NF	--	B
miR-203a-3p	0.6	NF	--	B
miR-672-5p	0.6	NF	--	B
miR-669a-5p	0.6	NF	--	S
miR-297c-5p	0.59	NF	--	B
miR-9-5p	0.59	Intersection	0.016	B
miR-92a-3p	0.59	NF	--	S
miR-106b-3p	0.59	NF	--	B
miR-126-3p	0.58	NF	--	B
miR-2137	0.58	NF	--	S
miR-30e-3p	0.58	Intersection	0.007	B
miR-214-3p	0.57	Intersection	0.009	B
miR-339-5p	0.56	NF	--	B
miR-669c-5p	0.56	NF	--	B
miR-148b-3p	0.55	Intersection	0.017	B
miR-127-3p	0.55	NF	--	B
miR-465c-5p	0.55	NF	--	B
miR-712-5p	0.55	NF	--	S
miR-409a-3p	0.55	NF	--	B
miR-210-3p	0.54	NF	--	B
miR-379-5p	0.54	NF	--	B
let-7a-5p	0.52	NF	--	B
miR-301b-3p	0.5	Intersection	0.027	B
miR-433-3p	0.5	NF	--	B
miR-744-5p	0.49	NF	--	B

microRNA	Fold Change	GSEA	*p*-value of GSEA	microRNA intensity quality
miR-674-3p	0.49	NF	--	B
miR-208a-3p	0.48	NF	--	B
miR-340-3p	0.47	NF	--	B
miR-706	0.46	NF	--	S
miR-669o-5p	0.46	NF	--	B
miR-466h-5p	0.45	NF	--	B
miR-133b	0.44	NF	--	B
miR-103a-2-5p	0.44	Intersection	0.031	B
miR-1983	0.43	NF	--	B
miR-501-5p	0.43	NF	--	B
miR-134-5p	0.42	NF	--	B
miR-743b-5p	0.42	NF	--	B
miR-30d-3p	0.42	Intersection	0.006	B
miR-667-3p	0.42	NF	--	B
miR-130b-3p	0.41	Intersection	0.021	B
miR-139-3p	0.41	NF	--	B
miR-150-5p	0.39	Intersection	0.001	B
miR-133a-3p	0.38	NF	--	B
miR-741-3p	0.38	NF	--	B
miR-652-3p	0.38	NF	--	B
miR-467f	0.38	NF	--	B
miR-142-3p	0.37	NF	--	B
miR-541-5p	0.36	NF	--	B
miR-330-3p	0.36	NF	--	B
miR-1949	0.36	NF	--	S
miR-669n	0.33	NF	--	B
miR-200b-5p	0.33	NF	--	B
miR-195-3p	0.3	NF	--	B
miR-1839-3p	0.3	NF	--	B
miR-1198-5p	0.28	NF	--	B
miR-365a-3p	0.26	NF	--	B
let-7b-5p	0.24	NF	--	B
miR-486-5p	0.09	NF	--	B
miR-451a	0.06	NF	--	A
miR-144-3p	0.06	NF	--	B
