# Supplementary figures and images for: Transcriptomic Analysis of Vitrified–Warmed vs. Fresh Mouse Blastocysts: Cryo-Induced Physiological Mechanisms and Implantation Impact
Source: Int J Mol Sci. 2024 Aug 8;25(16):8658. doi: 10.3390/ijms25168658 (PMC11354596; doi:10.3390/ijms25168658)

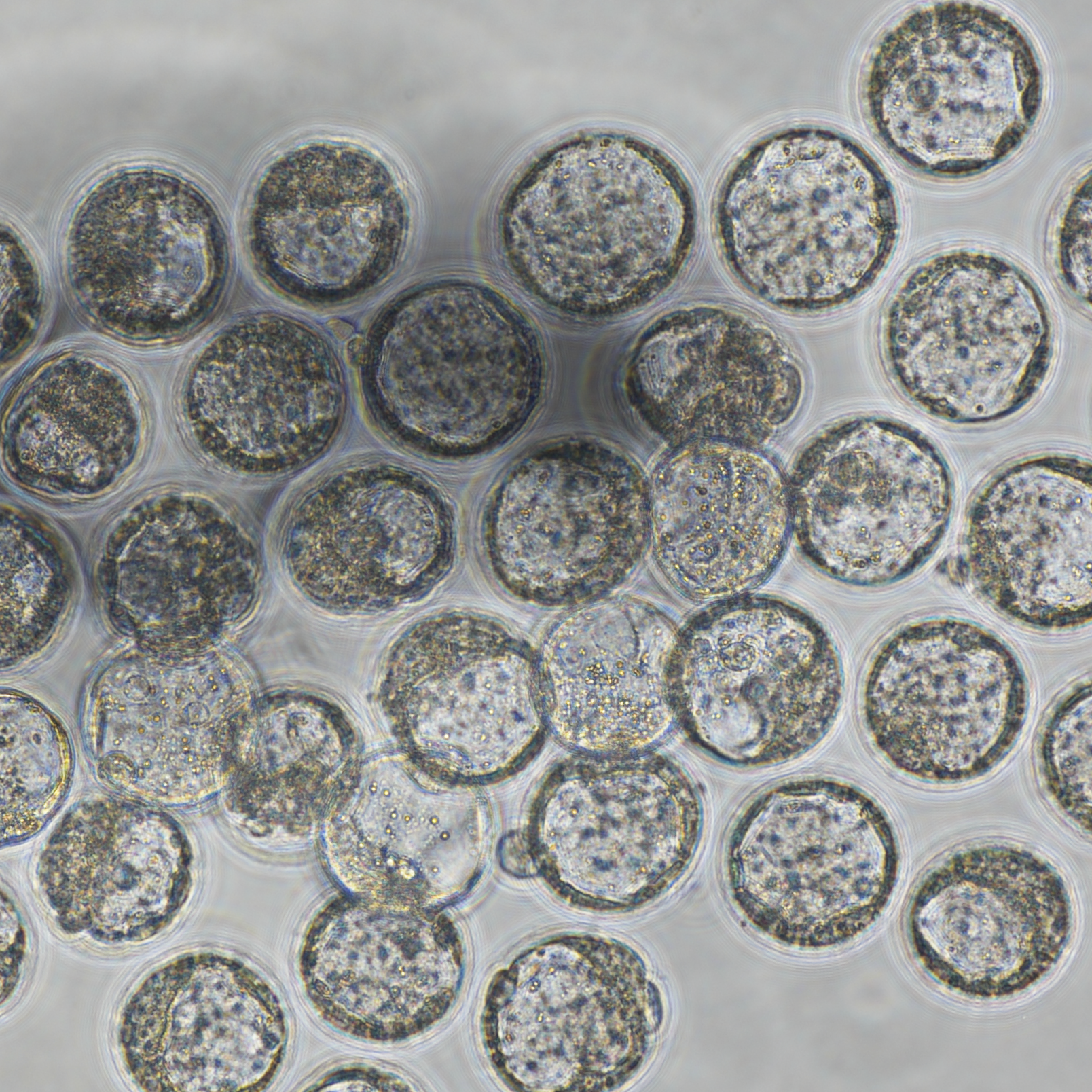

Supplement: Supplementary file 1 [file ijms-25-08658-s001.zip › seq6643.tiff]

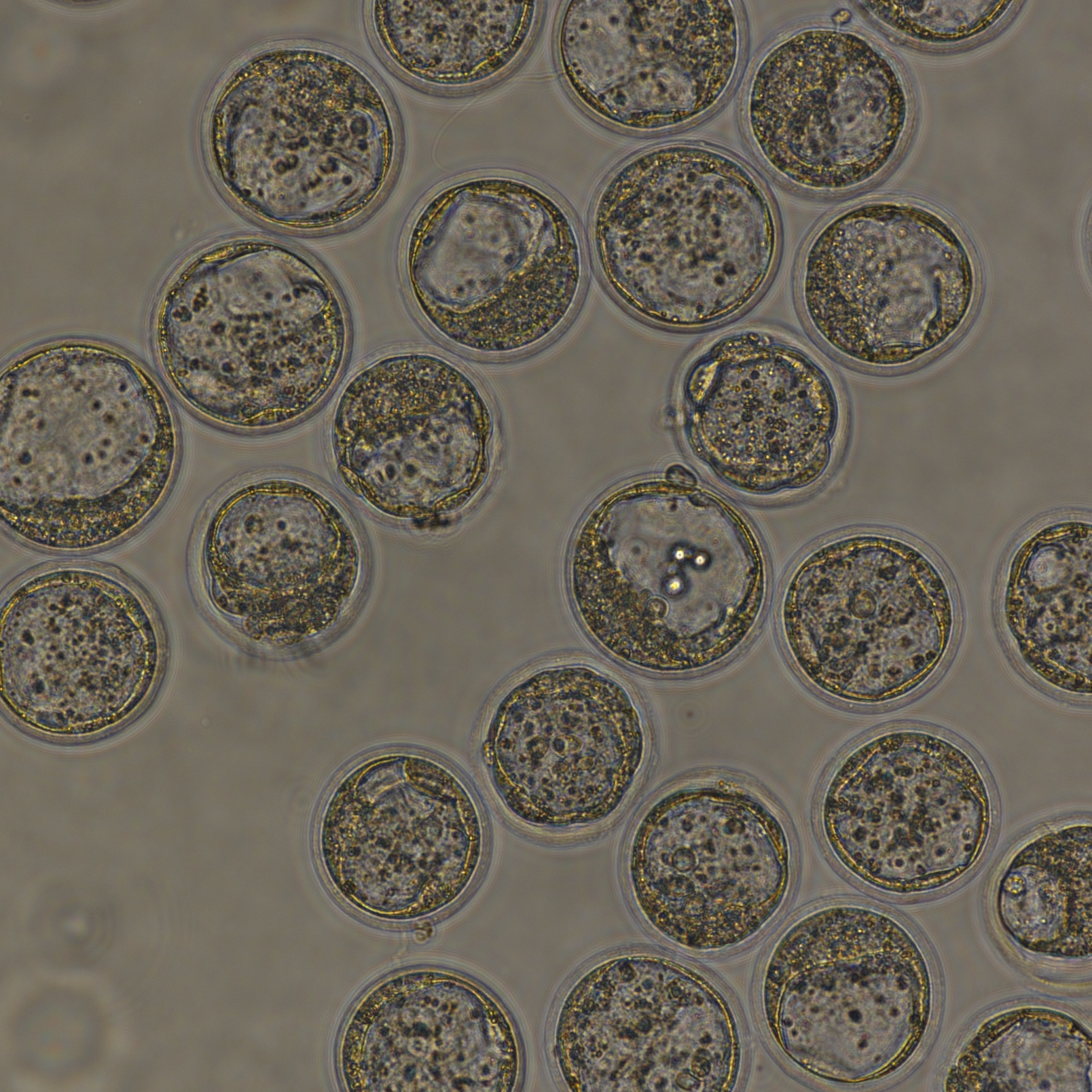

Supplement: Supplementary file 1 [file ijms-25-08658-s001.zip › seq6644.tiff]

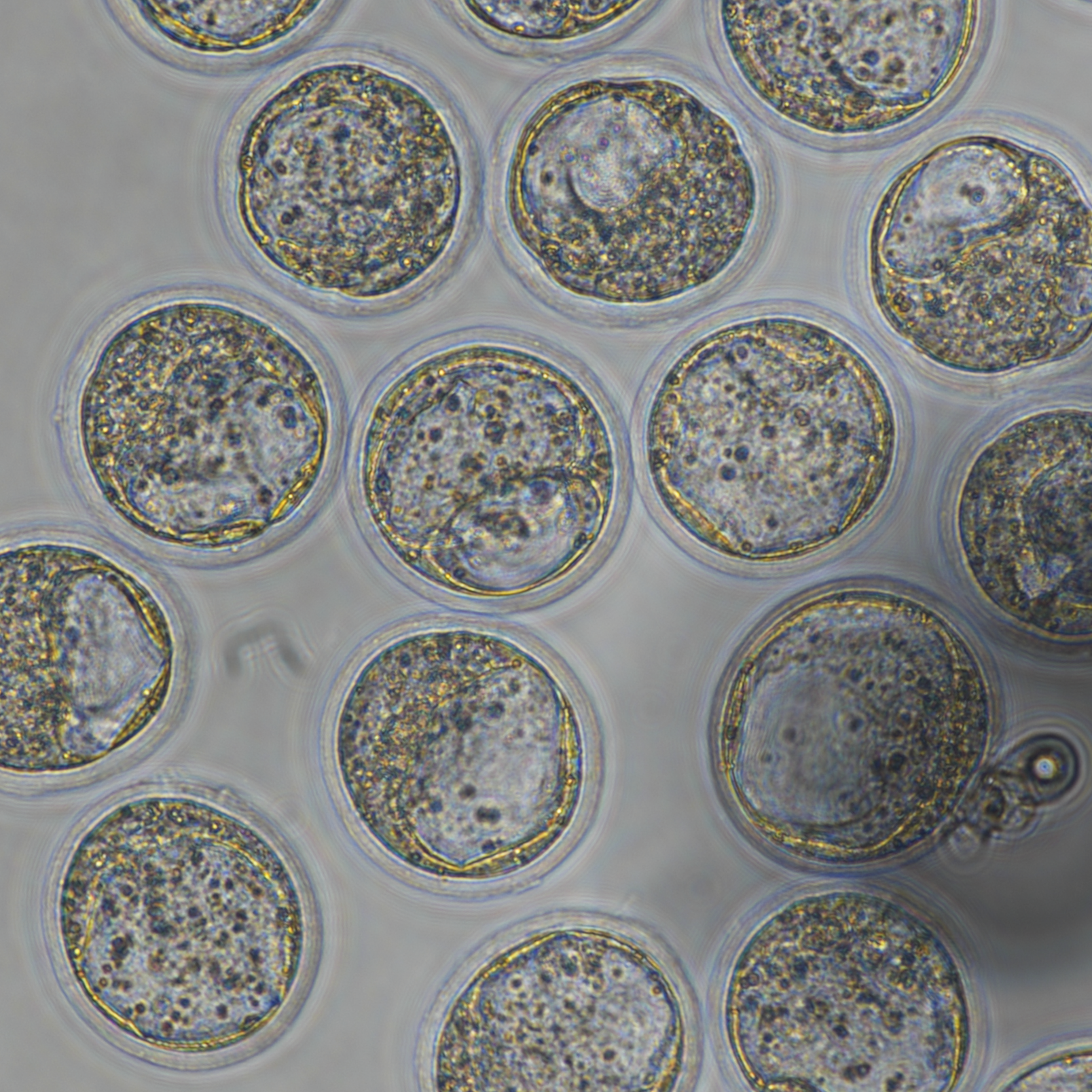

Supplement: Supplementary file 1 [file ijms-25-08658-s001.zip › seq6710.tiff]

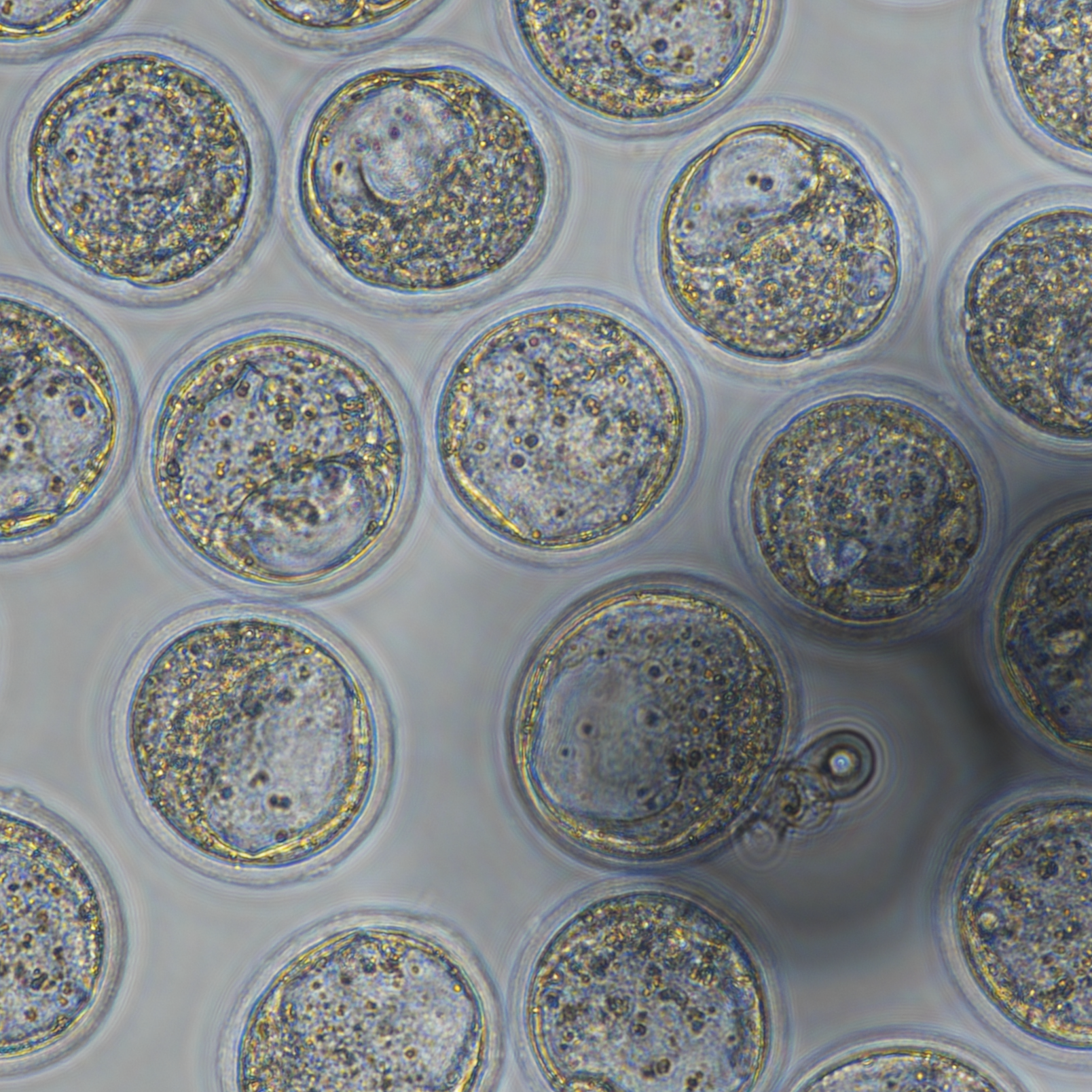

Supplement: Supplementary file 1 [file ijms-25-08658-s001.zip › seq6711.tiff]
